# Effects of alcoholic fermentation on the non-volatile and volatile compounds in grapefruit (*Citrus paradisi* Mac. cv. Cocktail) juice: A combination of UPLC-MS/MS and gas chromatography ion mobility spectrometry analysis

**DOI:** 10.3389/fnut.2022.1015924

**Published:** 2022-09-28

**Authors:** Xuedan Cao, Shuijiang Ru, Xiugui Fang, Yi Li, Tianyu Wang, Xiamin Lyu

**Affiliations:** Zhejiang Citrus Research Institute, Taizhou, China

**Keywords:** GC-IMS, metabolomics, grapefruit juice, alcoholic fermentation, flavonoids

## Abstract

Grapefruit has attracted much attention as a functional fruit, of which “Cocktail” is a special variety with low acidity. The present study aimed to investigate the effects of alcoholic fermentation on the non-volatile and volatile compounds of “Cocktail” grapefruit juice. To analyze, a non-targeted metabolomics method based on UPLC-MS/MS and volatiles analysis using GC-IMS were performed. A total of 1015 phytochemicals were identified, including 296 flavonoids and 145 phenolic acids, with noticeably increasing varieties and abundance following the fermentation. Also 57 volatile compounds were detected, and alcoholic fermentation was effective in modulating aromatic profiles of grapefruit juice, with terpenes and ketones decreasing, and alcohols increasing together with esters. Citraconic acid and ethyl butanoate were the most variable non-volatile and volatile substances, respectively. The results provide a wealth of information for the study of “Cocktail” grapefruit and will serve as a valuable reference for the large-scale production of grapefruit fermented juice in the future.

## Introduction

In recent years, fermented fruit juice has gained more attention due to its positive effect on human health (1–3). As an alternative to conventional thermal treatments, fermentation allows a low-energy consumption in the process and can affect the phytochemical composition and biological activity of fruit juice owing to the role of microbes (2, 4). As previously reported, lactic acid or alcoholic fermentation had a great impact on the bioactive compounds in citrus juices, such as flavonoids, phenolic acids, limonoids, carotenoids and vitamin A values (5). Additionally, alcoholic fermentation causes a variation in flavor substances, such as essential oils, sugars, lipids, ascorbic acid, and sulfur-containing compounds (6). Although existing studies suggest that alcoholic fermentation can alter the phytochemical composition of citrus juice and affects its function and flavor, however, they often focus on a small number of compounds and the systematic study of all components is needed.

The grapefruit (*Citrus paradisi* Mac.) originated in Barbados in the 18th century via crossing naturally between the pomelo (*Citrus maxima* Burm) and the sweet orange [*Citrus sinensis* (L.) Osbeck], which is now widely distributed in tropical and subtropical regions of the world (7). Grapefruit encompassed various bioactive chemicals like flavonoids, limonoids, carotenoids, organic acids, pectin, fiber, and folic acid (8–11). These bioactive substances endow grapefruit with important biomedical activities such as anti-inflammatory, antibacterial, anticancer, chemopreventive, and blood sugar regulation effects (12–14). Among different grapefruit varieties, “Cocktail” is a special one due to its higher sugar content and lower acidity in comparison with others (15). These properties make “Cocktail” grapefruit a promising raw material for fermented juice production. However, to our knowledge, research on “Cocktail” grapefruit juice and its fermented beverages has not been well studied. In particular, the effect of alcoholic fermentation on the non-volatile and volatile profiles of “Cocktail” grapefruit juice has not been reported.

Metabolomics is a comprehensive analysis, comprising the identification and quantification of as many metabolites as possible in a biological system or a food system (16). The application of this methodology is helpful to understand the relationship between food quality and processing. Wang et al. (17) have employed UPLC-MS/MS and gas chromatography-mass spectrometry (GC-MS) based metabolomics to explore the effects of high-hydrostatic-pressure and high-temperature treatments on the metabolic profiling of tomato juice. Citrus juice, in contrast, has more complex metabolic substances associated with human health that should be of well concern. Goh et al. (18) have applied LC-QTOF/MS to analyze non-volatile compounds in Hongxin and Shatian pomelo juices, and eight dominant compounds were identified and quantified. Likewise, Deng et al. (19) have identified and quantified 14 compounds in grapefruit using UHPLC system. These targeted approaches are more limited as reaching a relatively small number of metabolites, which is unsuitable for the overall metabolites analysis. By contrast, untargeted approach is a more common choice since it covers a wider range of molecules. As for volatile compounds, GC-MS and gas chromatography ion mobility spectrometry (GC-IMS) are extensively analysis tools. Comparatively, GC-IMS is less time-cost due to its ultra-high sensitivity and fast analytical speed (20). Brendel et al. (21) has achieved the differentiation of grapefruit juice and orange juice samples by their volatile profiles using a GC-MS-IMS system, and GC-IMS was found to be more sensitive in the identification of low-abundance volatiles compared with GC-MS. Therefore, GC-IMS technique has recently been applied to detect flavor compounds in fermented products, such as fermented Douchi (22) and alcoholic beverages like cherry wine (23). As a consequence, untargeted methods and GC-IMS are preferred for the overall study of non-volatile and volatile compounds of fruit juices. However, to our knowledge, few studies have adopted the integration of these two techniques to investigate the phytochemical composition and volatile compounds in grapefruit juice and its fermented juice.

In this study, metabolomics techniques based on UPLC-MS/MS and volatiles analysis using GC-IMS were performed to comprehensively analyze the variation of the metabolites and volatile compounds during grapefruit juice fermentation. The aim was to investigate the potential effects of alcoholic fermentation on the non-volatile and volatile substances of “Cocktail” grapefruit juices, which may contribute to the development of “Cocktail” grapefruit products.

## Materials and methods

### Chemical reagents

Gradient grades of methanol, acetonitrile, and formic acid were purchased from Merck Company (Germany). The internal standard 2-chlorophenylalanine was bought from J&K Chemicals Co., Ltd. (United States). Ultrapure water used was produced by a Millipore Milli-Q system (Millipore, Bedford, MA, United States). Pectinase (10000 U/g) and active dry yeast (*Saccharomyces cerevisiae* BV818) were provided by Jiangsu Ruiyang Biotech Co., Ltd. (Wuxi, China) and Angel Yeast Co., Ltd. (Yichang, China), respectively.

### Preparation of grapefruit juice and grapefruit fermented juice

Six-year-old grapefruit trees (*Citrus paradisi* Mac. cv. Cocktail) with similar growth and fruit-bearing capacity were selected, which were grown in the Zhejiang Citrus Research Institute experimental orchard located in Taizhou city, Zhejiang provinces, China (∼28°64′N, 121°16′E). Fruits were randomly harvested from these selected trees at commercial maturity during November to December, and immediately transported to the laboratory. A total of 100 kilograms of fruits with no physical injuries or infections were selected, and then the peels and seeds were removed manually. The grapefruit juice (FJ) sample was obtained by pulp homogenizing with a blender (FPM256, Kenwood, United Kingdom) and filtering through an 80-mesh filter, and stored at –80°C.

For fermentation, the fresh grapefruit juice was treated with 0.05% of pectolytic enzyme and kept at 45° for 1.5 h. After naturally cooled, the juice was inoculated with 200 mg/L of the yeast after the strain was activated in 37° water for 30 mins. The fermentation process lasted for 14 days using 25 L unsealed stainless steel containers, and the temperature was maintained between 18° and 20° (24). The grapefruit fermented juice (FMT) was obtained by discarding the lees and stored at –80°. Fermentation experiments were carried out in triplicate. Then the basic parameters of FJ and FMT were measured according to Castello et al. (1), with sugars and organic acids determined by HPLC methods (25). The quality parameters of FJ and FMT are presented in Table 1.

**TABLE 1:** Quality parameters of grapefruit juice (FJ) and grapefruit fermented juice (FMT).

Parameters	FJ	FMT
pH	3.62 ± 0.01^b^	3.81 ± 0.01^a^
Titratable acidity (g citric acid/100 mL)	0.85 ± 0.02^a^	0.54 ± 0.01^b^
Total soluble solids (°Brix)	10.82 ± 0.31^a^	4.37 ± 0.11^b^
Alcohol (% v/v)	0.38 ± 0.03^b^	6.01 ± 0.18^a^
**Sugars (mg/g)**		
Glucose	20.99 ± 1.05^a^	0.29 ± 0.01^b^
Fructose	27.19 ± 1.08^a^	1.94 ± 0.21^b^
Sucrose	59.42 ± 2.00^a^	nd^b^
**Organic acids (mg/g)**		
Oxalic	0.06 ± 0.01^a^	nd^b^
Tartaric	0.52 ± 0.03^a^	0.55 ± 0.03^a^
Malic	1.63 ± 0.17^b^	2.01 ± 0.14^a^
Shikimic	0.07 ± 0.01^a^	0.02 ± 0.01^b^
Lactic	nd^b^	0.23 ± 0.02^a^
Acetic	nd^b^	0.10 ± 0.04^a^
Citric	5.04 ± 0.26^a^	4.61 ± 0.24^a^
Quinic	nd	nd
Fumaric	nd	nd

Values are expressed as mean (n = 3) ± standard deviation (SD) and “n.d.” indicated no detection. Different letters in the same row represent a significant difference at p < 0.05.

### Metabolites analysis of the grapefruit juice and grapefruit fermented juice by UPLC-MS/MS

#### Metabolites extraction

After being thawed from the refrigerator at –80°C, the FJ and FMT samples were mixed with vortex for 10 s. As FMT samples contained much lower solid contents than FJ samples, the following extraction processes were slightly different in the two groups. For FJ, 9 mL sample was placed in a 50 mL centrifuge tube and immersed in liquid nitrogen for freezing, after which the sample was completely lyophilized using a SCIENTZ-100F lyophilizer (Xinzhi 100F, Ningbo, China). Then 50 mg of lyophilized FJ sample was taken into a 2 mL EP tube, and 1200 μL of 70% of methanol (containing 2-chlorophenylalanine as internal standard) was added for the extraction. As for FMT samples, 3 mL of thawed sample was taken for lyophilization as above, and then 200 μL extraction solution was added. Afterward, both FJ and FMT samples extraction procedure was performed by vortexing the sample for 15 min and centrifuging (12000 r/min, 4°C) for 3 min. The supernatants were filtered with a 0.22 μm membrane and stored at –80° for analysis. Note that the obtained data of the two groups were corrected according to the corresponding volume before we performed the comparison.

#### UPLC-MS/MS condition

The sample extracts were analyzed using a UPLC-MS/MS system (UPLC, SHIMADZU Nexera X2; MS, Applied Biosystems 4500 Q TRAP). The analytical conditions were as follows: for UPLC, the Agilent SB-C18 column (1.8 μm, 2.1 mm × 100 mm) was used and the column oven was set to 40°C. The mobile phase consisted of solvent A, pure water with 0.1% formic acid, and solvent B, acetonitrile with 0.1% formic acid. Sample separation was performed with a gradient program that employed the starting conditions of 95% A and 5% B. Within 9 min, a linear gradient to 5% A, 95% B was programmed, and a composition of 5% A, 95% B was kept for 1 min. Subsequently, a composition of 95% A and 5.0% B was adjusted within 1.1 min and kept for 2.9 min. The flow velocity was set as 0.35 mL/min and the injection volume was 4 μL. The effluent was alternatively connected to an ESI-triple quadrupole-linear ion trap (QTRAP)-MS.

ESI-Q TRAP-MS/MS condition: LIT and triple quadrupole (QQQ) scans were acquired on a triple quadrupole-linear ion trap mass spectrometer (Q TRAP), AB4500 Q TRAP UPLC-MS/MS System, equipped with an ESI Turbo Ion-Spray interface, operating in positive and negative ion mode and controlled by Analyst 1.6.3 software (AB Sciex). The ESI source operation parameters were as follows: ion source, turbo spray; source temperature 550°C; ion spray voltage (IS) 5500 V (positive ion mode)/−4500 V (negative ion mode); ion source gas I (GSI), gas II (GSII), curtain gas (CUR) was set at 50, 60, and 25.0 psi, respectively; the collision-activated dissociation (CAD) was high. Instrument tuning and mass calibration were performed with 10 and 100 μmol/L polypropylene glycol solutions in QQQ and LIT modes, respectively. QQQ scans were acquired as multiple reaction monitoring (MRM) experiments with collision gas (nitrogen) set to medium. A specific set of MRM transitions were monitored for each period according to the metabolites eluted within this period.

#### Statistical data analysis of metabolites

The metabolomics data of FJ and FMT were processed using the system software analyst (version 1.6.3 Applied Biosystems Company, Framingham, MA, United States). Metware database (MWDB) was adopted for substance characterization which was based on secondary spectrum information. Metabolite quantification was accomplished by MRM analysis of triple quadrupole mass spectrometry. After obtaining the metabolite spectrum analysis data of different samples, the mass spectrum peaks of all substances were integrated by peak area, and the mass spectrum peaks of the same metabolite in different samples were integrated and corrected. Then unsupervised principal component analysis (PCA) was performed by statistics function prcomp within R^1^ after the data was unit variance scaled. The heatmap of metabolites was carried out by the R package ComplexHeatmap. VIP (variable importance in projection) values were extracted from the OPLS-DA result, which also contained score plots and permutation plots generated using the R package MetaboAnalystR. Significantly regulated metabolites between groups were determined by VIP ≥ 1 and absolute log_2_FC (fold change) ≥ 1. The data was log-transformed and mean centering before OPLS-DA. To avoid overfitting, a permutation test (200 permutations) was performed. Identified metabolites were annotated using the KEGG Compound database^2^, and annotated metabolites were then mapped to the KEGG Pathway database^3^.

### Volatiles analysis by gas chromatography ion mobility spectrometry

#### Gas chromatography ion mobility spectrometry condition

Analyses for the identification of characteristic volatile compounds of fruit juice samples were performed on an IMS commercial instrument (Flavorspec^®^, GAS GmbH, Dortmund, Germany), equipped with an MXT-WAX column (30 m × 0.53 mm id, 0.1 μm film thickness, Restek, United States). For analysis, 2 g of each sample was placed into a 20 mL headspace vial, closed with magnetic caps, and incubated at 40°C for 20 min at 500 rpm/min. Afterward, 500 μL of the headspace gases was automatically injected into the GC-IMS equipment by a heated syringe (65°C). The nitrogen gas (N_2_) was used as carrier gas and drift gas, and the column temperature was kept at 60°C under isothermal conditions for timely separation. The programmed flow for carrier gas was set as follows: 2 mL/min for 2 min, then raised to 10 mL/min till 10 min, and ramped up to 100 mL/min in the next 10 min, then maintained at 100 mL/min until 30 min. The drift gas flow rate was held at 150 mL/min. Each analysis was conducted in triplicates.

#### Gas chromatography ion mobility spectrometry data analysis

The GC-IMS data were collected and processed using LAV software (version 2.2.1, G.A.S., Dortmund, Germany). To avoid significant errors in the multivariate statistical analysis, the spectra were normalized relative to the expected reaction-ion-peak (RIP) position, which was followed by spline interpolation to create a common set of points on the drift-time axis (*X*-axis) of the GC-IMS spectra. Volatile compounds were qualitatively analyzed by comparing the retention indexes and drift times with those in the GC-IMS library. The “Gallery Plot” plug-in of LAV software was used to automatically generate fingerprints, to visually and quantitatively compare the differences in volatiles between FJ and FMT samples. Principal component analysis and differential metabolites analysis were performed by R software.

## Results and discussion

### Quality parameters

The effect of alcoholic fermentation on the substances and parameters of grapefruit juice were presented in Table 1. The titratable acidity decreased by 36.5% after fermentation with an increase in the pH value, which may be owing to the consumption of some organic acids by the yeast as reported by Liu et al. (3). Likewise, the soluble solids content of the fermented juice showed a significant decrease due to the utilization of carbohydrates by the microbial. While a notable increase in alcohol content in fermented grapefruit juice was determined. Moreover, HPLC analysis was carried out to evaluate the changes in sugars and organic acids concentrations. Sucrose, fructose and glucose in the juice were mostly consumed by the yeast, and the consumption of sucrose was the highest among the sugars. Ordoudi et al. (26) reported that almost all the sugars in fruit juice were converted to alcohols at the end of alcoholic fermentation, which was consistent with our results. The concentration of citric acid, the main acid found in the juice, was slightly reduced after the fermentation. Lactic and acetic were detected as the newly formed acidic metabolites within the fermentation.

Organic acids and sugars are known as the most abundant solids present in fruit juice. They are responsible for the sour and sweet taste, and also have a significant effect on the mouthfeel quality. The consumption of sugars by the yeast sharply diminished the sweet taste and resulted in a relatively elevated sour taste of the juice. Since we have selected “Cocktail” grapefruit with lower acidity than other varieties, the fermented juice was more acceptable to consumers in terms of acidity. Besides, as a volatile acid, acetic acid can produce a pleasant acidity at levels below 0.8 g/L in fermented fruit juice (27). Small amounts of lactic acid produced by yeast metabolism can also improve the sensory properties of the juice (28). Taken our results together, the fermented “Cocktail” grapefruit juice is expected to meet the growing demand of consumers for fruit wines with less ethanol and good mouthfeel.

### Metabolomics analysis

#### Data quality assessment

A total of 1015 metabolites were identified in FJ and FMT samples. Supplementary Figure 1 represented the overlapping display of the mass spectra of mixed QC samples to ensure the reproducibility and reliability of the data. The results showed that the TIC curves of QC samples had a high overlap, indicating the reliability of the results.

#### Metabolomics analysis of grapefruit juice and grapefruit fermented juice

**FIGURE 1:**
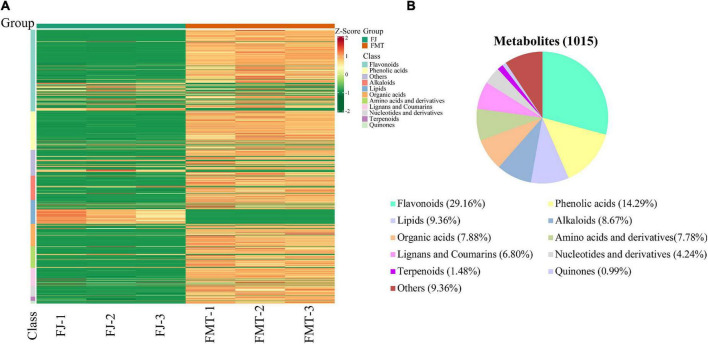
Heatmap visualization and proportions of the metabolites identified from grapefruit juice (FJ) and grapefruit fermented juice (FMT). **(A)** Heatmap visualization. The content of each metabolite was normalized to the complete linkage hierarchical clustering. Each example is visualized in a single column, and each metabolite is represented by a single row. Red indicates high abundance, whereas metabolites with low relative abundance are shown in green (the color key scale is right of the heat map). **(B)** Types and proportions of the metabolites identified from FJ and FMT.

The metabolite profiles of grapefruit juice samples can be visualized by the heatmap (Figure 1A). It showed that the three replicates of each group are clustered together, indicating good homogeneity and high reliability. The metabolites identified in FT and FMT can be classified into 10 major groups, namely flavonoids (296), phenolic acids (145), lipids (95), alkaloids (88), organic acids (80), amino acids (79), lignans and coumarins (69), nucleotides and their derivatives (43), terpenoids (15), and quinines (10). Overall, an obvious difference was found between FMT and FJ, and the abundance of most metabolites increased after alcoholic fermentation, including flavonoids, phenolic acids, amino acids and terpenoids. As shown in Figure 1B, the sum of flavonoids and phenolic acids accounted for more than 43% of the total identified compounds. Moreover, our result was in good agreement with previous literature in terms of the variation of flavonoids and phenolic acids after fermentation (29). The elevated content of flavonoids and phenolic acids may be partially owing to the hydrolysis of large original polymers performed by microbial enzymes. Besides, during the fermentation process, the alcohols and the heat generated in the matrix might contribute to the percolation of these bioactive compounds (30).

#### Multivariate analysis of identified metabolites in grapefruit juice and grapefruit fermented juice

**FIGURE 2:**
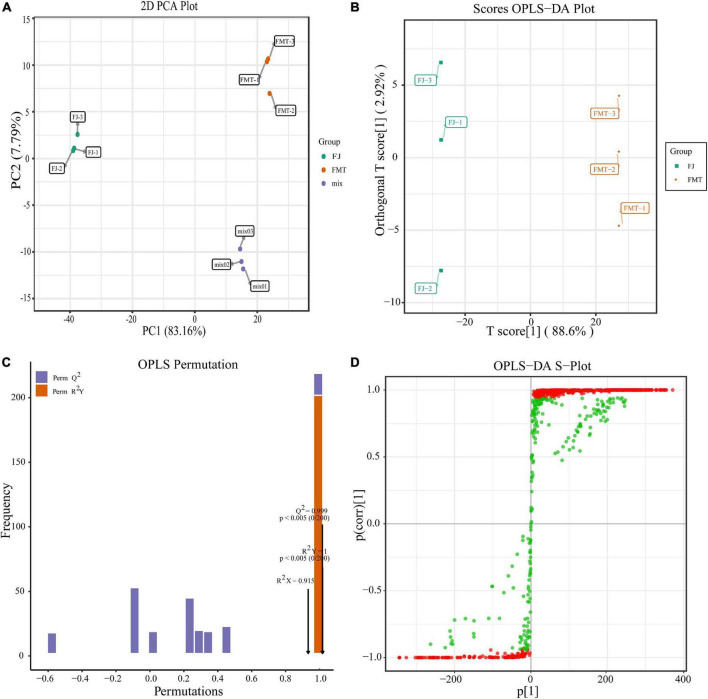
Multivariate analysis of identified metabolites. **(A)** PCA analysis of metabolites identified from FJ and FMT. Equal volumes of FJ and FMT samples were mixed as a quality control (QC). **(B)** OPLS-DA model plot of the metabolites identified from FJ and FMT. **(C)** Permutation test for OPLS-DA model validation of FJ and FMT. **(D)** OPLS-DA S-Plot of the samples.

To eliminate the effects of quantity on pattern recognition, we applied a log10 transformation of peak areas for each metabolite and, subsequently, performed PCA and OPLS-DA analysis. We selected the first two principal components, which can explain 85% of the total variation between groups. In the PCA scores, each point represents an individual sample, and Figure 2A showed that FJ and FMT were divided into two distinct areas, indicating the difference between FJ and FMT. In comparison to PCA, OPLS-DA is a better approach to processing two classes of datasets to discriminate the metabolites between the samples. As shown in Figure 2B, the horizontal coordinates indicate the scores of the main compounds, reflecting the differences between groups, and the vertical coordinates indicate the scores of the orthogonal components, showing the differences within groups. Accordingly, the OPLS-DA scores plot showed metabolites of the samples presented clear intergroup differences between FJ and FMT. In the model validation permutation test plot of OPLS-DA, the vertical coordinate indicates the accuracy of 200 models in 200 permutation tests, and the arrow indicates the location of the model accuracy (Figure 2C). Here, R2Y, Q2 and R2X values were higher than 0.9 and the *p*-Value is less than 0.05, showing the goodness of the prediction. In addition, the advantages of the S-plot from the supervised OPLS-DA can discriminate the differential constituents in the samples. In the S-plot, each point represents an ion detected using UPLC-MS/MS, where the *X*-axis represents variable contribution, and the *Y*-axis represents variable confidence. In Figure 2D, the variables that changed most significantly were plotted at the top or bottom of the “S” shape plot, where the red area indicated the VIP value of these metabolites ≥ 1 and the green section meant the VIP < 1. Collectively, both PCA and OPLS-DA analyses suggested that FJ and FMT had different metabolite profiles, indicating that the yeast activities had a great impact on the metabolites of grapefruit juice.

**FIGURE 3:**
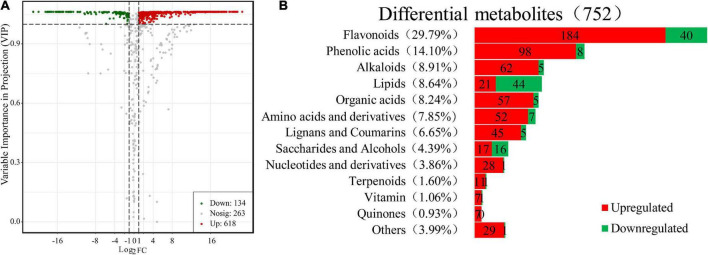
Differential metabolites between FJ and FMT. **(A)** Volcano plot of the 752 differential metabolites identified. Differential metabolites were defined as metabolites with VIP ≥ 1 and absolute log_2_FC (fold change) ≥ 1 in FMT relative to FJ. **(B)** Column chart depicting the biochemical categories of differential metabolites identified between FJ and FMT.

Moreover, further studies based on the VIP value, together with the fold change of the metabolites were carried out by a volcano plot. A total of 752 differential metabolites were identified with the fold change threshold > 2 (or <0.5) and VIP value threshold >1 between FJ and FMT, among which 134 metabolites were decreased and 618 metabolites increased (Figure 3A). The significant metabolites could be categorized into more than 10 different classes, including flavonoids, phenolic acids, alkaloids, lipids, organic acids, and amino acids and their derivatives, etc. (Figure 3B).

##### Flavonoids

Flavonoids have been shown to possess a variety of biological activities such as anti-inflammatory properties, cholesterol-lowering and immune system modulation (31). In the present study, 224 flavonoids were detected in the differential metabolites, accounting for approximately one-third of the total differentials, of which 184 increased and 40 decreased (Supplementary Table 1). The results indicated that fermentation has changed the flavonoids in grapefruit juice obviously. About 55 flavonoids were newly produced after fermentation, including 24 flavones, 14 dihydroflavones, 9 flavonols, 3 chalcones, 2 isoflavones, 1 dihydroflavonol, 1 flavonoid carbon glycoside, and 1 flavanone. The only 2 flavonoids that disappeared after fermentation were kaempferol-3-O-robinobioside and tricetin-5-O-(6″-malonyl) glucoside. The decline of kaempferol-3-O-robinobioside is likely due to the hydrolysis of glycosides by 3-O-glucosyltransferase produced by the yeast according to Wang et al. (32). Generally, most of the natural flavonoids in citrus exist in the form of glycosides (33). In this study, 137 kinds of flavonoids were identified as glycosides, and the most prominent glycosides in FJ are neohesperidin, hesperidin and naringin. Sicari et al. (34) analyzed the flavonoids in two different grapefruit juice and found naringin, narirutin, poncirin, and naringenin were the major flavonoids with a content ranging from 17.3 to 287.2 mg/L, which was similar to our results.

Previous reports showed that the absorption of flavonoid glycosides by intestinal epithelial cells is lower than flavonoid aglycone (35). And the bioavailability of flavonoid glycosides can be improved after being hydrolyzed into monoglycoside or aglycone forms during the fermentation (36). For instance, *saccharomyces cerevisiae* can degrade neohesperidin and hesperidin and form hesperetin and nobiletin with the action of glycosidases (37). In the present study, there was a significant decrease in flavonoid glycosides in FMT compared with FJ, such as neoeriocitrin, hesperidin, neohesperidin, narirutin, poncirin, didymin, rhoifolin, eriocitrin, isorhoifolin, naringin, while it was found concurrently an increase of their corresponding flavonoid aglycones. Additionally, flavonoids may undergo demethylation during alcoholic fermentation, as proved by the increased level of compounds like 5-demethylnobiletin and 3′-demethylnobiletin in FMT samples (38).

##### Phenolic acids

Phenolic acids are important bioactive substances in grapefruit juice, which played an important role in the juice function and sensory characteristics (39). The principal phenolic acids found in grapefruit are protocatechuic, neochlorogenic acid, hydroxycinnamic acids and ferulic acid, etc. (40). In this study, 106 kinds of phenolic acids and their derivatives were detected in the differential metabolites, of which 98 increased and 8 decreased after fermentation (Supplementary Table 1). Total 53 phenolic acids derivatives such as ferulic acid methyl ester and ethyl ferulate were newly generated, and only 1-O-p-coumaroyl-β-D-glucose and chlorogenic acid disappeared during the fermentation process. As reported by Zuriarrain-Ocio et al. (41), chlorogenic acid can be hydrolyzed by some lactic acid bacteria (LAB) strains to quinic and caffeic acids. Herein, we found the abundance decrease in chlorogenic acid corresponded to a large increase in caffeic acid, this may because *Saccharomyces cerevisiae* and LAB strains have similar conversion functions for chlorogenic acid. Among those ascending phenolic acids, such as salicylic acid, vanillic acid, protocatechuic acid and caffeic acid, have been reported to have antioxidant, antimutagenic, antiproliferative and antimicrobial properties (42). Additionally, newly generated methyl ferulate (FAME) had known function in inhibiting colony formation, inducing morphological change and apoptosis of cancer cells, and FAME also exhibited potential benefits to enhance the sensitivity of colorectal cancer cells to conventional chemotherapeutic drugs (43). Moreover, ethyl ferulate was reported to ameliorate LPS-induced acute lung injury in an AMPK/Nrf2-dependent manner (44). Consequently, the results implied that alcoholic fermentation can enrich bioactive phenolic metabolites in grapefruit juice and potentially improve its health value.

##### Lipids, organic acids, amino acids, and derivatives

Lipids were the only category in which more compounds were found to decrease, including palmitaldehydes, lauric acid and γ-linolenic acid, etc. Lipids are essential nutrients for yeast alcoholic fermentation, which play an important role in membrane structure, adaptation to stress, or as signaling molecules. Lipid metabolism also generates large intermediate products, for instance, lipid supplementation can greatly stimulate the formation of yeast volatile metabolites (45). As for organic acids, more than 90% of them increased in abundance after fermentation, with citraconic acid being the most abundant followed by aminomalonic acid. Some short-chain fatty acids were newly generated after yeast alcoholic fermentation, such as 2-hydroxybutyric acid, 4-acetamidobutyric acid, 2,3-dihydroxy-3-methylpentanoic acid, 3-methyl-2-oxobutanoic acid, and 2-hydroxyisobutyric acid, etc. Yao et al. (46) has reported short-chain fatty acids, produced by gut microbiome fermentation, played an important role in human immunity and metabolism. Additionally, the significantly increased 3-(4-hydroxyphenyl)-propionic acid in FMT, could inhibit the conversion of macrophages into foam cells by regulating cellular lipid metabolism, and suppressing cellular oxidative stress and inflammation (47). Moreover, fermentation caused an increase in the abundance of most amino acids, with L-cyclopentylglycine being the greatest after fermentation. The increase of free amino acids like L-methionine, L-leucine, L-isoleucine and L-tyrosine were mainly owing to the hydrolyzation of proteins by the proteolytic enzymes produced by the microbial. Also, the abundance of acylated amino acids ascended in the FMT sample, which may arise from the acylation of the respective amino acids activated by CoA or *N*-acylating enzymes (48).

In addition, vitamin C, an important micronutrient in citrus juice, was also significantly increased after fermentation with a fold change of 3.27. Overall, the abundance of most functional (flavonoids, phenolic acids) and nutritional components (amino acids, vitamins, organic acids, etc.) in fermented grapefruit juice increased, indicating that controlled alcoholic fermentation plays an important role in enhancing the nutritional value of grapefruit juice.

#### KEGG classification and enrichment analysis of differential metabolites

The pathway enrichment analysis of 752 differential metabolites was carried out by the KEGG database. A total of 199 differential metabolites were identified, which were distributed in 84 metabolic pathways (Supplementary Figure 2). Subsequently, we conducted the KEGG pathway enrichment analysis to identify differences in metabolic pathways between FJ and FMT (Supplementary Figure 3). The specialized metabolic pathways included flavonoid biosynthesis, pyrimidine metabolism, tropane, piperidine and pyridine alkaloid biosynthesis, valine, leucine and isoleucine biosynthesis, and phenylalanine metabolism, etc.

In particular, we focused on the changes of metabolites in the flavonoid synthesis pathway. According to Supplementary Figure 2, a total of 23 metabolites were annotated to the flavonoid biosynthesis pathway, accounting for 11.56% of the total metabolites annotated, which was the highest except for the metabolic pathway and biosynthesis of secondary metabolites pathway. Supplementary Figure 4 detailed the biotransformation involved in flavonoid biosynthesis, and the abundance changes of relevant compounds were notated. Many of the metabolites were monitored after fermentation with an upregulation (red). This is probably due to the biocatalytic action of newly generated enzymes by the yeast, such as flavonoid 3′,5′-hydroxylase, flavonoid 3′-monooxygenase, and flavonoid 4′-O-methyltransferase (32).

### Volatile profiles analysis

#### Visual topographic plot comparison of volatile compounds in grapefruit juice and grapefruit fermented juice

The results of the qualitative analysis of volatile compounds in the samples are listed in Table 2. Except for 8 unidentified compounds, a total of 57 components were tentatively identified from the GC-IMS library, containing 20 esters, 13 aldehydes, 8 terpenes, 7 ketones, 7 alcohols, 1 acid, and 1 ether. We detected more volatile compounds with GC-IMS in this study than in previous studies (49, 50), which helped to better understand the variation of volatiles before and after fermentation. In addition, several single compounds were found to produce multiple signals or spots (dimers, trimers, and even polymers). It has been reported that the formation of dimers or trimers is related to the high proton affinity or high concentration of the compounds in the analytes, and the compounds with high concentration could accelerate the combination of neutral molecules and proton molecules to form dimmers Lantsuzskaya (Krisilova) et al. (51).

**TABLE 2:** Qualitative analysis of volatile compounds in the grapefruit juice.

Class	Compound	CAS	Formula	MW	RI	Rt [sec]	Dt [RIP rel]
Aldehydes	Non-anal	C124-19-6	C_9_H_18_O	142.2	1401.7	1026.948	1.47202
	Octanal-M	C124-13-0	C_8_H_16_O	128.2	1299.3	822.172	1.40575
	Octanal-D	C124-13-0	C_8_H_16_O	128.2	1299.3	822.17	1.82885
	(E)-2-Hexenal	C6728-26-3	C_6_H_10_O	98.1	1232.2	715.257	1.18357
	Heptanal	C117-71-7	C_7_H_14_O	114.2	1197.3	665.432	1.33426
	2-Methyl-2-pentenal	C623-36-9	C_6_H_10_O	98.1	1165.7	603.152	1.50041
	Hexanal	C66-25-1	C_6_H_12_O	100.2	1102.5	488.97	1.56224
	Diethyl acetal	C105-57-7	C_6_H_14_O_2_	118.2	913.3	295.415	1.03213
	3-Methylbutanal	C590-86-3	C_5_H_10_O	86.1	933	308.829	1.41831
	Propanal-M	C123-38-6	C_3_H_6_O	58.1	820.2	239.525	1.04633
	Propanal-D	C123-38-6	C_3_H_6_O	58.1	820.2	239.525	1.14997
	2-Methylpropanal	C78-84-2	C_4_H_8_O	72.1	832.5	246.232	1.28485
	Butanal	C123-72-8	C_4_H_8_O	72.1	918.3	298.769	1.29337
	Furfural	C98-01-1	C_5_H_4_O_2_	96.1	1494	1254.592	1.09324
	Methacrolein	C78-85-3	C_4_H_6_O	70.1	896.2	284.265	1.04773
Esters	Ethyl nonanoate	C123-29-5	C_11_H_22_O_2_	186.3	1533.8	1367.68	1.53253
	Butyl hexanoate	C626-82-4	C_10_H_20_O_2_	172.3	1434.1	1101.743	1.4534
	Ethyl Acetate	C141-78-6	C_4_H_8_O_2_	88.1	903.1	288.708	1.33455
	Ethyl formate	C109-94-4	C_3_H_6_O_2_	74.1	881.1	274.736	1.21102
	Methyl acetate	C79-20-9	C_3_H_6_O_2_	74.1	851.2	256.851	1.19541
	Ethyl propanoate	C105-37-3	C_5_H_10_O_2_	102.1	975.1	339.569	1.45807
	Ethyl lactate	C97-64-3	C_5_H_10_O_3_	118.1	1359.8	937.574	1.13555
	Ethyl isobutyrate	C97-62-1	C_6_H_12_O_2_	116.2	984.5	346.834	1.56313
	Propyl acetate	C109-60-4	C_5_H_10_O_2_	102.1	994.4	354.659	1.47794
	Isobutyl acetate	C110-19-0	C_6_H_12_O_2_	116.2	1030.6	394.341	1.61424
	Methyl 3-methylbutanoate	C556-24-1	C_6_H_12_O_2_	116.2	1022.4	384.84	1.53899
	Ethyl butanoate	C105-54-4	C_6_H_12_O_2_	116.2	1051.9	420.051	1.56455
	Ethyl 3-methylbutanoate	C108-64-5	C_7_H_14_O_2_	130.2	1066	437.935	1.65116
	Ethyl 2-methylbutanoate	C7452-79-1	C_7_H_14_O_2_	130.2	1081.5	458.615	1.65179
	Isoamyl acetate	C123-92-2	C_7_H_14_O_2_	130.2	1139	551.952	1.74908
	3-methylbutyl propanoate	C105-68-0	C_8_H_16_O_2_	144.2	1187.5	648.433	1.83892
	Ethyl hexanoate-M	C123-66-0	C_8_H_16_O_2_	144.2	1246.2	736.379	1.33906
	Ethyl hexanoate-D	C123-66-0	C_8_H_16_O_2_	144.2	1244.7	733.969	1.80492
	Ethyl octanoate-M	C106-32-1	C_10_H_20_O_2_	172.3	1450.2	1140.763	1.48179
	Ethyl octanoate-D	C106-32-1	C_10_H_20_O_2_	172.3	1451.9	1145.019	2.03799
	Ethyl crotonate	C623-70-1	C_6_H_10_O_2_	114.1	1147	566.823	1.19847
	Isobutyl butyrate	C539-90-2	C_8_H_16_O_2_	144.2	1145.9	564.829	1.33362
Acids	Acetic acid-M	C64-19-7	C_2_H_4_O_2_	60.1	1500.7	1272.94	1.06007
	Acetic acid-D	C64-19-7	C_2_H_4_O_2_	60.1	1501.3	1274.602	1.15549
Ketones	2-Heptanone	C110-43-0	C_7_H_14_O	114.2	1187.7	648.824	1.6202
	Cyclopentanone	C120-92-3	C_5_H_8_O	84.1	1151.4	575.125	1.11402
	1-Penten-3-one-D	C1629-58-9	C_5_H_8_O	84.1	1041.4	407.196	1.31183
	1-Penten-3-one-M	C1629-58-9	C_5_H_8_O	84.1	1042.3	408.314	1.07757
	4-Methyl-2-pentanone-M	C108-10-1	C_6_H_12_O	100.2	1028.2	391.547	1.18121
	4-Methyl-2-pentanone-D	C108-10-1	C_6_H_12_O	100.2	1026.7	389.87	1.47794
	2-Pentanone	C107-87-9	C_5_H_10_O	86.1	1000.6	360.807	1.36862
	2-Butanone	C78-93-3	C_4_H_8_O	72.1	917.5	298.21	1.24368
	Acetone	C67-64-1	C_3_H_6_O	58.1	841.4	251.262	1.11874
Ethers	Dimethyl sulfide	C75-18-3	C_2_H_6_S	62.1	795.7	226.67	0.95972
Alcohols	1-Hexanol	C111-27-3	C_6_H_14_O	102.2	1372.5	963.788	1.33005
	2-Methyl-1-propanol-M	C78-83-1	C_4_H_10_O	74.1	1108.2	498.313	1.17198
	2-Methyl-1-propanol-D	C78-83-1	C_4_H_10_O	74.1	1108.5	498.856	1.35563
	1-Propanol-M	C71-23-8	C_3_H_8_O	60.1	1054.6	423.404	1.11164
	1-Propanol-D	C71-23-8	C_3_H_8_O	60.1	1056.8	426.198	1.25405
	Ethanol	C64-17-5	C_2_H_6_O	46.1	946.5	318.33	1.12584
	2-Propanol	C67-63-0	C_3_H_8_O	60.1	934.6	309.947	1.09034
	1-Butanol	C71-36-3	C_4_H_10_O	74.1	1161.8	595.425	1.37816
	3-Methyl-1-butanol	C123-51-3	C_5_H_12_O	88.1	1223.6	702.646	1.48018
Terpenes	γ-Terpinene	C99-85-4	C_10_H_16_	136.2	1261.4	759.891	1.22027
	Limonene-M	C5989-27-5	C_10_H_16_	136.2	1205.5	676.851	1.22414
	Limonene-D	C5989-27-5	C_10_H_16_	136.2	1204.8	675.81	1.29176
	Limonene-T	C5989-27-5	C_10_H_16_	136.2	1205.5	676.85	1.66077
	α-Phellandrene	C99-83-2	C_10_H_16_	136.2	1187.2	647.786	1.21834
	Myrcene-M	C123-35-3	C_10_H_16_	136.2	1175.9	623.912	1.22027
	Myrcene-D	C123-35-3	C_10_H_16_	136.2	1175.9	623.91	1.28789
	Myrcene-T	C123-35-3	C_10_H_16_	136.2	1175.9	623.91	1.711
	β-Pinene-M	C127-91-3	C_10_H_16_	136.2	1132.9	540.871	1.21834
	β-Pinene-D	C127-91-3	C_10_H_16_	136.2	1132.9	540.87	1.63758
	(-)-β-Pinene	C18172-67-3	C_10_H_16_	136.2	1118.1	514.921	1.21834
	α-Pinene-M	C80-56-8	C_10_H_16_	136.2	1035.3	399.93	1.22096
	α-Pinene-D	C80-56-8	C_10_H_16_	136.2	1034.4	398.81	1.66677
	Terpinolene	C586-62-9	C_10_H_16_	136.2	1299.5	822.682	1.20762

RI and Rt mean retention index and retention time, respectively. Dt indicates migration time, where [RIP rel] refers to normalization treatment.

**FIGURE 4:**
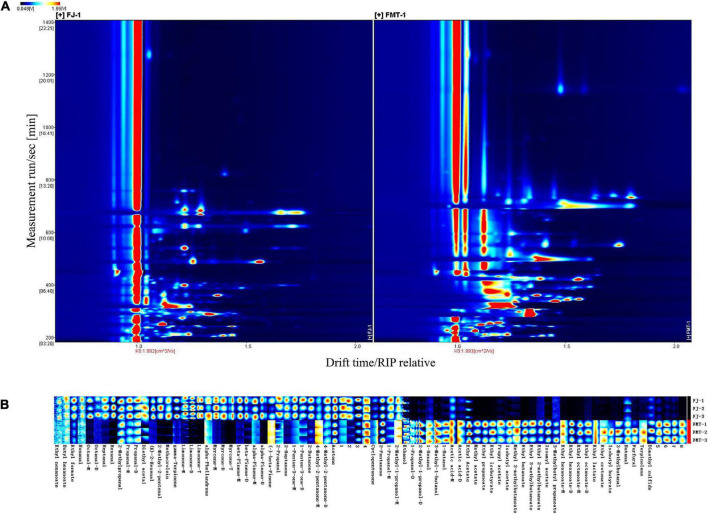
Gas chromatography-mass spectrometry spectrum of the FJ and FMT samples (**A**, a top view) and Gallery plot fingerprint of volatile substances **(B)**.

Figure 4A shows GC-IMS topographical plots of volatile compounds in grapefruit juice before and after fermentation. The ordinate represents the retention time of volatile compounds during GC separation, and the abscissa represents the ion migration time (after normalization). The red vertical line on the left is the reactive ion peak (RIP), each point on the right of the RIP represents a volatile compound extracted from the samples. The color and area of the signals represent the signal intensity of the compounds. Darker colors and larger area spots indicate that the content of the volatile substances was higher. As shown, most of the signals appear in the retention time of 200 to 800 s and the drift time of 1.0 to 1.8 ms. It was observed the volatile compounds changed dramatically after fermentation.

The contribution of volatile aroma compounds to the overall composition and sensory perception of fruit wines is well recognized. To further analyze the specific effects of fermentation on the volatile flavor substances of grapefruit juice, fingerprint profiles of volatiles in FJ and FMT were generated according to the Gallery Plot plug-in (Figure 4B), enabling an intuitive comparison of the differences in volatile compounds between the two groups of samples. Each row of the graph represents the signal peak of a volatile compound contained in one sample, and each column indicates the comparison between different samples for the same volatile compound. The shade of the color represents the content of the volatile compound, the brighter the color, the higher the content and vice versa (the numbered peaks are those not identified). As seen in Figure 4B, FT samples showed high contents of ethyl nonanoate, butyl hexanoate, ethyl formate, nonanal, octanal, heptanal, hexanal, 2-methylpropanal, propanal, acetal, E-2-hexenal, 2-methyl-2-pentenal, methacrolein, γ-pinene, limonene, α-phellandrene, myrcene, β-pinene, α-pinene, (-)-β-pinene, 2-propanol, 2-heptanone, 1-penten-3-one, 2-butanone, 4-methyl-2-pentanone, and acetone, which could form the flavor profile of fresh grapefruit juice. Compared with FT, FMT samples showed less content in aldehydes like 2-methyl-2-pentenal, hexanal, heptanal as well as terpenes like limonene, myrcene and β-pinene, while showed higher content in esters such as ethyl butanoate, ethyl propanoate, ethyl isobutyrate, methyl acetate and alcohols like ethanol, 3-methyl-1-butanol and 1-butanol.

#### Principal component analysis and OPLS-DA analysis

**FIGURE 5:**
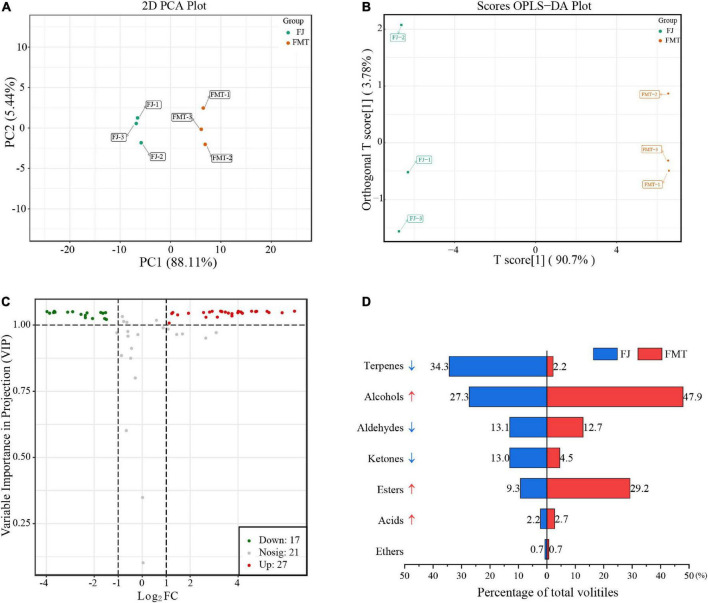
Multivariate analysis of volatile compounds. **(A)** PCA analysis of volatile compounds from FJ and FMT. **(B)** OPLS-DA model plot of the volatile compounds from FJ and FMT. **(C)** Volcano plot of differential metabolites. **(D)** Volatile substances changes of different categories between FJ and FMT.

Principal component analysis of volatiles was used to analyze the difference between FJ and FMT samples (Figure 5A). Based on the first principal component (PC1 = 88.11%) and the second principal component (PC2 = 5.44%), two groups were separated clearly, indicating that fermentation can alter the volatile composition of grapefruit juice. A comparison of the volatile characteristics of FJ and FMT using the OPLS-DA model is shown in Figure 5B. As calculated, R2Y of the model was 1, Q2 and R2X were both greater than 0.9, and the *p*-Value is less than 0.05, indicating that the model is of goodness. As a result, both PCA and OPLS-DA results indicated the flavor profiles of grapefruit juice changed dramatically before and after fermentation.

#### Effect of fermentation on the volatiles in grapefruit juice

The effect of fermentation on grapefruit juice is reflected in the total amount and composition of volatile substances. According to volatile profiles abundance, the total amount of volatiles after fermentation was higher than unfermented juice, increasing about 80% compared to FJ samples. In addition, the composition of the volatiles has changed obviously *via* fermentation (Figure 5C). Terpenes accounted for the highest percentage in FJ, followed by alcohols. In contrast, the highest volatiles in FMT were alcohols, followed by esters, and terpenes accounting for only 2.2% (Figure 5D). Fan et al. (52) also observed significant changes in volatile compounds in orange juice after fermentation, with most terpenes disappearing after fermentation and esters being the most abundant aromatically compounds. For these phenomena, it was suggested the terpenes decrease may be owing to the transformations by yeast. For example, limonene could be transformed to carveol, trans-2,8-methadien-1-ol, and cis-2,8-methadien-1-ol by yeast via hydroxylation, and the simple adsorption by yeast cells may also result in the massive loss of aroma compounds during the fermentation process (53). In addition, monoterpene and sesquiterpene hydrocarbons were primarily distributed in the pulp (74.0 and 87.2%) and cloud (7.3 and 14.9%) of citrus juice (54), while the insoluble cloud and pulp turned into a precipitate after alcoholic fermentation. Herein, the FMT was obtained by collecting the supernatants of fermented juice without the precipitation, in which some terpenes may also be lost together. The only exception is the increase in terpinolene content after fermentation. Terpinolene has been reported not to be degraded or transformed by the yeast (53), and previous studies showed it can be also derived from the decomposition of limonene (55), which may account for its raised level. Given that the flavor of grapefruit juice depends on the combined effects of multiple aroma compounds, the analysis of these volatiles compounds is summarized by category as follows.

##### Terpenes

Terpene was one of the most abundant volatiles in grapefruit juice, and limonene exhibited the highest concentration in both FJ and FMT, which agreed with the results of the previous study (56). As reported, most of the detected terpenes imparted citrusy, fruity, piney and woody notes. For example, limonene and α-pinene both contribute citrus-like, pungent aromas (57). β-pinene was described as woody and piney, while myrcene contributed most of the green, fruity notes. Fermentation caused a dramatic decrease in terpenes and may result in a reduction of citrusy flavor in juice. Nevertheless, terpenes may contribute to the overall flavor to a smaller extent than other ingredients such as esters and alcohols, due to the relatively higher sensory threshold (57). Thus, the reduction of terpenes content may have a limited effect on the aroma. In contrast, compounds such as esters, organic acids and alcohols with low sensory thresholds may have a greater impact on juice flavor.

##### Esters

A total of 20 esters were detected in grapefruit juice, which were known to contribute to the “top note” of fruit and citrus flavors (58). Controlled alcoholic fermentation can increase the content of juice esters, owing to the generated by-products that were produced in the yeast decomposition of sugars. After fermentation, the proportion of esters in the volatiles of grapefruit juice increased from 9.3% (FJ) to 29.2% (FMT). Among these esters, ethyl acetate was the most abundant compound both before and after fermentation. Ethyl butyrate, ethyl hexanoate, methyl acetate, ethyl propanoate, ethyl isobutyrate, propyl acetate, isobutyl acetate and isoamyl acetate all showed a content increase in FMT samples. As reported, isoamyl acetate confers a banana aroma characteristic and has a high aggregate value for the food industry (59). Ethyl butyrate and ethyl hexanoate are responsible for the fruity odor and act as important contributors to the desired flavor in orange products (58). Selli et al. (60) identified 63 compounds in Turkish Kozan wine and found that alcohols, followed by esters, were the most abundant aromatic compounds in orange wine. Uniquely, the content of methyl acetate was high in fresh juice with only less than ethyl acetate, yet showed a decline after fermentation, implying that yeast could also consume or degrade some esters during the growth. Ethyl formate has been reported to have antimicrobial activity (61), while yeast may achieve suitable growth conditions via the degradation of ethyl formate. Although there is no direct evidence to confirm this speculation, the reports that ethyl formate could be hydrolyzed into formic acid and ethanol by *Aeromonas salmonicida* and the formed formic acid would participate in the cyclic metabolism of carboxylic acid (62), which may provide some information.

##### Alcohols

The concentration of alcohols in FMT was much higher than that in FJ, indicating that the fermentation resulted in a huge change in the alcoholic aroma. Ethanol was the highest alcohol both in FJ and FMT, imparting the sweet and wine notes for the juices. Likewise, 1-Propanol contributed to the sweet and wine notes, while 3-methyl-1-butanol (isoamylol), 1-butanol and 1-hexanol enhanced the fruity note. Interestingly, alcohols could contribute to the absorption of phenolic compounds in the human intestine, in addition to enhancing the flavor of the juice (1). Combined with the former result that alcoholic fermentation can increase the abundance of flavonoids and phenolic acids in the juice, it was suggested that fermented grapefruit juice can be served as a wellness drink to promote human health.

##### Acids, aldehydes, and ketones

Concerning other volatile constituents, including acids, aldehydes and ketones, it was found that only one volatile acid, i.e., acetic acid was detected from the current samples. Acetic acid content was higher in FMT than that in FJ samples, which was similar to a previous report (63). A total of 14 aldehydes were detected in the juice, and the concentrations of 8 aldehydes showed a dramatic decrease after fermentation, including octanal, heptanal, 2-methyl-2-pentenal, (E)-2-hexenal, hexanal, diethyl acetal, propanal and methacrolein. The reduction was likely due to oxidation and/or enzymatic degradation occurring during yeast fermentation (30). For example, propanal can be oxidized to produce propionic acid and utilized by the yeast, and aldehydes and ketones would be transformed into corresponding biogenic amines by the microbial enzymes (63). On the other hand, 4 aldehydes increased after fermentation, namely 3-methylbutanal, butanal, 2-methyl-1-propanol and furfural, which may be the oxidation products of alcohols with the raising level. Seven ketones were detected, five of which decreased significantly, including 2-heptanone (fruity, spicy and woody), 4-methyl-2-pentanone (green, herbal, fruity), 1-penten-3-one (fresh and pungent), 2-butanone (fruity) and acetone (apple and pungent). Among them, 1-penten-3-one has been characterized as an impact aroma compound in grapefruit, orange juice, olive oil, or tomatoes due to a very low odor threshold of 0.94 μg/L (64). However, 1-penten-3-oneis prone to degradation due to its instability in the heat treatment or storage progress (64).

Collectively, fermented grapefruit juice had a reduction in the abundance of most terpenes and ketones contributing to “fruity,” “citrus-like” notes, and the increase of the esters with “green,” “fruity” notes. In addition, the newly generated esters and alcohols could endow the fermented juice with fruitiness and wine flavor. It indicated that alcoholic fermentation induced changes in the aroma profile and enriched the flavor of grapefruit juice. The study is a comprehensive analysis of the functional and volatile compounds in fermented grapefruit juice, yet still requires refinement in the next work. Besides, more efforts should be made to perform the functional evaluation of fermented grapefruit juice *in vitro* and *in vivo* models, aiming to obtain more information on its efficacy.

## Conclusion

In this study, an abundant number of metabolites were generated from “Cocktail” grapefruit juice fermented by *Saccharomyces cerevisiae*. Metabolites in grapefruit juice after alcoholic fermentation can be effectively evaluated by wide-target metabolomics, and GC-IMS allows a well-visualized differentiation between fermented and unfermented grapefruit juice. The integration of these two techniques achieves a comprehensive analysis of non-volatile and volatile metabolites in grapefruit juice, which provides a wide perspective to further understand or evaluate the effect of alcoholic fermentation on the biochemical composition of citrus juices. Grapefruit juice was identified to be rich in flavonoids and phenolic acids, and fermentation can lead to a notable increase in the variety and abundance of these bioactive components. Meanwhile, Lipids, organic acids, amino acids and other classes of compounds also underwent significant changes. Additionally, alcoholic fermentation was effective in modulating grapefruit aromatic profiles, especially in the enrichment of juice aroma compounds with the “fruity” notes (ethyl butyrate, ethyl hexanoate, isoamyl acetate, 1-butanol, etc.) and “winey” notes (ethanol, 1-propanol, 1-hexanol, etc.). It is expected that the obtained results will serve as a valuable reference for the large-scale production of grapefruit fermented juice and its functional enhancements in the future, as well as inspiring research on the formation mechanism of key flavor substances in the juice and the transformation process of major functional compounds during fermentation.

## Data availability statement

The original contributions presented in this study are included in the article/Supplementary material, further inquiries can be directed to the corresponding authors.

## Author contributions

XC, XF, and SR designed the study. XC, YL, and TW collected the data and participated in the design of the experimental. XL participated in manuscript preparation and revision. All authors contributed to the article and approved the submitted version.

## Footnotes

1
www.r-project.org

2
http://www.kegg.jp/kegg/compound

3
http://www.kegg.jp/kegg/pathway.html
