# Supplementary material for: Effects of alcoholic fermentation on the non-volatile and volatile compounds in grapefruit (Citrus paradisi Mac. cv. Cocktail) juice: A combination of UPLC-MS/MS and gas chromatography ion mobility spectrometry analysis
Source: Front Nutr. 2022 Sep 28;9:1015924. doi: 10.3389/fnut.2022.1015924 (PMC9554462; doi:10.3389/fnut.2022.1015924)
Supplement: Supplementary file 1 [file Data_Sheet_1.PDF]

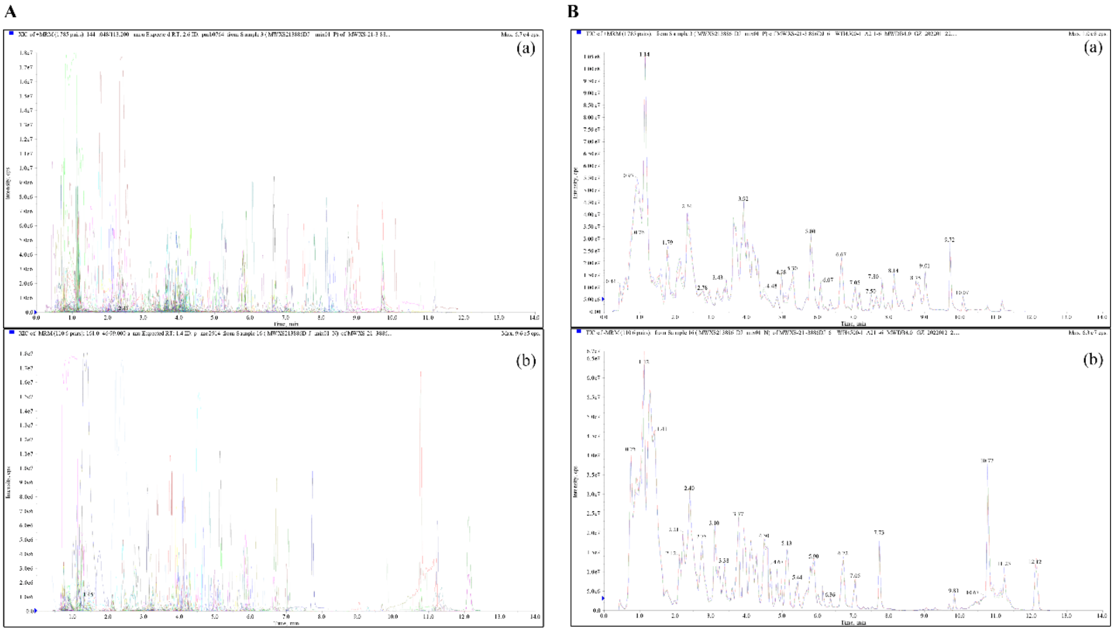

Fig. S1. Detection of metabolites with LC-MS/MS. (A) Multipeak map of MRM metabolite detection (ion current spectrum of multi-material extraction, XIC). (B) Total ion current (TIC) overlapping map of QC samples mass spectrometry results. Note: The abscissa is the retention time of the metabolite detection (retention time, Rt). The ordinate is the ion current intensity of the ion detection (the intensity units are counts per second (cps)). (a) is the detection in positive ion mode; (b)is the detection in negative ion mode.

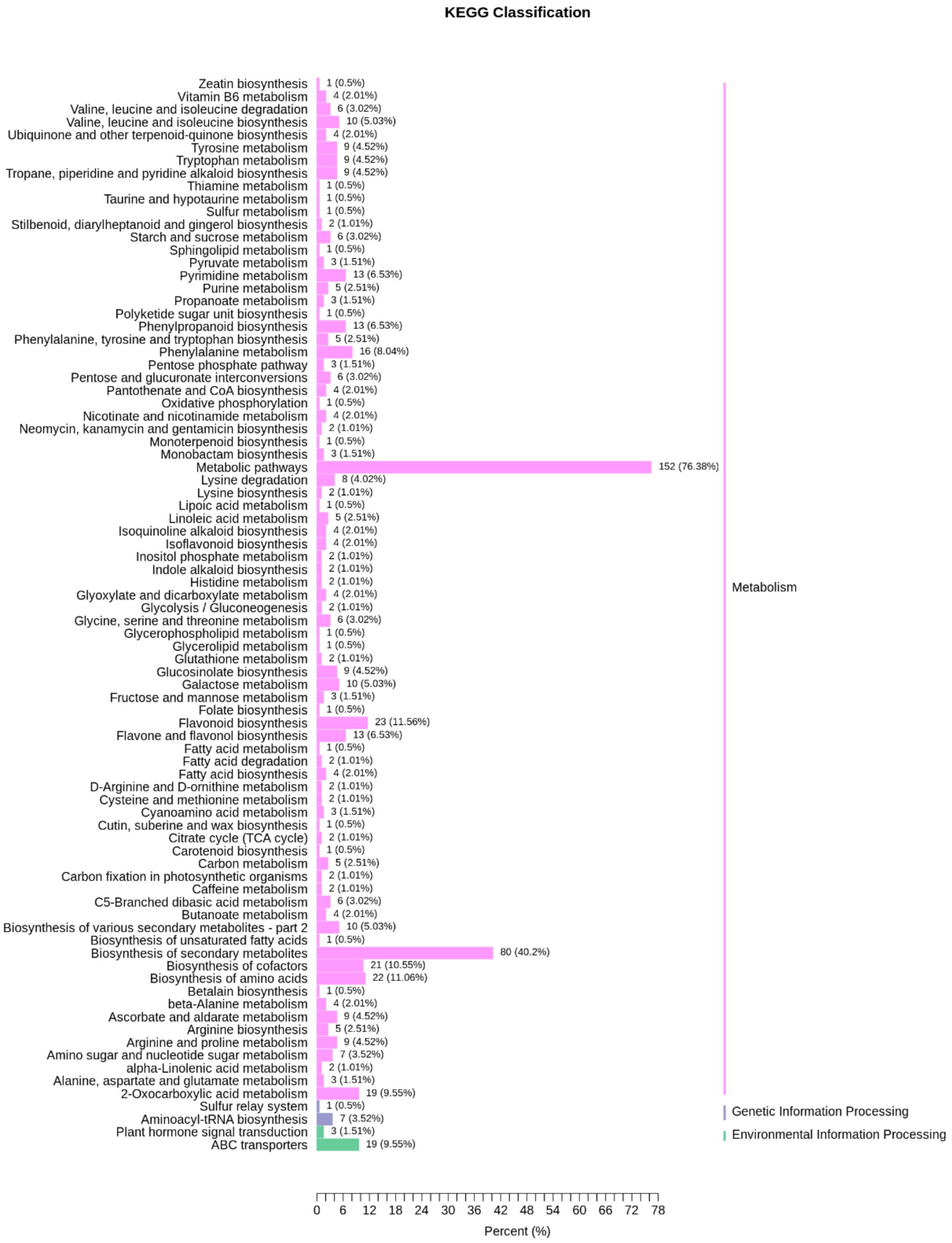

Fig. S2. KEGG classification of metabolites in FJ and FMT. The vertical coordinate is the names of the KEGG metabolic pathways, and the horizontal coordinate is the number of metabolites annotated to the pathway and their number as a percentage of the total number of annotated metabolites.

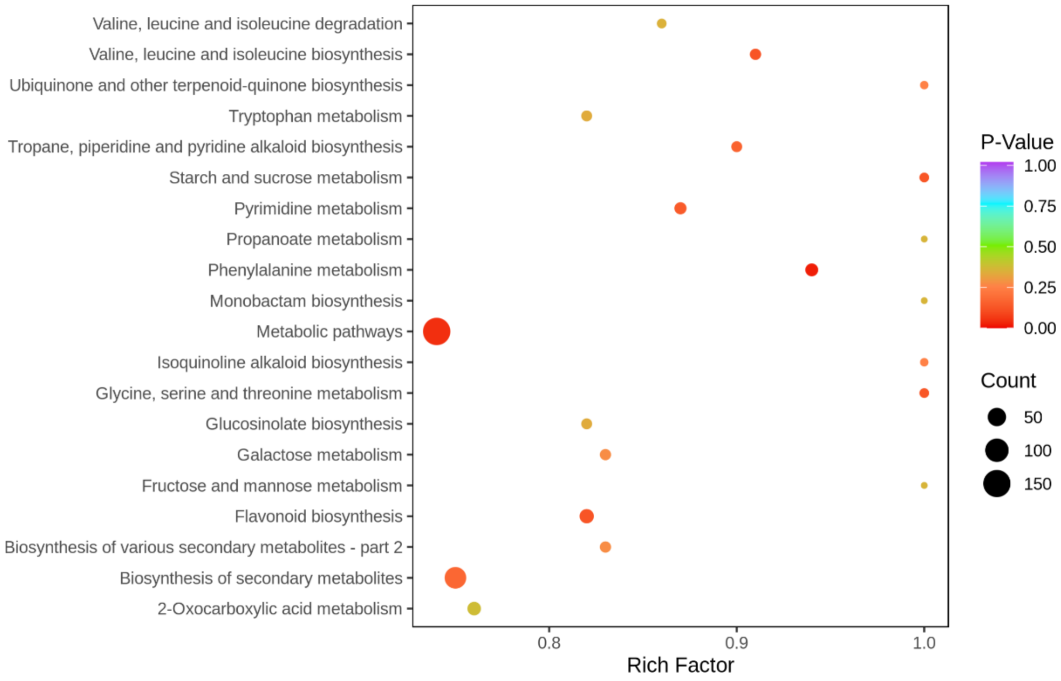

Fig. S3. Enrichment map of differential metabolites. The dot size represents the number of enriched metabolites in the pathway. The color of the dot reflects the p-value, where red indicates more significant enrichment.

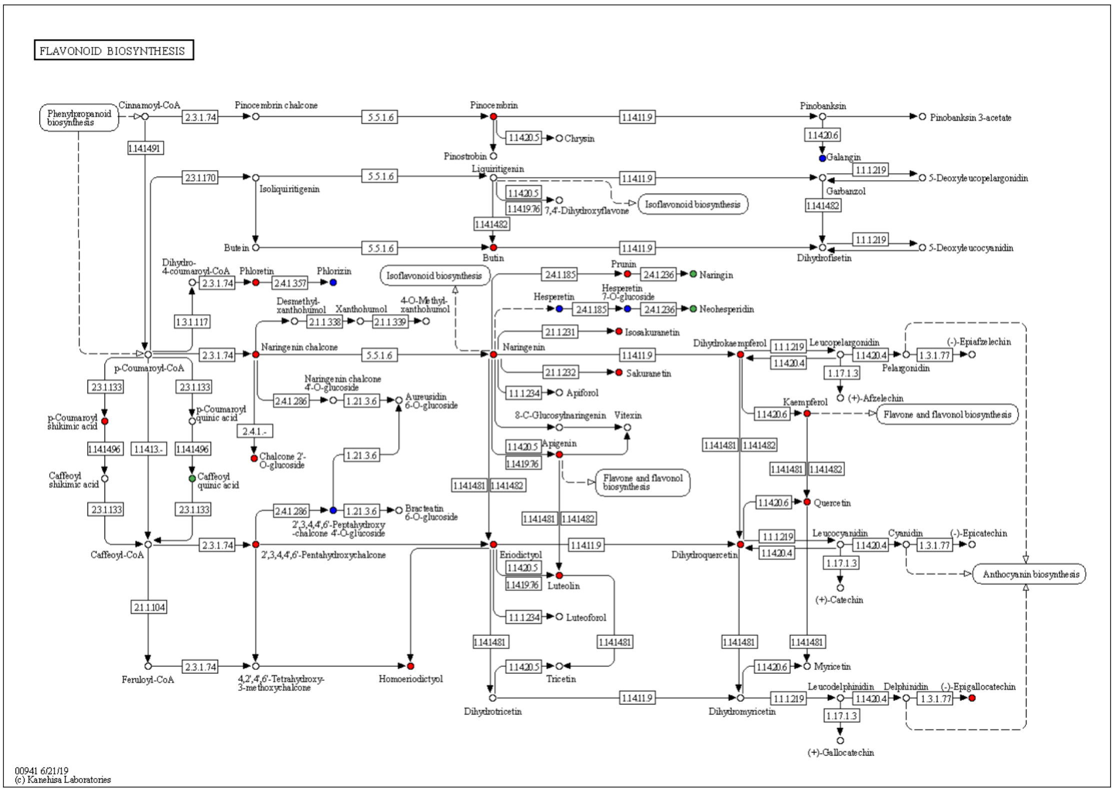

Fig. S4. The KEGG pathway diagram of flavonoid biosynthesis. The red color represents up-regulation, green color represents down-regulation and blue color indicates no significant difference. The full line represents direct conversion and the dotted line represents indirect conversion.
