# Supplementary material for: Effects of alcoholic fermentation on the non-volatile and volatile compounds in grapefruit (Citrus paradisi Mac. cv. Cocktail) juice: A combination of UPLC-MS/MS and gas chromatography ion mobility spectrometry analysis
Source: Front Nutr. 2022 Sep 28;9:1015924. doi: 10.3389/fnut.2022.1015924 (PMC9554462; doi:10.3389/fnut.2022.1015924)
Supplement: Supplementary file 2 [file Table_1.pdf]

Table S1. Differential metabolites between FJ and FMT samples

No.	Compounds	Molecule Weight	Formula	Ionization model	FJ-1	FJ-2	FJ-3	FMT-1	FMT-2	FMT-3	VIP	Fold Change	Type
**Alkaloids**													
1	Piperidine	8.51E+01	C5H11N	[M+H]+	8.54E+05	7.72E+05	7.99E+05	1.36E+07	1.24E+07	1.27E+07	1.06E+00	1.59E+01	up
2	Putrescine	8.81E+01	C4H12N2	[M+H]+	3.78E+05	3.17E+05	3.50E+05	9.02E+05	1.01E+06	9.55E+05	1.06E+00	2.74E+00	up
3	Choline	1.03E+02	C5H13NO	[M+H]+	1.46E+07	1.54E+07	1.20E+07	5.90E+06	5.05E+06	5.27E+06	1.04E+00	3.86E-01	down
4	Betaine	1.17E+02	C5H11NO2	[M+H]+	9.00E+00	9.00E+00	9.00E+00	2.26E+05	2.30E+05	1.51E+05	1.06E+00	2.25E+04	up
5	N-Benzylmethylene isomethylamine	1.19E+02	C8H9N	[M+H]+	9.75E+04	1.01E+05	1.10E+05	9.43E+05	7.47E+05	9.80E+05	1.06E+00	8.63E+00	up
6	2-Phenylethylamine	1.21E+02	C8H11N	[M+H]+	9.00E+00	9.00E+00	9.00E+00	8.20E+05	7.71E+05	7.39E+05	1.06E+00	8.63E+04	up
7	6-Deoxyfagomine	1.31E+02	C6H13NO2	[M+H]+	8.87E+05	7.70E+05	8.31E+05	1.25E+07	1.25E+07	1.24E+07	1.06E+00	1.50E+01	up
8	2-Phenylacetamide	1.35E+02	C8H9NO	[M+H]+	1.55E+06	1.18E+06	1.25E+06	1.34E+07	1.19E+07	1.27E+07	1.06E+00	9.53E+00	up
9	N-benzylformamide	1.35E+02	C8H9NO	[M+H]+	1.52E+06	1.19E+06	1.18E+06	1.31E+07	1.11E+07	1.19E+07	1.06E+00	9.28E+00	up
10	Salicylamide	1.37E+02	C7H7NO2	[M+H]+	9.00E+00	9.00E+00	9.00E+00	4.79E+05	4.04E+05	4.67E+05	1.06E+00	5.00E+04	up
11	6-Hydroxynicotinic acid	1.39E+02	C6H5NO3	[M+H]+	9.00E+00	9.00E+00	9.00E+00	7.21E+05	1.28E+06	5.04E+05	1.06E+00	9.28E+04	up
12	2-Amino-4,5-dihydro-1H-imidazole-4-acetic acid	1.43E+02	C5H9N3O2	[M+H]+	1.08E+06	1.08E+06	1.18E+06	2.46E+06	3.61E+06	3.45E+06	1.03E+00	2.85E+00	up
13	Stachydrine	1.43E+02	C7H13NO2	[M+H]+	8.07E+06	9.32E+06	8.20E+06	2.68E+07	1.85E+07	2.65E+07	1.03E+00	2.80E+00	up
14	Methyl L-pyroglutamate	1.43E+02	C6H9NO3	[M+H]+	9.00E+00	9.00E+00	9.00E+00	1.56E+07	1.54E+07	1.78E+07	1.06E+00	1.81E+06	up
15	Nornicotine	1.48E+02	C9H12N2	[M+H]+	9.00E+00	9.00E+00	9.00E+00	1.35E+05	1.96E+05	1.52E+05	1.06E+00	1.79E+04	up
16	4-Hydroxymandelonitrile	1.49E+02	C8H7NO2	[M+H]+	1.39E+06	1.27E+06	1.26E+06	2.79E+07	2.70E+07	2.68E+07	1.06E+00	2.08E+01	up
17	2-Acetyl-3-ethylpyrazine	1.50E+02	C8H10N2O	[M+H]+	9.00E+00	9.00E+00	9.00E+00	5.24E+04	6.66E+04	7.74E+04	1.06E+00	7.27E+03	up
18	2(3H)-Benzothiazolone	1.51E+02	C7H5NOS	[M-H]-	9.00E+00	9.00E+00	9.00E+00	2.50E+04	1.88E+04	1.76E+04	1.06E+00	2.28E+03	up
19	3-Hydroxyanthranilic acid	1.53E+02	C7H7NO3	[M+H]+	9.00E+00	9.00E+00	9.00E+00	2.29E+06	1.90E+06	2.17E+06	1.06E+00	2.35E+05	up
20	Vanillylamine	1.53E+02	C8H11NO2	[M+H]+	9.00E+00	9.00E+00	9.00E+00	3.10E+05	2.39E+05	2.58E+05	1.06E+00	2.99E+04	up
21	Indole-2-carboxylic acid	1.61E+02	C9H7NO2	[M-H]-	9.00E+00	9.00E+00	9.00E+00	5.21E+05	4.02E+05	4.32E+05	1.06E+00	5.02E+04	up
22	Tryptophol	1.61E+02	C10H11NO	[M-H]-	9.00E+00	9.00E+00	9.00E+00	7.59E+05	5.79E+05	6.53E+05	1.06E+00	7.38E+04	up

23	4,5,6-Trihydroxy-2-cyclohexen-1-ylideneacetonitrile	1.67E+02	C8H9NO3	[M+H]+	5.91E+05	7.15E+05	7.51E+05	2.81E+06	2.70E+06	2.68E+06	1.06E+00	3.98E+00	up
24	Synephrine; 4-[1-Hydroxy-2-(methylamino)ethyl]phenol	1.67E+02	C9H13NO2	[M+H]+	1.04E+05	1.36E+05	1.25E+05	1.35E+06	1.28E+06	1.28E+06	1.06E+00	1.07E+01	up
25	N-Hydroxytryptamine*	1.76E+02	C10H12N2O	[M+H]+	9.00E+00	9.00E+00	9.00E+00	1.14E+05	1.10E+05	1.08E+05	1.06E+00	1.23E+04	up
26	Serotonin*	1.76E+02	C10H12N2O	[M+H]+	9.00E+00	9.00E+00	9.00E+00	1.34E+05	1.37E+05	1.38E+05	1.06E+00	1.51E+04	up
27	4-hydroxy-5-(2-oxo-l-pyrrolidinyl)-benzoic Acid	1.81E+02	C9H11NO3	[M+H]+	4.26E+06	3.59E+06	4.05E+06	3.96E+07	3.62E+07	3.95E+07	1.06E+00	9.70E+00	up
28	N-(2-Hydroxy-4-methoxyphenyl)acetamide	1.81E+02	C9H11NO3	[M+H]+	1.59E+06	1.17E+06	1.24E+06	1.34E+07	1.19E+07	1.33E+07	1.06E+00	9.70E+00	up
29	3-Indoleacrylic acid	1.87E+02	C11H9NO2	[M+H]+	1.84E+07	1.91E+07	2.26E+07	2.50E+06	2.04E+06	2.41E+06	1.06E+00	1.16E-01	down
30	3-amino-2-naphthoic acid	1.87E+02	C11H9O2N	[M+H]+	1.88E+07	1.95E+07	2.32E+07	2.42E+06	2.17E+06	2.67E+06	1.06E+00	1.18E-01	down
31	N-(4-Aminobutyl)benzamide	1.92E+02	C11H16N2O	[M+H]+	2.19E+05	2.42E+05	2.76E+05	1.23E+06	1.05E+06	1.18E+06	1.06E+00	4.69E+00	up
32	1-Methoxy-indole-3-acetamide	2.04E+02	C11H12N2O2	[M+H]+	3.81E+07	3.32E+07	4.50E+07	4.52E+06	4.00E+06	4.37E+06	1.06E+00	1.11E-01	down
33	2-(Acetylamino)-3-phenyl-2-propenoic acid	2.05E+02	C11H11NO3	[M+H]+	1.11E+05	1.51E+05	1.39E+05	2.95E+05	3.40E+05	3.14E+05	1.03E+00	2.36E+00	up
34	1-Acetyl-β-carboline	2.10E+02	C13H10N2O	[M+H]+	9.00E+00	9.00E+00	9.00E+00	3.64E+05	3.13E+05	3.29E+05	1.06E+00	3.73E+04	up
35	Ailanindole	2.12E+02	C12H8N2O2	[M+H]+	2.97E+04	8.17E+04	5.58E+04	4.73E+06	4.11E+06	3.71E+06	1.05E+00	7.50E+01	up
36	(2E,6E,8E)-N-(2-hydroxy-2-methylpropyl)-10-oxo-2,6,8-decatrienamide	2.19E+02	C14H21NO	[M+H]+	9.00E+00	9.00E+00	9.00E+00	5.94E+04	5.01E+04	4.93E+04	1.06E+00	5.88E+03	up
37	Methyl dioxindole-3-acetate	2.21E+02	C11H11NO4	[M+H]+	6.78E+04	7.24E+04	7.41E+04	2.29E+05	2.04E+05	2.22E+05	1.06E+00	3.06E+00	up
38	Crenatine	2.26E+02	C14H14N2O	[M+H]+	9.00E+00	9.00E+00	9.00E+00	2.17E+05	2.23E+05	2.16E+05	1.06E+00	2.43E+04	up
39	p-Coumaroylputrescine	2.34E+02	C13H18N2O2	[M+H]+	6.97E+04	9.54E+04	5.68E+04	5.02E+05	4.83E+05	5.02E+05	1.05E+00	6.70E+00	up
40	Dihydrocaffeoylputrescine	2.52E+02	C13H20N2O3	[M+H]+	4.84E+05	4.01E+05	3.47E+05	8.47E+05	7.43E+05	9.78E+05	1.01E+00	2.08E+00	up
41	Platydesmine	2.59E+02	C15H17NO3	[M+H]+	8.88E+03	8.70E+02	2.56E+03	7.87E+05	6.59E+05	7.58E+05	1.03E+00	1.79E+02	up
42	3-(2'-Hydroxyl-phenyl)-4-(3H)-quinazolone	2.66E+02	C15H10N2O3	[M+H]+	9.00E+00	9.00E+00	9.00E+00	7.84E+04	9.92E+04	9.52E+04	1.06E+00	1.01E+04	up
43	Caffeoylcholine	2.66E+02	C14H20NO4+	[M]+	2.70E+04	2.71E+04	3.41E+04	9.94E+05	6.05E+05	9.73E+05	1.06E+00	2.92E+01	up
44	Dihydroferuloylputrescine	2.66E+02	C14H22N2O3	[M+H]+	5.13E+06	5.94E+06	6.02E+06	5.06E+07	5.10E+07	5.24E+07	1.06E+00	9.01E+00	up
45	p-Coumaroylagmatine	2.76E+02	C14H20N4O2	[M+H]+	9.00E+00	9.00E+00	9.00E+00	1.70E+06	1.26E+06	1.69E+06	1.06E+00	1.72E+05	up
46	Feruloylcholine	2.80E+02	C15H22NO4+	[M]+	9.00E+00	9.00E+00	9.00E+00	2.62E+05	2.82E+05	2.96E+05	1.06E+00	3.12E+04	up

47	(±)-Zanthoxylumamide E	2.81E+02	C16H27NO3	[M+H]+	3.17E+03	2.12E+03	1.41E+03	3.14E+04	5.58E+04	4.38E+04	1.04E+00	1.96E+01	up
48	N-p-Coumaroylhydroxyagmatine	2.92E+02	C14H20N4O3	[M-H]-	9.00E+00	9.00E+00	9.00E+00	5.20E+05	3.97E+05	4.34E+05	1.06E+00	5.00E+04	up
49	Dihydrobungeanool	2.93E+02	C18H31NO2	[M+H]+	2.13E+04	3.12E+04	1.75E+04	9.00E+00	9.00E+00	9.00E+00	1.06E+00	3.85E-04	down
50	Sinapoylputrescine	2.94E+02	C15H22N2O4	[M+H]+	4.56E+04	2.64E+04	3.19E+04	6.06E+05	6.47E+05	6.58E+05	1.06E+00	1.84E+01	up
51	(E)-3-(3',5'-Dimethoxy-4'-hydroxy-benzylidene')-2 -indolinone	2.97E+02	C17H15NO4	[M+H]+	9.00E+00	9.00E+00	9.00E+00	4.62E+05	4.53E+05	5.13E+05	1.06E+00	5.29E+04	up
52	Dobutamine	3.01E+02	C18H23NO3	[M+H]+	9.00E+00	9.00E+00	9.00E+00	7.64E+04	7.45E+04	5.99E+04	1.06E+00	7.81E+03	up
53	Sinapine	3.10E+02	C16H24NO5+	[M]+	3.41E+04	2.62E+04	2.37E+04	4.62E+05	4.65E+05	5.20E+05	1.06E+00	1.72E+01	up
54	N-Feruloyltyramine	3.13E+02	C18H19NO4	[M+H]+	9.00E+00	9.00E+00	9.00E+00	8.12E+04	8.75E+04	9.02E+04	1.06E+00	9.59E+03	up
55	Feruloylspermidine	3.21E+02	C17H27N3O3	[M+H]+	1.90E+04	2.71E+04	1.65E+04	3.71E+06	2.09E+06	3.68E+06	1.06E+00	1.51E+02	up
56	N-Feruloyl-3-methoxytyramine	3.43E+02	C19H21NO5	[M+H]+	9.00E+00	9.00E+00	9.00E+00	1.40E+06	1.97E+06	2.13E+06	1.06E+00	2.04E+05	up
57	N-Cis-Feruloyl-3'-O-methyldopamine	3.43E+02	C19H21NO5	[M+H]+	9.00E+00	9.00E+00	9.00E+00	2.64E+05	4.12E+05	3.51E+05	1.06E+00	3.80E+04	up
58	N-p-Coumaroylspermine	3.48E+02	C19H32N4O2	[M+H]+	9.00E+00	9.00E+00	9.00E+00	2.64E+05	2.58E+05	2.40E+05	1.06E+00	2.82E+04	up
59	N-Feruloylserotonin	3.52E+02	C20H20N2O4	[M+H]+	9.00E+00	9.00E+00	9.00E+00	1.01E+06	9.52E+05	8.95E+05	1.06E+00	1.06E+05	up
60	Haplopine-glucoside	4.07E+02	C19H21NO9	[M+H]+	1.16E+04	2.88E+04	2.30E+04	1.59E+05	1.62E+05	1.65E+05	1.03E+00	7.67E+00	up
61	Glycohaplopine	4.08E+02	C19H21NO9	[M+H]+	1.88E+04	6.68E+03	2.15E+04	1.50E+05	1.28E+05	1.33E+05	1.01E+00	8.76E+00	up
62	N,N'-Diferuloylputrescine	4.40E+02	C24H28N2O6	[M+H]+	9.00E+00	9.00E+00	9.00E+00	5.07E+05	5.10E+05	4.40E+05	1.06E+00	5.40E+04	up
63	3'-Glucosyl-6,7-dihydroxy-N-methyl-benzyltetrah ydroisoquinoline	4.47E+02	C23H29NO8	[M+H]+	9.00E+00	9.00E+00	9.00E+00	1.34E+05	1.21E+05	1.40E+05	1.06E+00	1.46E+04	up
64	18-Demethylparaensidimerin C	4.68E+02	C29H28N2O4	[M+H]+	7.32E+04	6.44E+04	7.47E+04	2.10E+05	3.33E+05	1.67E+05	1.00E+00	3.34E+00	up
65	N-(4'-O-glycosyl)-feruloyl agmatine	4.68E+02	C21H32N4O8	[M+H]+	4.91E+04	5.54E+04	3.95E+04	5.47E+05	4.46E+05	4.77E+05	1.06E+00	1.02E+01	up
66	2-Glucosyl-glucosyloxy-2-phenylacetic acid amide	4.75E+02	C20H29NO12	[M-H]-	9.00E+00	9.00E+00	9.00E+00	1.50E+05	1.28E+05	1.33E+05	1.06E+00	1.52E+04	up
67	6-O-methyl-N-deacetylisoipecosidic acid	5.23E+02	C25H33NO11	[M+H]+	3.60E+04	3.36E+04	2.41E+04	2.38E+06	2.88E+06	2.66E+06	1.06E+00	8.46E+01	up
**Amino acids and derivatives**													
68	N,N-Dimethylglycine	1.03E+02	C4H9NO2	[M+H]+	1.26E+06	1.60E+06	1.12E+06	4.35E+05	5.26E+05	4.69E+05	1.03E+00	3.59E-01	down
69	N-Ethylmaleimide (NEM)	1.25E+02	C6H7NO2	[M+H]+	9.00E+00	9.00E+00	9.00E+00	2.44E+05	2.30E+05	2.34E+05	1.06E+00	2.63E+04	up

70	N-Propionylglycine	1.31E+02	C5H9NO3	[M-H]-	9.00E+00	9.00E+00	9.00E+00	1.19E+06	7.50E+05	7.47E+05	1.06E+00	9.96E+04	up
71	L-Isoleucine*	1.31E+02	C6H13NO2	[M+H]+	4.33E+05	9.26E+04	1.19E+05	6.35E+06	5.67E+06	5.91E+06	1.03E+00	2.78E+01	up
72	L-Norleucine*	1.31E+02	C6H13NO2	[M+H]+	1.90E+05	1.30E+05	1.43E+05	6.57E+06	6.16E+06	6.15E+06	1.06E+00	4.09E+01	up
73	L-Leucine*	1.31E+02	C6H13NO2	[M+H]+	2.06E+05	1.82E+05	2.03E+05	3.55E+06	6.03E+06	3.62E+06	1.06E+00	2.24E+01	up
74	S-Methyl-L-cysteine	1.35E+02	C4H9NO2S	[M+H]+	9.00E+00	9.00E+00	9.00E+00	7.07E+05	5.30E+05	5.00E+05	1.06E+00	6.44E+04	up
75	L-Tyramine	1.37E+02	C8H11NO	[M+H]+	3.15E+05	3.77E+05	3.71E+05	1.27E+06	1.06E+06	8.78E+05	1.04E+00	3.01E+00	up
76	L-Cyclopentylglycine	1.43E+02	C7H13NO2	[M+H]+	2.14E+07	2.26E+07	2.15E+07	6.13E+07	6.38E+07	5.77E+07	1.06E+00	2.79E+00	up
77	D-Proline betaine	1.44E+02	C7H14NO2+	[M]+	1.06E+06	1.15E+06	1.13E+06	3.69E+06	3.59E+06	3.55E+06	1.06E+00	3.23E+00	up
78	L-Methionine	1.49E+02	C5H11NO2S	[M+H]+	1.28E+06	1.09E+06	1.12E+06	2.26E+07	2.33E+07	2.43E+07	1.06E+00	2.01E+01	up
79	3-Methylcrotonyl-L-glycine	1.57E+02	C7H11NO3	[M+H]+	9.00E+00	9.00E+00	9.00E+00	1.73E+05	2.07E+05	1.73E+05	1.06E+00	2.05E+04	up
80	N-Isovaleroylglycine	1.59E+02	C7H13NO3	[M+H]+	9.00E+00	9.00E+00	9.00E+00	3.36E+05	3.54E+05	3.91E+05	1.06E+00	4.00E+04	up
81	L-Alanyl-L-Alanine	1.60E+02	C6H12N2O3	[M-H]-	4.26E+03	5.72E+03	4.96E+03	8.52E+04	8.33E+04	8.36E+04	1.06E+00	1.69E+01	up
82	S-Allyl-L-cysteine	1.61E+02	C6H11NO2S	[M+H]+	4.12E+04	2.61E+04	2.90E+04	6.35E+06	6.82E+06	6.87E+06	1.06E+00	2.08E+02	up
83	3-Hydroxy-3-methylpentane-1,5-dioic acid	1.62E+02	C6H10O5	[M-H]-	4.51E+04	2.83E+04	3.90E+04	7.28E+06	7.27E+06	8.08E+06	1.06E+00	2.01E+02	up
84	L-Methionine methyl ester	1.63E+02	C6H13NO2S	[M+H]+	5.18E+04	4.76E+04	5.76E+04	8.33E+05	7.87E+05	8.64E+05	1.06E+00	1.58E+01	up
85	L-Phenylalanine	1.65E+02	C9H11NO2	[M+H]+	4.25E+05	4.68E+05	5.22E+05	2.34E+06	2.13E+06	2.24E+06	1.06E+00	4.75E+00	up
86	Cyclo(L-Ala-L-Pro)	1.68E+02	C8H12N2O2	[M+H]+	9.00E+00	9.00E+00	9.00E+00	1.31E+06	1.64E+06	1.57E+06	1.06E+00	1.67E+05	up
87	3-Methyl-L-Histidine	1.69E+02	C7H11N3O2	[M+H]+	9.00E+00	9.00E+00	9.00E+00	1.53E+05	1.72E+05	1.92E+05	1.06E+00	1.91E+04	up
88	Cyclo(Val-Ala)	1.70E+02	C8H14N2O2	[M+H]+	9.00E+00	9.00E+00	9.00E+00	7.96E+05	6.50E+05	6.95E+05	1.06E+00	7.93E+04	up
89	N-α-Acetyl-L-ornithine	1.74E+02	C7H14N2O3	[M-H]-	6.06E+06	6.46E+06	5.61E+06	7.37E+05	6.71E+05	5.96E+05	1.06E+00	1.10E-01	down
90	L-Arginine	1.74E+02	C6H14N4O2	[M+H]+	1.11E+08	1.29E+08	9.62E+07	4.08E+07	3.94E+07	3.51E+07	1.05E+00	3.43E-01	down
91	L-Citrulline	1.75E+02	C6H13N3O3	[M+H]+	1.31E+06	1.36E+06	1.38E+06	1.77E+05	1.94E+05	1.94E+05	1.06E+00	1.39E-01	down
92	L-Dihomomethionine	1.77E+02	C7H15NO2S	[M+H]+	9.00E+00	9.00E+00	9.00E+00	1.50E+05	1.04E+05	9.71E+04	1.06E+00	1.30E+04	up
93	L-Tyrosine	1.81E+02	C9H11NO3	[M+H]+	4.20E+05	3.39E+05	3.67E+05	4.17E+06	3.85E+06	3.76E+06	1.06E+00	1.05E+01	up
94	Cyclo(Ser-Pro)	1.84E+02	C8H12N2O3	[M+H]+	9.00E+00	9.00E+00	9.00E+00	3.36E+06	3.03E+06	2.81E+06	1.06E+00	3.41E+05	up
95	N-Acetyl-L-Glutamine	1.88E+02	C7H12N2O4	[M-H]-	3.13E+05	7.86E+05	1.91E+05	2.19E+07	2.07E+07	2.25E+07	1.04E+00	5.04E+01	up
96	N6-Acetyl-L-lysine	1.88E+02	C8H16N2O3	[M+H]+	4.42E+05	4.22E+05	3.05E+05	1.20E+06	1.30E+06	1.44E+06	1.04E+00	3.37E+00	up

97	Arginine methyl ester*	1.88E+02	C7H16N4O2	[M+H]+	9.00E+00	9.00E+00	9.00E+00	5.06E+05	5.73E+05	6.75E+05	1.06E+00	6.50E+04	up
98	Trimethyllysine	1.88E+02	C9H20N2O2	[M+H]+	7.12E+05	7.84E+05	8.98E+05	6.19E+06	5.41E+06	5.57E+06	1.06E+00	7.17E+00	up
99	N-Acetyl-L-glutamic acid	1.89E+02	C7H11NO5	[M-H]-	2.11E+04	1.67E+04	2.84E+04	2.26E+05	1.58E+05	2.37E+05	1.05E+00	9.37E+00	up
100	N-Phenylacetylglycine	1.93E+02	C10H11NO3	[M+H]+	1.37E+04	1.67E+04	1.87E+04	8.49E+06	7.06E+06	7.29E+06	1.06E+00	4.65E+02	up
101	L-Tyrosine methyl ester	1.95E+02	C10H13NO3	[M+H]+	4.16E+04	3.69E+04	5.04E+04	1.55E+06	1.71E+06	1.80E+06	1.06E+00	3.93E+01	up
102	Cyclo(D-Val-L-Pro)	1.96E+02	C10H16N2O2	[M+H]+	9.00E+00	9.00E+00	9.00E+00	3.80E+06	4.00E+06	3.95E+06	1.06E+00	4.35E+05	up
103	L-Tryptophan	2.04E+02	C11H12N2O2	[M-H]-	5.63E+06	4.91E+06	5.78E+06	1.10E+05	1.21E+05	9.58E+04	1.06E+00	2.00E-02	down
104	N-Acetyl-L-phenylalanine	2.07E+02	C11H13NO3	[M-H]-	1.49E+05	1.26E+05	2.14E+05	7.45E+06	6.56E+06	6.40E+06	1.06E+00	4.17E+01	up
105	N-Acetyl-L-Arginine	2.16E+02	C8H16N4O3	[M+H]+	9.00E+00	9.00E+00	9.00E+00	8.21E+05	8.45E+05	8.63E+05	1.06E+00	9.37E+04	up
106	5-Hydroxy-L-tryptophan	2.20E+02	C11H12N2O3	[M+H]+	9.00E+00	9.00E+00	9.00E+00	1.90E+05	1.76E+05	8.99E+04	1.06E+00	1.69E+04	up
107	L-Glycyl-L-phenylalanine	2.22E+02	C11H14N2O3	[M+H]+	8.32E+03	3.86E+03	9.24E+03	4.11E+06	3.78E+06	4.37E+06	1.06E+00	5.73E+02	up
108	N-Acetyl-L-tyrosine	2.23E+02	C11H13NO4	[M+H]+	3.25E+04	2.72E+04	1.52E+04	8.07E+05	9.49E+05	9.45E+05	1.05E+00	3.60E+01	up
109	Cyclo(L-Leu-trans-4-hydroxy-L-Pro)	2.26E+02	C11H18N2O3	[M+H]+	9.00E+00	9.00E+00	9.00E+00	7.68E+05	5.82E+05	6.64E+05	1.06E+00	7.46E+04	up
110	L-Prolyl-L-Leucine	2.29E+02	C11H20N2O3	[M+H]+	9.00E+00	9.00E+00	9.00E+00	1.66E+07	1.39E+07	1.54E+07	1.06E+00	1.70E+06	up
111	L-Valyl-L-Leucine	2.31E+02	C11H22N2O3	[M+H]+	1.74E+03	8.20E+03	7.40E+02	2.40E+06	2.14E+06	2.34E+06	1.04E+00	6.45E+02	up
112	Cyclo(Tyr-Ala)	2.34E+02	C12H14N2O3	[M+H]+	9.00E+00	9.00E+00	9.00E+00	1.67E+06	1.77E+06	1.69E+06	1.06E+00	1.90E+05	up
113	L-Isoleucyl-L-Aspartate	2.46E+02	C10H18N2O5	[M+H]+	2.74E+04	3.11E+04	4.64E+04	6.30E+06	6.10E+06	6.10E+06	1.06E+00	1.76E+02	up
114	γ-Glutamyl-L-valine	2.46E+02	C10H18N2O5	[M+H]+	1.34E+04	3.70E+04	4.22E+04	4.09E+05	4.43E+05	5.16E+05	1.03E+00	1.48E+01	up
115	Phenylacetyl-L-glutamine	2.64E+02	C13H16N2O4	[M-H]-	9.00E+00	9.00E+00	9.00E+00	5.46E+04	3.82E+04	5.21E+04	1.06E+00	5.37E+03	up
116	L-Valyl-L-Phenylalanine	2.64E+02	C14H20N2O3	[M+H]+	9.00E+00	9.00E+00	9.00E+00	7.10E+06	4.40E+06	6.14E+06	1.06E+00	6.54E+05	up
117	L-Saccharopine	2.76E+02	C11H20N2O6	[M-H]-	8.90E+03	8.29E+03	9.45E+03	2.50E+05	2.15E+05	1.96E+05	1.06E+00	2.48E+01	up
118	γ-glutamylmethionine	2.78E+02	C10H18N2O5S	[M+H]+	9.00E+00	9.00E+00	9.00E+00	1.46E+05	1.38E+05	1.51E+05	1.06E+00	1.61E+04	up
119	L-Leucyl-L-phenylalanine	2.78E+02	C15H22N2O3	[M+H]+	6.63E+02	3.55E+03	5.63E+02	3.06E+06	2.23E+06	2.61E+06	1.05E+00	1.65E+03	up
120	L-Aspartyl-L-Phenylalanine	2.80E+02	C13H16N2O5	[M+H]+	4.63E+04	5.27E+04	8.53E+04	1.37E+07	1.05E+07	1.38E+07	1.06E+00	2.06E+02	up
121	N-Benzoyl-(2R,3S)-3-phenylisoserine	2.85E+02	C16H15NO4	[M+H]+	9.00E+00	9.00E+00	9.00E+00	4.33E+05	3.66E+05	4.45E+05	1.06E+00	4.61E+04	up
122	γ-Glutamylphenylalanine	2.94E+02	C14H18N2O5	[M-H]-	5.87E+04	6.36E+04	5.52E+04	1.75E+05	1.99E+05	1.54E+05	1.05E+00	2.97E+00	up
123	γ-Glutamyltyrosine	3.10E+02	C14H18N2O6	[M+H]+	6.17E+04	5.99E+04	8.00E+04	3.45E+05	4.16E+05	2.94E+05	1.05E+00	5.24E+00	up

124	L-Phenylalanyl-L-phenylalanine	3.12E+02	C18H20N2O3	[M+H]+	3.53E+04	1.35E+04	3.40E+04	2.19E+07	1.44E+07	1.74E+07	1.06E+00	6.48E+02	up
125	S-(5'-Adenosyl)-L-methionine	3.98E+02	C15H22N6O5S	[M+H]+	5.50E+05	5.97E+05	6.30E+05	9.00E+00	9.00E+00	9.00E+00	1.06E+00	1.52E-05	down
126	L-Aspartic acid-O-diglucoside	4.57E+02	C16H27NO14	[M+H]+	2.36E+07	2.48E+07	2.42E+07	4.26E+05	4.12E+05	3.03E+05	1.06E+00	1.57E-02	down
**Flavonoids**													
127	7-Hydroxyflavone	2.38E+02	C15H10O3	[M+H]+	9.00E+00	9.00E+00	9.00E+00	1.43E+05	1.50E+05	1.45E+05	1.06E+00	1.62E+04	up
128	3,4'-Dihydroxyflavone	2.54E+02	C15H10O4	[M+H]+	9.00E+00	9.00E+00	9.00E+00	2.95E+04	7.01E+04	2.87E+04	1.06E+00	4.75E+03	up
129	Pinocembrin (Dihydrochrysin)	2.56E+02	C15H12O4	[M+H]+	9.00E+00	9.00E+00	9.00E+00	2.58E+07	2.38E+07	2.51E+07	1.06E+00	2.76E+06	up
130	Formononetin (7-Hydroxy-4'-methoxyisoflavone)	2.68E+02	C16H12O4	[M-H]-	9.00E+00	9.00E+00	9.00E+00	4.94E+04	4.21E+04	4.39E+04	1.06E+00	5.02E+03	up
131	6-Hydroxy-2'-methoxyflavone	2.68E+02	C16H12O4	[M-H]-	9.00E+00	9.00E+00	9.00E+00	4.90E+04	4.10E+04	4.71E+04	1.06E+00	5.08E+03	up
132	3',4',7-Trihydroxyflavone	2.70E+02	C15H10O5	[M+H]+	9.00E+00	9.00E+00	9.00E+00	3.38E+05	3.75E+05	3.48E+05	1.06E+00	3.93E+04	up
133	Apigenin	2.70E+02	C15H10O5	[M+H]+	9.00E+00	9.00E+00	9.00E+00	4.97E+06	5.79E+06	5.59E+06	1.06E+00	6.05E+05	up
134	Naringenin chalcone	2.72E+02	C15H12O5	[M+H]+	9.00E+00	9.00E+00	9.00E+00	1.74E+06	1.72E+06	1.77E+06	1.06E+00	1.93E+05	up
135	Butin	2.72E+02	C15H12O5	[M+H]+	9.00E+00	9.00E+00	9.00E+00	1.74E+06	1.72E+06	1.77E+06	1.06E+00	1.93E+05	up
136	Naringenin (5,7,4'-Trihydroxyflavanone)	2.72E+02	C15H12O5	[M+H]+	9.00E+00	9.00E+00	9.00E+00	1.55E+06	1.63E+06	1.64E+06	1.06E+00	1.78E+05	up
137	Phloretin	2.74E+02	C15H14O5	[M-H]-	9.00E+00	9.00E+00	9.00E+00	6.91E+05	7.15E+05	7.30E+05	1.06E+00	7.91E+04	up
138	Genkwanin (Apigenin 7-methyl ether)	2.84E+02	C16H12O5	[M+H]+	9.00E+00	9.00E+00	9.00E+00	6.52E+04	7.68E+04	6.55E+04	1.06E+00	7.68E+03	up
139	Wogonin (5,7-Dihydroxy-8-Methoxyflavone)	2.84E+02	C16H12O5	[M+H]+	9.00E+00	9.00E+00	9.00E+00	4.50E+04	5.22E+04	4.77E+04	1.06E+00	5.37E+03	up
140	Isoscutellarein	2.86E+02	C15H10O6	[M+H]+	9.00E+00	9.00E+00	9.00E+00	5.16E+06	5.75E+06	6.08E+06	1.06E+00	6.29E+05	up
141	Luteolin (5,7,3',4'-Tetrahydroxyflavone)	2.86E+02	C15H10O6	[M+H]+	9.00E+00	9.00E+00	9.00E+00	4.57E+06	4.58E+06	4.68E+06	1.06E+00	5.13E+05	up
142	Kaempferol (3,5,7,4'-Tetrahydroxyflavone)	2.86E+02	C15H10O6	[M-H]-	9.00E+00	9.00E+00	9.00E+00	1.48E+06	1.35E+06	1.43E+06	1.06E+00	1.58E+05	up
143	3,5,7,2'-Tetrahydroxyflavone; Datiscetin	2.86E+02	C15H10O6	[M-H]-	9.00E+00	9.00E+00	9.00E+00	1.79E+06	1.78E+06	1.38E+06	1.06E+00	1.83E+05	up
144	7-O-Methylnaringenin	2.86E+02	C16H14O5	[M+H]+	9.00E+00	9.00E+00	9.00E+00	1.61E+05	1.13E+05	1.52E+05	1.06E+00	1.58E+04	up
145	Sakuranetin	2.86E+02	C16H14O5	[M+H]+	9.00E+00	9.00E+00	9.00E+00	1.21E+05	1.05E+05	1.36E+05	1.06E+00	1.34E+04	up
146	Isosakuranetin (5,7-Dihydroxy-4'-methoxyflavanone)	2.86E+02	C16H14O5	[M+H]+	9.00E+00	9.00E+00	9.00E+00	1.53E+07	1.51E+07	1.65E+07	1.06E+00	1.73E+06	up
147	Aromadendrin (Dihydrokaempferol)	2.88E+02	C15H12O6	[M+H]+	9.00E+00	9.00E+00	9.00E+00	4.12E+06	4.49E+06	4.24E+06	1.06E+00	4.76E+05	up
148	2-Hydroxy-2,3-dihydrogenistein	2.88E+02	C15H12O6	[M-H]-	8.98E+04	4.54E+04	3.89E+04	9.46E+06	1.08E+07	1.07E+07	1.06E+00	1.78E+02	up

149	2-hydroxynaringenin	2.88E+02	C15H12O6	[M-H]-	9.00E+04	4.63E+04	2.66E+04	9.94E+06	1.11E+07	1.03E+07	1.05E+00	1.92E+02	up
150	Eriodictyol (5,7,3',4'-Tetrahydroxyflavanone)	2.88E+02	C15H12O6	[M+H]+	1.27E+04	1.41E+03	3.51E+03	1.38E+06	1.49E+06	1.26E+06	1.04E+00	2.34E+02	up
151	3,4,2',4',6'-Pentahydroxychalcone	2.88E+02	C15H12O6	[M-H]-	8.23E+03	4.83E+03	1.64E+03	1.98E+06	2.42E+06	2.06E+06	1.05E+00	4.40E+02	up
152	Hispidulin (5,7,4'-Trihydroxy-6-methoxyflavone)	3.00E+02	C16H12O6	[M+H]+	9.00E+00	9.00E+00	9.00E+00	2.30E+07	2.59E+07	2.50E+07	1.06E+00	2.74E+06	up
153	Chrysoeriol	3.00E+02	C16H12O6	[M+H]+	9.00E+00	9.00E+00	9.00E+00	2.48E+05	3.46E+05	3.17E+05	1.06E+00	3.38E+04	up
154	Rhamnocitrin (7-Methylkaempferol)	3.00E+02	C16H12O6	[M+H]+	1.52E+04	1.20E+03	4.14E+03	2.23E+07	2.49E+07	2.45E+07	1.05E+00	3.49E+03	up
155	Diosmetin (5,7,3'-Trihydroxy-4'-methoxyflavone)	3.00E+02	C16H12O6	[M+H]+	9.00E+00	9.00E+00	9.00E+00	2.29E+07	2.47E+07	2.54E+07	1.06E+00	2.70E+06	up
156	Gnetifolin B	3.00E+02	C16H12O6	[M-H]-	2.99E+04	8.16E+03	8.16E+03	1.37E+07	1.44E+07	1.39E+07	1.05E+00	9.07E+02	up
157	6,7,8-Tetrahydroxy-5-methoxyflavone	3.00E+02	C16H12O6	[M+H]+	1.85E+04	5.08E+03	3.72E+03	2.30E+07	2.38E+07	2.55E+07	1.05E+00	2.65E+03	up
158	5,7,2'-Trhiyroxy-8-methoxyflavone	3.00E+02	C16H12O6	[M-H]-	3.95E+04	7.98E+03	1.13E+04	1.44E+07	1.52E+07	1.43E+07	1.05E+00	7.47E+02	up
159	4'-Hydroxy-5,7-dimethoxyflavanone	3.00E+02	C17H16O5	[M-H]-	2.51E+06	3.12E+06	2.26E+06	9.54E+05	8.46E+05	8.07E+05	1.04E+00	3.30E-01	down
160	6-HydroxyLuteolin	3.02E+02	C15H10O7	[M-H]-	2.33E+04	1.37E+04	9.07E+03	2.24E+05	2.00E+05	2.16E+05	1.04E+00	1.39E+01	up
161	Isoetin (5,7,2',4',5'-Pentahydroxyflavone)	3.02E+02	C15H10O7	[M-H]-	9.00E+00	9.00E+00	9.00E+00	3.59E+04	1.61E+04	2.89E+04	1.06E+00	3.00E+03	up
162	Quercetin	3.02E+02	C15H10O7	[M+H]+	1.20E+04	6.73E+03	2.82E+03	4.05E+05	3.74E+05	4.22E+05	1.04E+00	5.57E+01	up
163	Homoeriodictyol	3.02E+02	C16H14O6	[M+H]+	9.00E+00	9.00E+00	9.00E+00	1.11E+06	1.19E+06	1.08E+06	1.06E+00	1.25E+05	up
164	Taxifolin(Dihydroquercetin)	3.04E+02	C15H12O7	[M+H]+	5.13E+03	3.43E+03	4.61E+03	1.46E+06	1.45E+06	1.49E+06	1.06E+00	3.34E+02	up
165	5,7,3',4',5'-Pentahydroxydihydroflavone	3.04E+02	C15H12O7	[M-H]-	9.00E+00	9.00E+00	9.00E+00	4.66E+06	3.80E+06	4.29E+06	1.06E+00	4.72E+05	up
166	Epigallocatechin	3.06E+02	C15H14O7	[M-H]-	9.00E+00	9.00E+00	9.00E+00	1.93E+05	2.42E+05	1.77E+05	1.06E+00	2.27E+04	up
167	Isobavachalcone D	3.12E+02	C19H20O4	[M+H]+	3.26E+04	2.88E+04	2.40E+04	1.43E+07	1.34E+07	1.37E+07	1.06E+00	4.84E+02	up
168	3’,4’-Dihydroxy-7,5’-dimethoxyflavone	3.14E+02	C17H14O6	[M+H]+	3.24E+03	1.28E+03	3.22E+03	3.53E+04	5.80E+04	4.95E+04	1.04E+00	1.85E+01	up
169	Isorhamnetin*	3.16E+02	C16H12O7	[M-H]-	6.16E+05	3.28E+05	2.92E+05	1.63E+07	1.49E+07	1.56E+07	1.05E+00	3.78E+01	up
170	Rhamnetin*	3.16E+02	C16H12O7	[M-H]-	1.02E+04	6.75E+03	6.53E+03	5.21E+05	4.80E+05	4.86E+05	1.06E+00	6.34E+01	up
171	Azaleatin (5-O-Methylquercetin)	3.16E+02	C16H12O7	[M+H]+	2.17E+04	2.01E+04	1.75E+04	2.94E+05	3.90E+05	1.32E+05	1.03E+00	1.38E+01	up
172	Tamarixetin (3,3',5,7-Tetrahydroxy-4'-methoxyflavone)	3.16E+02	C16H12O7	[M+H]+	1.49E+05	5.01E+04	3.53E+04	4.86E+06	4.82E+06	4.65E+06	1.04E+00	6.12E+01	up
173	Persicogenin (5,3'-dihydroxy-7,4'-dimethoxyflavanone)	3.16E+02	C17H16O6	[M+H]+	9.00E+00	9.00E+00	9.00E+00	1.20E+06	1.21E+06	1.13E+06	1.06E+00	1.31E+05	up

174	4'-hydroxy-2,4,6-trimethoxydihydrochalcone	3.16E+02	C18H20O5	[M-H]-	9.00E+00	9.00E+00	9.00E+00	1.00E+06	1.03E+06	1.04E+06	1.06E+00	1.14E+05	up
175	Salvigenin	3.28E+02	C18H16O6	[M+H]+	3.01E+04	2.43E+04	3.26E+04	2.02E+05	2.12E+05	2.19E+05	1.06E+00	7.27E+00	up
176	3-Hydroxy-5,7,8-trimethoxyflavone	3.28E+02	C18H16O6	[M+H]+	9.00E+00	9.00E+00	9.00E+00	2.37E+05	2.50E+05	2.91E+05	1.06E+00	2.88E+04	up
177	Rehderianin I	3.30E+02	C17H14O7	[M+H]+	9.00E+00	9.00E+00	9.00E+00	9.05E+05	1.03E+06	9.77E+05	1.06E+00	1.08E+05	up
178	3,7-Di-O-methylquercetin	3.30E+02	C17H14O7	[M-H]-	9.00E+00	9.00E+00	9.00E+00	4.90E+05	5.29E+05	4.91E+05	1.06E+00	5.59E+04	up
179	4',5,7-Trihydroxy-3',6-dimethoxyflavone (Jaceosidin)	3.30E+02	C17H14O7	[M+H]+	9.00E+00	9.00E+00	9.00E+00	6.84E+05	7.05E+05	6.97E+05	1.06E+00	7.73E+04	up
180	Mearnsetin	3.32E+02	C16H12O8	[M+H]+	5.80E+04	3.25E+04	2.59E+04	7.52E+05	7.94E+05	8.62E+05	1.05E+00	2.07E+01	up
181	5,7,8,4'-Tetramethoxyflavone*	3.42E+02	C19H18O6	[M+H]+	1.45E+05	1.55E+05	1.71E+05	2.89E+06	3.08E+06	3.33E+06	1.06E+00	1.98E+01	up
182	Nevadensin	3.44E+02	C18H16O7	[M+H]+	6.88E+02	1.02E+03	3.24E+03	3.03E+04	2.16E+04	3.27E+04	1.01E+00	1.71E+01	up
183	Eupatilin (5,7-Dihydroxy-3',4',6-Trimethoxyflavone)	3.44E+02	C18H16O7	[M+H]+	9.00E+00	9.00E+00	9.00E+00	2.45E+05	2.05E+05	2.44E+05	1.06E+00	2.57E+04	up
184	Eupatorin	3.44E+02	C18H16O7	[M+H]+	3.04E+03	6.88E+02	3.07E+03	1.85E+05	1.95E+05	1.85E+05	1.04E+00	8.31E+01	up
185	5,7-Dihydroxy-3',4',5'-trimethoxyflavone	3.44E+02	C18H16O7	[M+H]+	9.00E+00	9.00E+00	9.00E+00	3.25E+05	3.52E+05	3.64E+05	1.06E+00	3.86E+04	up
186	5,6,7,4'-Tetramethoxyflavanone	3.44E+02	C19H20O6	[M+H]+	9.00E+00	9.00E+00	9.00E+00	5.59E+04	6.37E+04	5.27E+04	1.06E+00	6.38E+03	up
187	Limocitrin (5,7,4'-trihydroxy-8,3'-dimethoxyflavonol)*	3.46E+02	C17H14O8	[M+H]+	2.82E+04	1.51E+04	1.42E+04	1.67E+06	1.83E+06	2.03E+06	1.06E+00	9.60E+01	up
188	5-Hydroxy-3,7,3',4'-tetramethoxyflavone (Retusin)	3.58E+02	C19H18O7	[M+H]+	3.86E+04	3.12E+04	3.71E+04	1.19E+05	1.40E+05	1.39E+05	1.05E+00	3.72E+00	up
189	Acerosin	3.60E+02	C18H16O8	[M-H]-	9.00E+00	9.00E+00	9.00E+00	2.57E+04	3.87E+04	2.96E+04	1.06E+00	3.48E+03	up
190	3,4',5,6,7-Pentamethoxyflavone*	3.72E+02	C20H20O7	[M+H]+	5.34E+03	1.34E+04	8.79E+03	1.27E+05	1.61E+05	1.48E+05	1.04E+00	1.59E+01	up
191	Tangeretin (4',5,6,7,8-Pentamethoxyflavone)*	3.72E+02	C20H20O7	[M+H]+	1.17E+06	9.97E+05	1.16E+06	2.87E+07	2.90E+07	3.14E+07	1.06E+00	2.68E+01	up
192	3',4',5',5,7-Pentamethoxyflavone*	3.72E+02	C20H20O7	[M+H]+	8.17E+04	8.76E+04	1.02E+05	2.59E+06	2.67E+06	2.95E+06	1.06E+00	3.02E+01	up
193	Sinensetin (5,6,7,3',4'-pentamethoxyflavone)*	3.72E+02	C20H20O7	[M+H]+	1.03E+04	1.41E+04	9.91E+03	2.22E+05	2.31E+05	2.48E+05	1.06E+00	2.04E+01	up
194	Isosinensetin	3.72E+02	C20H20O7	[M+H]+	2.19E+05	2.07E+05	2.60E+05	5.40E+06	6.65E+06	6.41E+06	1.06E+00	2.69E+01	up
195	5,4′-Dihydroxy-3,6,7,3′-tetramethoxyflavone	3.74E+02	C19H18O8	[M+H]+	1.85E+04	1.69E+04	1.21E+04	8.29E+05	8.40E+05	9.19E+05	1.06E+00	5.44E+01	up
196	Casticin (5,3'-Dihydroxy-3,6,7,4'-Tetramethoxyflavone)	3.74E+02	C19H18O8	[M+H]+	4.50E+03	5.01E+03	1.38E+03	1.97E+05	1.87E+05	1.96E+05	1.04E+00	5.33E+01	up

197	Chrysosplenetin	3.74E+02	C19H18O8	[M+H]+	3.25E+03	2.85E+03	4.11E+03	1.88E+05	1.86E+05	2.13E+05	1.06E+00	5.76E+01	up
198	Skullcapflavone II	3.74E+02	C19H18O8	[M+H]+	6.11E+03	9.35E+03	1.24E+04	3.81E+05	3.64E+05	4.15E+05	1.06E+00	4.17E+01	up
199	5,7,3',4',5'-Pentamethoxyflavanone	3.74E+02	C20H22O7	[M+H]+	9.00E+00	9.00E+00	9.00E+00	2.01E+04	2.12E+04	2.06E+04	1.06E+00	2.29E+03	up
200	5-Hydroxyauranetin	3.88E+02	C20H20O8	[M+H]+	2.46E+04	2.55E+04	3.17E+04	4.69E+05	4.92E+05	5.28E+05	1.06E+00	1.82E+01	up
201	5-Demethylnobiletin*	3.88E+02	C20H20O8	[M+H]+	1.82E+05	1.57E+05	2.07E+05	3.17E+06	3.41E+06	3.38E+06	1.06E+00	1.82E+01	up
202	3'-Demethylnobiletin*	3.88E+02	C20H20O8	[M+H]+	3.21E+04	4.30E+04	3.51E+04	3.20E+06	3.48E+06	3.76E+06	1.06E+00	9.47E+01	up
203	Gardenin E	3.90E+02	C19H18O9	[M+H]+	9.00E+00	9.00E+00	9.00E+00	1.63E+05	1.68E+05	1.78E+05	1.06E+00	1.89E+04	up
204	Nobiletin (5,6,7,8,3',4'-Hexamethoxyflavone)	4.02E+02	C21H22O8	[M+H]+	1.77E+05	1.96E+05	2.40E+05	6.80E+06	7.00E+06	7.38E+06	1.06E+00	3.45E+01	up
205	Gardenin C	4.04E+02	C20H20O9	[M+H]+	1.51E+05	1.27E+05	1.50E+05	4.56E+06	4.98E+06	5.34E+06	1.06E+00	3.48E+01	up
206	Monohydroxy-hexamethoxyflavone	4.18E+02	C21H22O9	[M+H]+	9.23E+04	1.06E+05	1.13E+05	2.21E+06	2.29E+06	2.36E+06	1.06E+00	2.20E+01	up
207	Gardenin A	4.18E+02	C21H22O9	[M+H]+	1.51E+05	1.40E+05	1.32E+05	4.41E+06	4.87E+06	5.08E+06	1.06E+00	3.39E+01	up
208	Natsudaidain (3-Hydroxy-3',4',5,6,7,8-hexamethoxyflavone)	4.18E+02	C21H22O9	[M-H]-	2.53E+03	2.75E+03	2.14E+03	9.99E+04	1.06E+05	9.26E+04	1.06E+00	4.02E+01	up
209	Liquiritigenin-4'-O-Glucoside (Liquiritin)	4.18E+02	C21H22O9	[M+H]+	9.00E+00	9.00E+00	9.00E+00	5.23E+05	5.72E+05	5.32E+05	1.06E+00	6.03E+04	up
210	Isovitexin	4.32E+02	C21H20O10	[M+H]+	3.17E+05	4.07E+05	3.01E+05	1.02E+07	1.09E+07	1.07E+07	1.06E+00	3.10E+01	up
211	Apigenin-6-C-glucoside (Isovitexin)*	4.32E+02	C21H20O10	[M-H]-	3.07E+05	3.17E+05	2.41E+05	2.47E+06	2.42E+06	2.10E+06	1.06E+00	8.06E+00	up
212	vitexin*	4.32E+02	C21H20O10	[M+H]+	4.17E+05	4.80E+05	4.99E+05	9.04E+06	9.56E+06	9.09E+06	1.06E+00	1.98E+01	up
213	Genistein-8-C-glucoside	4.32E+02	C21H20O10	[M+H]+	3.17E+05	4.07E+05	3.01E+05	1.02E+07	1.09E+07	1.07E+07	1.06E+00	3.10E+01	up
214	3,5,6,7,8,3',4'-Heptamethoxyflavone	4.32E+02	C22H24O9	[M+H]+	1.11E+05	1.14E+05	1.06E+05	3.67E+06	3.92E+06	4.01E+06	1.06E+00	3.50E+01	up
215	Naringenin-7-O-glucoside (Prunin)	4.34E+02	C21H22O10	[M-H]-	3.56E+04	5.05E+04	3.97E+04	5.77E+06	6.30E+06	6.25E+06	1.06E+00	1.46E+02	up
216	Butin-7-O-glucoside	4.34E+02	C21H22O10	[M-H]-	4.36E+04	4.65E+04	3.94E+04	5.40E+06	5.92E+06	5.53E+06	1.06E+00	1.30E+02	up
217	Naringenin-6-C-Glucoside	4.34E+02	C21H22O10	[M+H]+	4.03E+04	5.00E+04	6.51E+04	3.28E+05	4.18E+05	3.83E+05	1.05E+00	7.26E+00	up
218	Isosalipurposide (Phlorizin Chalcone)	4.34E+02	C21H22O10	[M+H]+	2.05E+05	1.68E+05	1.96E+05	4.26E+06	4.35E+06	4.26E+06	1.06E+00	2.26E+01	up
219	Dihydrocharcone-4'-O-glucoside	4.36E+02	C21H24O10	[M-H]-	4.05E+04	6.01E+04	6.01E+04	2.78E+06	2.83E+06	2.57E+06	1.06E+00	5.09E+01	up
220	Phloretin-4'-O-glucoside (Trilobatin)	4.36E+02	C21H24O10	[M-H]-	4.99E+04	6.32E+04	5.40E+04	2.72E+05	3.22E+05	2.71E+05	1.06E+00	5.18E+00	up
221	Catechin gallate	4.42E+02	C22H18O10	[M-H]-	1.76E+04	2.90E+04	3.83E+04	1.02E+05	9.26E+04	1.07E+05	1.00E+00	3.56E+00	up
222	Acacetin-7-O-galactoside	4.46E+02	C22H22O10	[M+H]+	9.00E+00	9.00E+00	9.00E+00	9.55E+05	1.43E+06	1.18E+06	1.06E+00	1.32E+05	up

223	Isorhamnetin-3-O-arabinoside	4.48E+02	C21H20O11	[M+H]+	9.00E+00	9.00E+00	9.00E+00	1.02E+05	9.09E+04	1.12E+05	1.06E+00	1.13E+04	up
224	Luteolin-7-O-glucoside (Cynaroside)	4.48E+02	C21H20O11	[M+H]+	9.00E+00	9.00E+00	9.00E+00	1.17E+06	1.25E+06	1.30E+06	1.06E+00	1.38E+05	up
225	Kaempferol-3-O-glucoside (Astragalin)*	4.48E+02	C21H20O11	[M+H]+	9.00E+00	9.00E+00	9.00E+00	1.13E+06	1.24E+06	8.23E+05	1.06E+00	1.18E+05	up
226	Quercetin-3-O-rhamnoside(Quercitrin)	4.48E+02	C21H20O11	[M+H]+	4.61E+06	4.09E+06	3.74E+06	2.50E+05	2.86E+05	2.83E+05	1.06E+00	6.58E-02	down
227	Kaempferol-7-O-glucoside*	4.48E+02	C21H20O11	[M-H]-	2.69E+04	2.68E+04	7.91E+03	2.14E+05	2.31E+05	2.14E+05	1.01E+00	1.07E+01	up
228	Luteolin-8-C-glucoside (orientin)	4.48E+02	C21H20O11	[M+H]+	2.29E+05	2.06E+05	1.94E+05	1.28E+06	1.96E+06	1.58E+06	1.05E+00	7.66E+00	up
229	Luteolin-6-C-glucoside (Isoorientin)	4.48E+02	C21H20O11	[M-H]-	1.28E+05	1.93E+05	1.20E+05	9.42E+05	9.90E+05	1.03E+06	1.05E+00	6.70E+00	up
230	Sakuranin	4.48E+02	C22H24O10	[M-H]-	9.00E+00	9.00E+00	9.00E+00	9.55E+05	1.43E+06	1.18E+06	1.06E+00	1.32E+05	up
231	Isosakuranin	4.48E+02	C22H24O10	[M+H]+	1.92E+04	3.22E+04	3.43E+04	1.64E+07	1.77E+07	1.73E+07	1.06E+00	6.00E+02	up
232	eriodictyol 7-O-β-D-glucopyranoside	4.50E+02	C21H22O11	[M+H]+	4.33E+06	3.74E+06	3.63E+06	1.61E+07	2.22E+07	2.06E+07	1.05E+00	5.03E+00	up
233	Dihydrokaempferol-3-O-glucoside*	4.50E+02	C21H22O11	[M+H]+	4.76E+06	3.10E+06	3.65E+06	1.58E+07	2.15E+07	2.13E+07	1.04E+00	5.09E+00	up
234	Taxifolin-3-O-rhamnoside (Astilbin)	4.50E+02	C21H22O11	[M-H]-	1.53E+05	1.21E+05	1.21E+05	1.08E+07	1.10E+07	1.01E+07	1.06E+00	8.09E+01	up
235	Dihydrokaempferol-7-O-glucoside*	4.50E+02	C21H22O11	[M-H]-	3.92E+04	5.27E+04	3.49E+04	1.44E+06	1.47E+06	1.30E+06	1.06E+00	3.32E+01	up
236	3',5,5',7-Tetrahydroxyflavanone-7-O-glucoside	4.50E+02	C21H22O11	[M+H]+	1.18E+07	1.15E+07	6.53E+06	7.40E+05	7.63E+05	8.39E+05	1.05E+00	7.85E-02	down
237	6-C-Glucosyl-2-Hydroxynaringenin	4.50E+02	C21H22O11	[M-H]-	2.33E+05	2.83E+05	2.41E+05	4.82E+05	5.45E+05	5.03E+05	1.04E+00	2.02E+00	up
238	Sieboldin	4.52E+02	C21H24O11	[M-H]-	1.47E+04	1.83E+04	1.78E+04	5.00E+05	5.69E+05	4.09E+05	1.06E+00	2.91E+01	up
239	Diosmetin-7-O-galactoside	4.62E+02	C22H22O11	[M+H]+	7.83E+04	6.34E+04	5.80E+04	6.55E+05	8.04E+05	6.32E+05	1.06E+00	1.05E+01	up
240	Chrysoeriol-7-O-glucoside	4.62E+02	C22H22O11	[M-H]-	9.00E+00	9.00E+00	9.00E+00	1.51E+05	1.91E+05	1.19E+05	1.06E+00	1.71E+04	up
241	Hispidulin-8-C-glucoside	4.62E+02	C22H22O11	[M+H]+	9.08E+05	9.66E+05	8.90E+05	8.92E+06	1.05E+07	1.03E+07	1.06E+00	1.07E+01	up
242	6-C-MethylKaempferol-3-glucoside	4.62E+02	C22H22O11	[M+H]+	7.20E+04	3.77E+04	4.04E+04	6.64E+05	5.80E+05	7.07E+05	1.05E+00	1.30E+01	up
243	6-C-Methylquercetin-3-O-rhamnoside	4.62E+02	C22H22O11	[M+H]+	9.00E+00	9.00E+00	9.00E+00	3.55E+05	3.35E+05	3.52E+05	1.06E+00	3.86E+04	up
244	Hispidulin-7-O-Glucoside	4.62E+02	C22H22O11	[M+H]+	5.25E+04	2.60E+04	3.14E+04	5.57E+05	6.45E+05	4.44E+05	1.05E+00	1.50E+01	up
245	Yuanhuanin	4.62E+02	C22H22O11	[M+H]+	6.21E+04	3.92E+04	7.01E+04	3.48E+05	4.77E+05	5.16E+05	1.04E+00	7.83E+00	up
246	Isorhamnetin-3-O-rhamnoside	4.62E+02	C22H22O11	[M+H]+	3.99E+04	4.26E+04	3.57E+04	6.75E+05	7.05E+05	6.44E+05	1.06E+00	1.71E+01	up
247	Diosmetin-6-C-glucoside	4.62E+02	C22H22O11	[M+H]+	7.06E+05	6.81E+05	6.18E+05	6.40E+06	5.76E+06	6.37E+06	1.06E+00	9.25E+00	up
248	Chrysoeriol-8-C-glucoside (scoparin)	4.62E+02	C22H22O11	[M+H]+	9.73E+05	1.12E+06	8.59E+05	7.70E+06	9.36E+06	9.12E+06	1.06E+00	8.87E+00	up
249	Quercetin-3-O-glucoside (Isoquercitrin)*	4.64E+02	C21H20O12	[M-H]-	5.85E+04	4.84E+04	5.80E+04	3.27E+05	2.87E+05	3.30E+05	1.06E+00	5.72E+00	up

250	Quercetin-5-O-β-D-glucoside*	4.64E+02	C21H20O12	[M+H]+	4.30E+05	7.46E+05	5.02E+05	8.04E+06	6.86E+06	9.31E+06	1.05E+00	1.44E+01	up
251	Isohyperoside*	4.64E+02	C21H20O12	[M+H]+	4.08E+05	5.09E+05	4.97E+05	8.85E+06	6.53E+06	7.17E+06	1.06E+00	1.60E+01	up
252	6-Hydroxykaempferol-7-O-glucoside	4.64E+02	C21H20O12	[M+H]+	2.41E+05	4.14E+05	3.38E+05	1.23E+06	1.16E+06	1.51E+06	1.03E+00	3.93E+00	up
253	6-Methoxykaempferol-3-O-glucoside	4.78E+02	C22H22O12	[M+H]+	9.26E+05	1.14E+06	8.38E+05	2.56E+06	2.75E+06	2.48E+06	1.04E+00	2.68E+00	up
254	Isorhamnetin-3-O-Glucoside*	4.78E+02	C22H22O12	[M+H]+	6.61E+05	7.04E+05	7.69E+05	9.15E+06	1.23E+07	8.14E+06	1.06E+00	1.39E+01	up
255	Rhamnetin-3-O-Glucoside*	4.78E+02	C22H22O12	[M+H]+	5.13E+05	7.66E+05	8.32E+05	8.53E+06	1.14E+07	8.87E+06	1.05E+00	1.36E+01	up
256	Isorhamnetin-7-O-glucoside (Brassicin)*	4.78E+02	C22H22O12	[M+H]+	1.36E+06	1.28E+06	1.09E+06	8.51E+06	1.12E+07	9.10E+06	1.06E+00	7.70E+00	up
257	tricin-4'-O-glucoside	4.92E+02	C23H24O12	[M+H]+	9.00E+00	9.00E+00	9.00E+00	1.99E+05	2.02E+05	2.43E+05	1.06E+00	2.38E+04	up
258	Eriodictyol-7-O-(6"-acetyl)glucoside	4.92E+02	C23H24O12	[M-H]-	9.00E+00	9.00E+00	9.00E+00	1.55E+05	1.86E+05	1.62E+05	1.06E+00	1.86E+04	up
259	Laricitrin-3-O-glucoside	4.94E+02	C22H22O13	[M+H]+	9.00E+00	9.00E+00	9.00E+00	1.36E+05	4.80E+05	2.02E+05	1.06E+00	3.03E+04	up
260	Limocitrin-3-O-galactoside*	5.08E+02	C23H24O13	[M+H]+	8.68E+04	1.05E+05	1.06E+05	7.11E+05	8.70E+05	6.77E+05	1.06E+00	7.57E+00	up
261	Genistein-7-O-(6"-malonyl)glucoside	5.18E+02	C24H22O13	[M+H]+	9.00E+00	9.00E+00	9.00E+00	2.69E+05	3.13E+05	2.98E+05	1.06E+00	3.26E+04	up
262	Kaempferol-3-O-(6"-malonyl)galactoside	5.34E+02	C24H22O14	[M+H]+	3.09E+04	5.08E+04	6.52E+04	3.21E+05	3.56E+05	3.05E+05	1.04E+00	6.68E+00	up
263	Tricetin-5-O-(6"-malonyl)glucoside	5.50E+02	C24H22O15	[M+H]+	4.10E+05	3.90E+05	5.11E+05	9.00E+00	9.00E+00	9.00E+00	1.06E+00	2.06E-05	down
264	"vitexin-2""-O-xyloside*"	5.64E+02	C26H28O14	[M+H]+	5.30E+06	6.74E+06	6.96E+06	1.36E+07	1.49E+07	1.45E+07	1.04E+00	2.26E+00	up
265	Apigenin-6-C-(2"-xylosyl)glucoside*	5.64E+02	C26H28O14	[M+H]+	5.30E+06	6.74E+06	6.96E+06	1.36E+07	1.49E+07	1.45E+07	1.04E+00	2.26E+00	up
266	Hispidulin-8-C-(2"-O-xylosyl)xyloside	5.64E+02	C26H28O14	[M+H]+	2.99E+04	3.05E+04	3.73E+04	1.39E+05	1.38E+05	1.47E+05	1.06E+00	4.34E+00	up
267	Apigenin-6-C-(2"-glucosyl)arabinoside	5.64E+02	C26H28O14	[M+H]+	2.19E+04	8.89E+03	2.07E+04	1.51E+05	1.79E+05	1.69E+05	1.03E+00	9.67E+00	up
268	Apigenin-8-C-(2"-xylosyl)glucoside*	5.64E+02	C26H28O14	[M+H]+	5.30E+06	6.74E+06	6.96E+06	1.36E+07	1.49E+07	1.45E+07	1.04E+00	2.26E+00	up
269	Isovitexin-2"xyloside*	5.64E+02	C26H28O14	[M+H]+	5.12E+06	6.38E+06	7.37E+06	1.22E+07	1.61E+07	1.59E+07	1.01E+00	2.34E+00	up
270	Genistein-8-C-apiosyl(1→6)glucoside	5.64E+02	C26H28O14	[M+H]+	5.71E+06	6.22E+06	6.35E+06	1.05E+07	1.51E+07	1.46E+07	1.02E+00	2.20E+00	up
271	Isovitexin-8-O-xylcoside*	5.64E+02	C26H28O14	[M+H]+	1.08E+06	1.22E+06	1.49E+06	2.80E+06	3.44E+06	3.14E+06	1.03E+00	2.47E+00	up
272	Apigenin-6-C-(2"-glucuronyl)xyloside	5.78E+02	C26H26O15	[M+H]+	2.16E+05	2.75E+05	2.65E+05	9.70E+05	1.23E+06	1.04E+06	1.05E+00	4.28E+00	up
273	Apigenin-7-O-(6"-p-Coumaryl)glucoside	5.78E+02	C30H26O12	[M+H]+	5.89E+06	4.96E+06	4.66E+06	2.00E+06	2.44E+06	2.26E+06	1.04E+00	4.32E-01	down
274	Apigenin-7-O-neohesperidoside (Rhoifolin)*	5.78E+02	C27H30O14	[M+H]+	5.67E+06	4.53E+06	4.87E+06	2.22E+06	2.67E+06	2.39E+06	1.03E+00	4.83E-01	down
275	Genistein-7-O-galactoside-rhamnose	5.78E+02	C27H30O14	[M+H]+	6.58E+06	6.44E+06	5.64E+06	2.12E+06	3.13E+06	2.32E+06	1.02E+00	4.05E-01	down
276	Apigenin-7-O-rutinoside (Isorhoifolin)*	5.78E+02	C27H30O14	[M+H]+	5.01E+06	7.34E+06	6.69E+06	2.15E+06	2.87E+06	2.42E+06	1.02E+00	3.91E-01	down

277	Isovitexin-2''-O-rhamnoside	5.78E+02	C27H30O14	[M+H]+	2.99E+05	2.37E+05	3.12E+05	7.99E+05	1.01E+06	9.44E+05	1.04E+00	3.25E+00	up
278	orientin-2''-O-xyloside	5.80E+02	C26H28O15	[M+H]+	1.64E+05	1.78E+05	1.65E+05	3.43E+05	3.43E+05	3.41E+05	1.06E+00	2.03E+00	up
279	isocarlinoside	5.80E+02	C26H28O15	[M+H]+	5.49E+04	5.88E+04	6.15E+04	1.11E+05	1.20E+05	1.39E+05	1.04E+00	2.12E+00	up
280	Luteolin-6-C-arabinoside-7-O-glucoside	5.80E+02	C26H28O15	[M+H]+	1.69E+06	1.39E+06	1.09E+06	1.42E+05	1.76E+05	1.68E+05	1.05E+00	1.16E-01	down
281	Naringenin-7-O-Rutinoside(Narirutin)*	5.80E+02	C27H32O14	[M+H]+	9.10E+06	1.15E+07	1.01E+07	2.78E+06	3.06E+06	3.13E+06	1.05E+00	2.92E-01	down
282	Naringenin-7-O-Neohesperidoside(Naringin)*	5.80E+02	C27H32O14	[M-H]-	6.44E+07	6.58E+07	6.21E+07	1.75E+06	1.58E+05	1.56E+06	1.00E+00	1.80E-02	down
283	Luteolin-6-C-(5''-glucuronyl)xyloside	5.94E+02	C26H26O16	[M+H]+	3.33E+04	3.52E+04	4.56E+04	1.13E+05	1.74E+05	1.92E+05	1.03E+00	4.20E+00	up
284	Luteolin-7-O-(6''-caffeoyl)rhamnoside	5.94E+02	C30H26O13	[M-H]-	6.83E+06	8.05E+06	8.07E+06	1.14E+06	1.44E+06	1.39E+06	1.06E+00	1.73E-01	down
285	Kaempferol-3-O-(2''-p-Coumaroyl)galactoside	5.94E+02	C30H26O13	[M+H]+	2.64E+07	3.51E+07	3.18E+07	5.29E+06	6.56E+06	5.21E+06	1.05E+00	1.83E-01	down
286	Kaempferol-3-O-(6''-p-Coumaroyl)glucoside (Tiliroside)	5.94E+02	C30H26O13	[M-H]-	2.49E+07	3.37E+07	2.59E+07	4.35E+06	4.38E+06	4.72E+06	1.06E+00	1.59E-01	down
287	Diosmetin-8-C-(2''-O-arabinosyl)glucoside	5.94E+02	C27H30O15	[M+H]+	2.63E+05	3.84E+05	4.10E+05	1.06E+06	1.09E+06	1.02E+06	1.03E+00	3.00E+00	up
288	Hispidulin-8-C-(2''-O-xylosyl)glucoside	5.94E+02	C27H30O15	[M+H]+	6.47E+05	8.65E+05	1.04E+06	2.14E+06	2.90E+06	2.63E+06	1.02E+00	3.00E+00	up
289	Kaempferol-3-O-robinobioside(Biorobin)*	5.94E+02	C27H30O15	[M-H]-	1.80E+07	2.05E+07	1.83E+07	9.00E+00	9.00E+00	9.00E+00	1.06E+00	4.76E-07	down
290	IsoSaponarin(Isovitexin-4'-O-glucoside)*	5.94E+02	C27H30O15	[M+H]+	2.25E+05	2.82E+05	3.02E+05	8.67E+05	1.35E+06	1.06E+06	1.04E+00	4.05E+00	up
291	5,7,4'-Trihydroxy-8-methoxyflavone-6-C-[Xylosyl -(1-2)]-glucoside	5.94E+02	C27H30O15	[M+H]+	6.47E+05	8.65E+05	1.04E+06	2.14E+06	2.90E+06	2.63E+06	1.02E+00	3.00E+00	up
292	Poncirin (Isosakuranetin-7-O-neohesperidoside)	5.94E+02	C28H34O14	[M-H]-	2.27E+07	3.41E+07	2.83E+07	4.38E+06	4.67E+06	5.21E+06	1.05E+00	1.68E-01	down
293	Didymin (Isosakuranetin-7-O-rutinoside)	5.94E+02	C28H34O14	[M+H]+	2.59E+07	3.39E+07	2.93E+07	5.51E+06	7.42E+06	6.31E+06	1.05E+00	2.16E-01	down
294	Neoeriocitrin	5.96E+02	C27H32O15	[M-H]-	2.44E+07	2.60E+07	2.60E+07	3.01E+06	3.45E+06	3.19E+06	1.06E+00	1.26E-01	down
295	Eriodictyol-7-O-Rutinoside (Eriocitrin)	5.96E+02	C27H32O15	[M+H]+	2.57E+07	7.58E+07	6.63E+07	2.19E+06	3.40E+06	3.26E+06	1.03E+00	5.27E-02	down
296	Diosmetin-8-C-(2''-O-rhamnosyl)glucoside	6.08E+02	C28H32O15	[M+H]+	3.65E+04	2.08E+04	4.22E+04	1.78E+05	1.95E+05	2.30E+05	1.03E+00	6.06E+00	up
297	Kaempferol-3-O-galactoside-4'-O-glucoside	6.10E+02	C27H30O16	[M+H]+	9.10E+05	8.00E+05	7.79E+05	9.27E+04	1.45E+05	1.11E+05	1.05E+00	1.40E-01	down
298	Isoorientin-7-O-glucoside	6.10E+02	C27H30O16	[M+H]+	9.00E+00	9.00E+00	9.00E+00	1.19E+05	3.40E+04	3.59E+04	1.06E+00	6.99E+03	up
299	Quercetin-3-O-neohesperidoside*	6.10E+02	C27H30O16	[M+H]+	9.00E+00	9.00E+00	9.00E+00	2.57E+06	3.18E+06	2.20E+06	1.06E+00	2.95E+05	up
300	Quercetin-3-O-rutinoside (Rutin)*	6.10E+02	C27H30O16	[M-H]-	1.92E+06	1.84E+06	1.77E+06	1.40E+05	1.25E+05	1.28E+05	1.06E+00	7.10E-02	down
301	Luteolin-6,8-di-C-glucoside*	6.10E+02	C27H30O16	[M+H]+	1.98E+06	1.94E+06	2.35E+06	8.02E+06	1.01E+07	8.18E+06	1.05E+00	4.19E+00	up

302	Luteolin-7,3'-di-O-glucoside	6.10E+02	C27H30O16	[M+H]+	1.26E+06	1.21E+06	1.10E+06	1.82E+05	2.21E+05	1.84E+05	1.06E+00	1.64E-01	down
303	Quercetin-3-O-(4"-O-glucosyl)rhamnoside*	6.10E+02	C27H30O16	[M+H]+	4.26E+06	4.70E+06	4.43E+06	9.28E+05	1.01E+06	8.26E+05	1.06E+00	2.06E-01	down
304	Quercetin-3-O-robinobioside*	6.10E+02	C27H30O16	[M-H]-	4.99E+04	5.19E+04	6.94E+04	5.57E+05	7.04E+05	5.79E+05	1.06E+00	1.07E+01	up
305	Kaempferol-6,8-di-C-glucoside	6.10E+02	C27H30O16	[M+H]+	7.93E+05	9.47E+05	1.16E+06	2.79E+06	3.41E+06	2.78E+06	1.04E+00	3.10E+00	up
306	Hesperetin-7-O-neohesperidoside(Neohesperidin)*	6.10E+02	C28H34O15	[M+H]+	9.25E+07	9.60E+07	8.86E+07	9.74E+06	1.44E+07	1.23E+07	1.06E+00	1.32E-01	down
307	Hesperetin-7-O-rutinoside (Hesperidin)*	6.10E+02	C28H34O15	[M+H]+	8.34E+07	9.67E+07	9.25E+07	1.09E+07	1.48E+07	1.10E+07	1.06E+00	1.35E-01	down
308	Luteolin-6-C-(2"-glucuronyl)glucoside	6.24E+02	C27H28O17	[M+H]+	7.77E+05	9.04E+05	9.12E+05	2.73E+06	3.03E+06	3.12E+06	1.06E+00	3.42E+00	up
309	Chrysoeriol-6,8-di-C-glucoside	6.24E+02	C28H32O16	[M+H]+	2.60E+06	3.33E+06	3.26E+06	1.20E+07	1.18E+07	1.20E+07	1.06E+00	3.90E+00	up
310	Hispidulin-8-C-(2"-O-glucosyl)glucoside	6.24E+02	C28H32O16	[M+H]+	1.37E+05	1.55E+05	1.06E+05	5.80E+05	6.05E+05	6.18E+05	1.05E+00	4.53E+00	up
311	Isorhamnetin-3-O-neohesperidoside*	6.24E+02	C28H32O16	[M+H]+	3.98E+05	4.05E+05	3.83E+05	2.06E+06	3.05E+06	3.14E+06	1.05E+00	6.95E+00	up
312	6-C-Methylquercetin-3-O-rutinoside	6.24E+02	C28H32O16	[M+H]+	3.98E+05	4.05E+05	3.83E+05	2.06E+06	3.05E+06	3.14E+06	1.05E+00	6.95E+00	up
313	Isorhamnetin-3-O-glucoside-7-O-rhamnoside*	6.24E+02	C28H32O16	[M+H]+	5.19E+05	4.52E+05	4.20E+05	2.28E+06	2.73E+06	2.96E+06	1.06E+00	5.73E+00	up
314	Isorhamnetin-3-O-galactoide-7-O-rhamnoside*	6.24E+02	C28H32O16	[M+H]+	5.19E+05	4.52E+05	4.20E+05	2.28E+06	2.73E+06	2.96E+06	1.06E+00	5.73E+00	up
315	Methylhesperidin	6.24E+02	C29H36O15	[M-H]-	3.30E+04	3.43E+04	3.64E+04	4.15E+05	3.93E+05	3.89E+05	1.06E+00	1.15E+01	up
316	6-Hydroxykaempferol-7,6-O-Diglucoside*	6.26E+02	C27H30O17	[M+H]+	6.31E+05	8.18E+05	6.69E+05	1.50E+05	1.59E+05	1.46E+05	1.06E+00	2.15E-01	down
317	Meratin	6.26E+02	C27H30O17	[M+H]+	6.27E+05	7.72E+05	6.47E+05	1.43E+05	1.41E+05	1.38E+05	1.06E+00	2.06E-01	down
318	Quercetin3,7-diglucoside	6.26E+02	C27H30O17	[M+H]+	6.24E+05	6.80E+05	6.08E+05	1.60E+05	1.80E+05	1.65E+05	1.06E+00	2.64E-01	down
319	6-Hydroxykaempferol-3,6-O-Diglucoside*	6.26E+02	C27H30O17	[M+H]+	1.81E+05	1.70E+05	2.13E+05	6.08E+05	7.17E+05	7.50E+05	1.05E+00	3.68E+00	up
320	Viscumneoside IV(Rhamnazin-3-O-(6"-hydroxymethylglutaryl)glucoside)	6.36E+02	C29H32O16	[M+H]+	9.00E+00	9.00E+00	9.00E+00	1.66E+05	1.66E+05	1.86E+05	1.06E+00	1.92E+04	up
321	Isorhamnetin-3,7-O-diglucoside	6.40E+02	C28H32O17	[M+H]+	6.10E+05	6.95E+05	7.81E+05	1.36E+05	1.64E+05	1.29E+05	1.05E+00	2.06E-01	down
322	2'-Hydoxy,5-methoxyGenistein-4',7-O-diglucoside	6.40E+02	C28H32O17	[M+H]+	1.08E+06	9.31E+05	1.03E+06	1.98E+06	2.56E+06	2.23E+06	1.04E+00	2.22E+00	up
323	Patuletin-3-O-rutinoside	6.40E+02	C28H32O17	[M+H]+	8.90E+05	1.20E+06	1.21E+06	2.37E+06	2.31E+06	2.31E+06	1.03E+00	2.12E+00	up
324	Limocitrin-3-O-(3-hydroxy-3-methylglutarate)glucoside	6.52E+02	C29H32O17	[M+H]+	3.83E+05	3.66E+05	4.25E+05	3.01E+06	3.40E+06	3.53E+06	1.06E+00	8.46E+00	up

325	3,3',5,7-Tetrahydroxy-4',6-Dimethoxyflavone-7-O-Gentiobioside; (Laciniatin-7-O-Gentiobioside)	6.70E+02	C29H34O18	[M+H]+	1.38E+05	1.49E+05	1.71E+05	3.04E+05	4.77E+05	3.53E+05	1.01E+00	2.48E+00	up
326	Natsudaidain-3-O-(3-hydroxy-3-methylglutarate)glucoside	7.24E+02	C33H40O18	[M+H]+	1.87E+05	2.03E+05	2.04E+05	9.55E+06	9.34E+06	9.63E+06	1.06E+00	4.81E+01	up
327	Luteolin-7-O-(2''-O-rhamnosyl)rutinoside	7.40E+02	C33H40O19	[M+H]+	1.66E+06	2.52E+06	1.71E+06	2.27E+05	3.40E+05	2.72E+05	1.05E+00	1.42E-01	down
328	Kaempferol-3-O-rutinoside-7-O-rhamnoside	7.40E+02	C33H40O19	[M+H]+	1.47E+06	1.30E+06	1.07E+06	4.78E+06	5.81E+06	4.95E+06	1.05E+00	4.04E+00	up
329	Clitorin	7.40E+02	C33H40O19	[M+H]+	1.47E+06	1.30E+06	1.07E+06	4.78E+06	5.81E+06	4.95E+06	1.05E+00	4.04E+00	up
330	Kaempferol-3-O-robinoside-7-O-rhamnoside (Robinin)	7.40E+02	C33H40O19	[M+H]+	1.53E+06	2.16E+06	1.91E+06	2.18E+05	2.07E+05	1.59E+05	1.05E+00	1.04E-01	down
331	Naringenin-7-O-Rutinoside-4'-O-glucoside	7.42E+02	C33H42O19	[M-H]-	2.75E+07	3.16E+07	3.11E+07	3.95E+05	4.62E+05	4.81E+05	1.06E+00	1.48E-02	down
332	Apigenin-7-O-rutinoside-4'-O-glucuronide	7.54E+02	C33H38O20	[M+H]+	2.98E+04	3.32E+04	2.79E+04	1.96E+05	1.71E+05	1.58E+05	1.06E+00	5.78E+00	up
333	Kaempferol-3-O-neohesperidoside-7-O-glucoside	7.56E+02	C33H40O20	[M+H]+	3.25E+06	2.87E+06	2.76E+06	1.50E+05	1.91E+05	1.60E+05	1.06E+00	5.66E-02	down
334	Quercetin-3-O-(2''-O-Rhamnosyl)rutinoside	7.56E+02	C33H40O20	[M+H]+	9.21E+05	8.91E+05	8.07E+05	4.44E+06	4.14E+06	3.99E+06	1.06E+00	4.80E+00	up
335	Quercetin-3-O-Sambubioside-5-O-Glucoside	7.58E+02	C32H38O21	[M+H]+	3.96E+04	6.15E+04	4.83E+04	2.15E+05	2.35E+05	2.26E+05	1.05E+00	4.53E+00	up
336	Isovitexin-7-O-(6''-feruloyl)glucoside*	7.70E+02	C37H38O18	[M+H]+	9.46E+04	1.08E+05	7.72E+04	2.46E+05	3.05E+05	2.75E+05	1.04E+00	2.95E+00	up
337	vitexin-7-O-(6''-feruloyl)glucoside*	7.70E+02	C37H38O18	[M+H]+	3.87E+04	4.51E+04	4.20E+04	1.55E+05	1.61E+05	1.22E+05	1.05E+00	3.48E+00	up
338	Rhamnetin-3-O-Rutinoside-5-O-rhamnoside*	7.70E+02	C34H42O20	[M+H]+	1.34E+06	1.57E+06	1.22E+06	6.94E+06	6.55E+06	5.86E+06	1.06E+00	4.69E+00	up
339	Chrysoeriol-7-O-rutinoside-5-O-glucoside	7.70E+02	C34H42O20	[M+H]+	2.82E+04	4.67E+04	4.30E+04	1.87E+05	1.55E+05	1.74E+05	1.04E+00	4.38E+00	up
340	Typhaneoside	7.70E+02	C34H42O20	[M+H]+	1.39E+06	1.44E+06	1.42E+06	4.93E+06	6.43E+06	4.63E+06	1.05E+00	3.76E+00	up
341	Isorhamnetin-3-O-rutinoside-7-O-rhamnoside*	7.70E+02	C34H42O20	[M+H]+	1.12E+06	1.26E+06	9.75E+05	4.71E+06	6.73E+06	5.93E+06	1.05E+00	5.18E+00	up
342	6-Hydroxykaempferol 3-Rutinoside-6-glucoside	7.72E+02	C33H40O21	[M+H]+	3.92E+06	4.47E+06	4.09E+06	2.64E+05	3.38E+05	2.72E+05	1.06E+00	7.01E-02	down
343	Quercetin-3-O-rutinoside-7-O-glucoside	7.72E+02	C33H40O21	[M+H]+	4.65E+06	4.98E+06	5.14E+06	2.76E+05	4.41E+05	3.60E+05	1.06E+00	7.29E-02	down
344	Quercetin-3-O-sophoroside-7-O-rhamnoside	7.72E+02	C33H40O21	[M+H]+	1.77E+06	2.04E+06	1.90E+06	2.43E+04	1.99E+04	2.64E+04	1.06E+00	1.23E-02	down
345	Kaempferol-6,8-di-C-glucoside-7-O-glucoside	7.72E+02	C33H40O21	[M+H]+	1.46E+06	1.52E+06	1.76E+06	1.03E+05	1.56E+05	1.21E+05	1.06E+00	8.02E-02	down
346	Isorhamnetin-3-O-rutinoside-4'-O-glucoside*	7.86E+02	C34H42O21	[M+H]+	3.58E+06	4.43E+06	4.71E+06	2.62E+05	3.36E+05	2.87E+05	1.06E+00	6.97E-02	down
347	Isorhamnetin-3-O-sophoroside-7-O-rhamnoside*	7.86E+02	C34H42O21	[M+H]+	3.63E+06	4.66E+06	4.53E+06	2.95E+05	3.70E+05	2.96E+05	1.06E+00	7.50E-02	down
348	Chrysoeriol-6,8-di-C-glucoside-4'-O-glucoside	7.86E+02	C34H42O21	[M+H]+	1.77E+06	2.31E+06	2.11E+06	1.26E+05	2.12E+05	1.51E+05	1.05E+00	7.91E-02	down

349	Chrysoeriol-8-C-glucoside-7-O-(6"-feruloyl)glucoside	8.00E+02	C38H40O19	[M+H]+	2.73E+04	1.83E+04	1.77E+04	2.10E+05	1.89E+05	2.12E+05	1.05E+00	9.63E+00	up
350	Natsudaidain-3-O-(5'-glucosyl-3-hydroxy-3-methylglutarate)glucoside	8.86E+02	C39H50O23	[M+H]+	3.06E+03	4.36E+03	4.45E+03	2.41E+04	3.49E+04	3.44E+04	1.05E+00	7.86E+00	up
**Lignans and Coumarins**													
351	4-Hydroxycoumarin	1.62E+02	C9H6O3	[M-H]-	9.00E+00	9.00E+00	9.00E+00	4.87E+06	3.88E+06	4.56E+06	1.06E+00	4.93E+05	up
352	Psoralen*	1.86E+02	C11H6O3	[M+H]+	9.00E+00	9.00E+00	9.00E+00	4.37E+04	4.05E+04	5.38E+04	1.06E+00	5.11E+03	up
353	Angelicin*	1.86E+02	C11H6O3	[M+H]+	9.00E+00	9.00E+00	9.00E+00	5.00E+04	5.27E+04	4.67E+04	1.06E+00	5.53E+03	up
354	Scopoletin (7-Hydroxy-6-methoxycoumarin)*	1.92E+02	C10H8O4	[M+H]+	9.00E+00	9.00E+00	9.00E+00	7.96E+06	1.05E+07	1.03E+07	1.06E+00	1.06E+06	up
355	isoscopoletin*	1.92E+02	C10H8O4	[M+H]+	2.45E+04	2.43E+04	2.83E+04	9.57E+06	1.11E+07	9.65E+06	1.06E+00	3.93E+02	up
356	Xanthotoxol	2.02E+02	C11H6O4	[M+H]+	9.00E+00	9.00E+00	9.00E+00	1.31E+07	1.22E+07	1.33E+07	1.06E+00	1.43E+06	up
357	Bergaptol	2.02E+02	C11H6O4	[M+H]+	9.00E+00	9.00E+00	9.00E+00	3.42E+06	3.58E+06	3.65E+06	1.06E+00	3.94E+05	up
358	Fraxetin (7,8-Dihydroxy-6-methoxycoumarin)	2.08E+02	C10H8O5	[M+H]+	5.92E+04	1.28E+05	5.57E+04	4.50E+05	4.38E+05	3.31E+05	1.01E+00	5.02E+00	up
359	isofraxidin*	2.22E+02	C11H10O5	[M+H]+	9.00E+00	9.00E+00	9.00E+00	2.05E+05	2.52E+05	2.31E+05	1.06E+00	2.55E+04	up
360	Fraxidin (8-Hydroxy-6,7-dimethoxycoumarin)*	2.22E+02	C11H10O5	[M+H]+	3.74E+03	4.74E+03	3.02E+03	2.25E+05	2.48E+05	2.73E+05	1.06E+00	6.49E+01	up
361	Sideretin (5,7,8-Trihydroxy-6-methoxycoumarin)	2.24E+02	C10H8O6	[M-H]-	1.97E+05	9.29E+04	1.08E+05	3.01E+07	3.26E+07	3.24E+07	1.06E+00	2.39E+02	up
362	5,6,7-Trimethoxycoumarin*	2.36E+02	C12H12O5	[M+H]+	9.00E+00	9.00E+00	9.00E+00	1.87E+05	1.79E+05	1.85E+05	1.06E+00	2.04E+04	up
363	5,7,8-trimethoxycoumarin*	2.36E+02	C12H12O5	[M+H]+	8.48E+03	1.06E+04	7.01E+03	6.55E+04	6.48E+04	6.10E+04	1.05E+00	7.33E+00	up
364	Osthol	2.44E+02	C15H16O3	[M+H]+	2.62E+05	1.89E+05	2.31E+05	1.60E+06	1.62E+06	1.70E+06	1.06E+00	7.22E+00	up
365	Suberosin	2.44E+02	C15H16O3	[M+H]+	4.27E+05	3.07E+05	3.76E+05	2.53E+06	2.60E+06	2.41E+06	1.06E+00	6.80E+00	up
366	S-(+)-Marmesin	2.46E+02	C14H14O4	[M+H]+	3.23E+04	4.24E+04	3.48E+04	5.71E+05	5.74E+05	6.98E+05	1.06E+00	1.68E+01	up
367	7,8-Dihydroxy-4-phenylcoumarin	2.54E+02	C15H10O4	[M+H]+	9.00E+00	9.00E+00	9.00E+00	7.30E+04	6.06E+04	6.50E+04	1.06E+00	7.36E+03	up
368	Auraptenol	2.60E+02	C15H16O4	[M+H]+	5.33E+05	6.30E+05	5.19E+05	8.21E+06	7.20E+06	7.84E+06	1.06E+00	1.38E+01	up
369	Sibiricol	2.60E+02	C15H16O4	[M-H]-	9.00E+00	9.00E+00	9.00E+00	1.16E+04	1.25E+04	1.10E+04	1.06E+00	1.30E+03	up
370	Meranzin	2.60E+02	C15H16O4	[M+H]+	7.40E+04	9.41E+04	9.55E+04	2.35E+06	2.26E+06	2.34E+06	1.06E+00	2.64E+01	up
371	7-Methoxy-5-Prenyloxycoumarin	2.60E+02	C15H16O4	[M+H]+	3.31E+05	3.97E+05	3.99E+05	7.81E+06	7.21E+06	7.10E+06	1.06E+00	1.96E+01	up
372	(S)-Peucedanol	2.64E+02	C14H16O5	[M+H]+	1.38E+05	1.18E+05	1.66E+05	4.20E+06	5.14E+06	4.20E+06	1.06E+00	3.21E+01	up

373	Dimethoxysamin	2.66E+02	C14H18O5	[M+H]+	5.62E+04	5.54E+04	6.93E+04	3.90E+05	4.85E+05	4.32E+05	1.06E+00	7.23E+00	up
374	8-Isopentenyllimettin	2.74E+02	C16H18O4	[M+H]+	1.03E+04	7.25E+03	1.20E+04	3.53E+04	3.75E+04	3.05E+04	1.03E+00	3.50E+00	up
375	(S)-Murpanidin	2.76E+02	C15H16O5	[M+H]+	9.00E+00	9.00E+00	9.00E+00	8.93E+04	8.77E+04	7.21E+04	1.06E+00	9.23E+03	up
376	(±)-Murpaniculol	2.76E+02	C15H16O5	[M+H]+	9.00E+00	9.00E+00	9.00E+00	8.93E+04	8.77E+04	7.21E+04	1.06E+00	9.23E+03	up
377	Oxypeucedanin*	2.86E+02	C16H14O5	[M+H]+	9.00E+00	9.00E+00	9.00E+00	1.63E+06	1.57E+06	1.73E+06	1.06E+00	1.83E+05	up
378	Isooxypeucedanine*	2.86E+02	C16H14O5	[M+H]+	9.00E+00	9.00E+00	9.00E+00	1.63E+06	1.57E+06	1.73E+06	1.06E+00	1.83E+05	up
379	5,7-Dimethoxy-8-(3'-methyl-2'-oxobutyl)coumarin *	2.90E+02	C16H18O5	[M+H]+	1.59E+05	2.14E+05	2.15E+05	9.00E+06	9.68E+06	9.58E+06	1.06E+00	4.80E+01	up
380	aculeatin	2.90E+02	C16H18O5	[M+H]+	9.00E+00	9.00E+00	9.00E+00	7.05E+04	7.39E+04	8.72E+04	1.06E+00	8.58E+03	up
381	Auraptene	2.98E+02	C19H22O3	[M+H]+	4.20E+07	2.35E+07	2.69E+07	4.04E+05	3.40E+05	2.83E+05	1.06E+00	1.11E-02	down
382	Isomexoticin	3.08E+02	C16H20O6	[M+H]+	9.29E+03	6.49E+03	1.20E+04	5.85E+05	4.56E+05	5.08E+05	1.06E+00	5.57E+01	up
383	5-Geranyloxy-1,3-dihydroxyxanthone	3.14E+02	C19H22O4	[M+H]+	9.00E+00	9.00E+00	9.00E+00	5.47E+04	5.28E+04	5.06E+04	1.06E+00	5.85E+03	up
384	Marmin [7-(6',7'-Dihydroxygeranyloxy)coumarin]	3.32E+02	C19H24O5	[M+H]+	2.31E+06	1.04E+06	2.47E+06	4.13E+07	3.94E+07	4.24E+07	1.05E+00	2.12E+01	up
385	Bergamottin	3.38E+02	C21H22O4	[M+H]+	1.46E+06	5.58E+05	1.10E+06	1.23E+03	1.52E+03	3.74E+03	1.05E+00	2.09E-03	down
386	Bavacumestan A	3.52E+02	C20H16O6	[M+H]+	9.00E+00	9.00E+00	9.00E+00	2.04E+06	1.99E+06	1.96E+06	1.06E+00	2.22E+05	up
387	Pinoresinol*	3.58E+02	C20H22O6	[M-H]-	8.52E+03	1.16E+04	6.32E+03	7.06E+06	7.02E+06	6.77E+06	1.06E+00	7.88E+02	up
388	Epipinoresinol*	3.58E+02	C20H22O6	[M-H]-	1.04E+04	6.34E+03	5.25E+03	6.30E+06	6.52E+06	6.50E+06	1.06E+00	8.78E+02	up
389	Cnidioside A	3.68E+02	C17H20O9	[M-H]-	5.56E+04	3.87E+04	5.60E+04	7.10E+05	7.37E+05	7.09E+05	1.06E+00	1.43E+01	up
390	8-Hydroxy-α-conidendrin	3.72E+02	C20H20O7	[M-H]-	9.00E+00	9.00E+00	9.00E+00	4.71E+05	6.97E+05	5.49E+05	1.06E+00	6.36E+04	up
391	6',7'-Dihydroxybergamottin	3.72E+02	C21H24O6	[M+H]+	2.46E+06	1.60E+06	3.63E+06	9.00E+00	9.00E+00	9.00E+00	1.06E+00	3.51E-06	down
392	Isohydroxymatairesinol	3.74E+02	C20H22O7	[M-H]-	9.00E+00	9.00E+00	9.00E+00	1.43E+05	2.34E+05	1.95E+05	1.06E+00	2.12E+04	up
393	Fraxidin-8-O-glucoside*	3.84E+02	C17H20O10	[M+H]+	9.00E+00	9.00E+00	9.00E+00	8.05E+05	9.97E+05	1.02E+06	1.06E+00	1.05E+05	up
394	Methylpicraquassioside A	4.12E+02	C19H24O10	[M+H]+	7.85E+05	7.99E+05	9.34E+05	6.44E+06	6.39E+06	6.43E+06	1.06E+00	7.65E+00	up
395	Pinoresinol-4-O-glucoside	5.20E+02	C26H32O11	[M-H]-	3.22E+05	2.44E+05	2.29E+05	7.67E+04	1.05E+05	1.21E+05	1.00E+00	3.81E-01	down
396	Isolariciresinol-9'-O-glucoside*	5.22E+02	C26H34O11	[M-H]-	1.82E+05	1.68E+05	1.68E+05	6.65E+05	7.17E+05	6.88E+05	1.06E+00	3.99E+00	up
397	Dihydrodehydrodiconiferyl alcohol-4-O-β-D-glucopyranoside*	5.22E+02	C26H34O11	[M-H]-	1.64E+05	1.65E+05	1.65E+05	6.08E+05	6.81E+05	7.03E+05	1.06E+00	4.03E+00	up

398	5'-Methoxymatairesinoside	5.50E+02	C27H34O12	[M-H]-	2.30E+04	2.00E+04	1.96E+04	6.48E+05	5.87E+05	6.38E+05	1.06E+00	2.99E+01	up
399	5'-Methoxyisolariciresinol-9'-O-glucoside	5.52E+02	C27H36O12	[M-H]-	8.43E+04	7.02E+04	6.56E+04	4.43E+05	4.74E+05	4.57E+05	1.06E+00	6.24E+00	up
400	syringaresinol-4'-O-glucopyranosid*	5.80E+02	C28H36O13	[M-H]-	1.54E+06	1.45E+06	1.59E+06	7.25E+05	8.07E+05	7.26E+05	1.05E+00	4.93E-01	down
**Lipids**													
401	Octanoic acid	1.44E+02	C8H16O2	[M-H]-	9.00E+00	9.00E+00	9.00E+00	1.02E+06	1.01E+06	1.01E+06	1.06E+00	1.12E+05	up
402	10-Hydroxy-2-Decenoic Acid	1.86E+02	C10H18O3	[M-H]-	9.00E+00	9.00E+00	9.00E+00	1.69E+05	1.51E+05	1.62E+05	1.06E+00	1.79E+04	up
403	10-Hydroxydecanoic acid	1.88E+02	C10H20O3	[M-H]-	9.00E+00	9.00E+00	9.00E+00	1.92E+05	1.28E+05	1.58E+05	1.06E+00	1.77E+04	up
404	Dodecanoic acid (Lauric acid)	2.00E+02	C12H24O2	[M-H]-	3.21E+05	2.50E+05	2.46E+05	7.00E+05	7.77E+05	6.58E+05	1.04E+00	2.61E+00	up
405	Undecanedioic acid	2.16E+02	C11H20O4	[M-H]-	4.89E+04	4.76E+04	5.49E+04	3.21E+05	2.93E+05	2.94E+05	1.06E+00	6.00E+00	up
406	12-Hydroxydodecanoic acid	2.16E+02	C12H24O3	[M-H]-	9.00E+00	9.00E+00	9.00E+00	4.92E+04	3.39E+04	3.62E+04	1.06E+00	4.42E+03	up
407	Goshuyic acid	2.24E+02	C14H24O2	[M+H]+	7.96E+04	7.56E+04	1.00E+05	1.08E+06	1.45E+06	1.08E+06	1.06E+00	1.42E+01	up
408	Dodecanedioic aicd	2.30E+02	C12H22O4	[M-H]-	1.29E+03	1.62E+03	1.23E+03	1.45E+04	1.49E+04	1.57E+04	1.06E+00	1.09E+01	up
409	Palmitaldehyde	2.40E+02	C16H32O	[M-H]-	1.80E+07	2.02E+07	1.75E+07	7.20E+06	7.82E+06	7.16E+06	1.06E+00	3.98E-01	down
410	1-O-Caffeoylglycerol	2.54E+02	C12H14O6	[M+H]+	7.68E+03	1.01E+04	2.73E+04	2.17E+05	2.30E+05	2.10E+05	1.02E+00	1.46E+01	up
411	Tetradecanedioic acid	2.58E+02	C14H26O4	[M-H]-	5.03E+04	5.35E+04	5.49E+04	1.16E+05	1.08E+05	1.05E+05	1.06E+00	2.07E+00	up
412	Hexadecylsphingosine	2.73E+02	C16H35NO2	[M+H]+	5.11E+06	5.45E+06	4.94E+06	2.12E+07	1.92E+07	1.86E+07	1.06E+00	3.81E+00	up
413	Crepenynic acid	2.78E+02	C18H30O2	[M-H]-	1.46E+05	1.26E+05	1.19E+05	3.78E+04	3.24E+04	2.10E+04	1.03E+00	2.33E-01	down
414	γ-Linolenic Acid	2.78E+02	C18H30O2	[M-H]-	1.38E+05	1.19E+05	1.10E+05	4.03E+04	3.04E+04	2.31E+04	1.03E+00	2.56E-01	down
415	9,16-Dihydroxypalmitic acid*	2.88E+02	C16H32O4	[M-H]-	9.00E+00	9.00E+00	9.00E+00	1.39E+06	1.24E+06	1.25E+06	1.06E+00	1.44E+05	up
416	10,16-Dihydroxypalmitic acid*	2.88E+02	C16H32O4	[M-H]-	9.00E+00	9.00E+00	9.00E+00	1.04E+05	1.06E+05	1.02E+05	1.06E+00	1.16E+04	up
417	Ricinoleic acid	2.98E+02	C18H34O3	[M-H]-	9.38E+04	8.67E+04	9.36E+04	2.03E+06	7.70E+05	1.24E+06	1.04E+00	1.48E+01	up
418	5S,8R-DiHODE; (5S,8R,9Z,12Z)-5,8-Dihydroxyoctadeca-9,12-dienoate*	3.12E+02	C18H32O4	[M-H]-	9.00E+00	9.00E+00	9.00E+00	9.53E+04	9.29E+04	8.60E+04	1.06E+00	1.02E+04	up
419	9-Hydroxy-12-oxo-15(Z)-octadecenoic acid*	3.12E+02	C18H32O4	[M-H]-	9.00E+00	9.00E+00	9.00E+00	1.45E+06	7.76E+05	1.04E+06	1.06E+00	1.21E+05	up
420	4-Hydroxysphinganine	3.17E+02	C18H39NO3	[M+H]+	2.36E+07	2.46E+07	2.70E+07	6.80E+05	3.90E+05	4.97E+05	1.06E+00	2.08E-02	down
421	9,12,13-Trihydroxy-10,15-octadecadienoic acid	3.28E+02	C18H32O5	[M-H]-	2.71E+04	1.45E+04	8.49E+03	4.29E+05	3.85E+05	4.01E+05	1.04E+00	2.42E+01	up

422	9,10,11-Trihydroxy-12-octadecenoic acid*	3.30E+02	C18H34O5	[M-H]-	2.53E+04	1.37E+04	1.41E+04	3.00E+05	2.79E+05	2.90E+05	1.05E+00	1.64E+01	up
423	9,10,13-Trihydroxy-11-Octadecenoic Acid*	3.30E+02	C18H34O5	[M-H]-	2.35E+05	1.32E+05	1.09E+05	1.18E+06	1.09E+06	1.16E+06	1.04E+00	7.20E+00	up
424	9,10-Dihydroxy-12,13-epoxyoctadecanoic acid	3.30E+02	C18H34O5	[M-H]-	9.00E+00	9.00E+00	9.00E+00	8.86E+05	8.98E+05	1.11E+06	1.06E+00	1.07E+05	up
425	Monopalmitin	3.30E+02	C19H38O4	[M+H]+	1.57E+04	1.18E+04	1.31E+04	7.50E+04	7.04E+04	6.15E+04	1.05E+00	5.09E+00	up
426	1-α-Linolenoyl-glycerol*	3.52E+02	C21H36O4	[M+H]+	3.82E+04	3.17E+04	2.52E+04	9.00E+00	9.00E+00	9.00E+00	1.06E+00	2.84E-04	down
427	1-Linoleoylglycerol*	3.54E+02	C21H38O4	[M+H]+	2.82E+04	2.19E+04	1.72E+04	3.04E+03	2.69E+03	2.89E+03	1.05E+00	1.28E-01	down
428	2-Linoleoylglycerol*	3.54E+02	C21H38O4	[M+H]+	1.69E+05	1.65E+05	1.23E+05	1.72E+03	2.20E+03	1.04E+03	1.06E+00	1.09E-02	down
429	1-Oleoyl-Sn-Glycerol	3.56E+02	C21H40O4	[M+H]+	5.57E+04	5.36E+04	6.40E+04	9.00E+00	9.00E+00	9.00E+00	1.06E+00	1.56E-04	down
430	LysoPE 14:0*	4.25E+02	C19H40NO7P	[M+H]+	1.05E+05	9.09E+04	7.98E+04	9.00E+00	9.00E+00	9.00E+00	1.06E+00	9.79E-05	down
431	LysoPE 14:0(2n isomer)*	4.25E+02	C19H40NO7P	[M+H]+	9.62E+04	8.85E+04	8.27E+04	9.00E+00	9.00E+00	9.00E+00	1.06E+00	1.01E-04	down
432	LysoPE 15:1(2n isomer)*	4.37E+02	C20H40NO7P	[M+H]+	8.93E+04	7.76E+04	6.35E+04	9.00E+00	9.00E+00	9.00E+00	1.06E+00	1.17E-04	down
433	LysoPE 15:1*	4.37E+02	C20H40NO7P	[M+H]+	9.96E+04	8.49E+04	6.44E+04	9.00E+00	9.00E+00	9.00E+00	1.06E+00	1.09E-04	down
434	LysoPE 15:0*	4.39E+02	C20H42NO7P	[M+H]+	5.93E+05	5.00E+05	4.20E+05	9.00E+00	9.00E+00	9.00E+00	1.06E+00	1.78E-05	down
435	LysoPE 15:0(2n isomer)*	4.39E+02	C20H42NO7P	[M+H]+	5.81E+05	4.42E+05	4.12E+05	9.00E+00	9.00E+00	9.00E+00	1.06E+00	1.88E-05	down
436	LysoPE 16:1*	4.51E+02	C21H42NO7P	[M+H]+	2.09E+06	1.82E+06	1.47E+06	9.00E+00	9.00E+00	9.00E+00	1.06E+00	5.02E-06	down
437	LysoPE 16:1(2n isomer)*	4.51E+02	C21H42NO7P	[M+H]+	2.04E+06	1.83E+06	1.53E+06	9.00E+00	9.00E+00	9.00E+00	1.06E+00	5.00E-06	down
438	LysoPE 16:0*	4.53E+02	C21H44NO7P	[M+H]+	2.09E+07	1.96E+07	1.93E+07	2.71E+04	1.34E+04	5.74E+03	1.05E+00	7.73E-04	down
439	LysoPE 16:0(2n isomer)*	4.53E+02	C21H44NO7P	[M+H]+	2.46E+07	2.33E+07	2.23E+07	3.72E+04	1.60E+04	5.40E+03	1.05E+00	8.35E-04	down
440	LysoPE 17:1(2n isomer)*	4.65E+02	C22H44NO7P	[M+H]+	5.32E+05	3.58E+05	3.18E+05	9.00E+00	9.00E+00	9.00E+00	1.06E+00	2.24E-05	down
441	LysoPE 17:1*	4.65E+02	C22H44NO7P	[M+H]+	1.17E+05	1.13E+05	9.78E+04	9.00E+00	9.00E+00	9.00E+00	1.06E+00	8.23E-05	down
442	LysoPC 14:0	4.67E+02	C22H46NO7P	[M+H]+	3.18E+05	2.74E+05	2.20E+05	9.00E+00	9.00E+00	9.00E+00	1.06E+00	3.33E-05	down
443	LysoPE 18:3*	4.75E+02	C23H42NO7P	[M+H]+	8.26E+05	7.62E+05	5.89E+05	9.00E+00	9.00E+00	9.00E+00	1.06E+00	1.24E-05	down
444	LysoPE 18:3(2n isomer)*	4.75E+02	C23H42NO7P	[M+H]+	8.59E+05	6.90E+05	4.69E+05	9.00E+00	9.00E+00	9.00E+00	1.06E+00	1.34E-05	down
445	LysoPC 15:1	4.79E+02	C23H46NO7P	[M+H]+	5.33E+05	5.26E+05	3.30E+05	9.00E+00	9.00E+00	9.00E+00	1.06E+00	1.94E-05	down
446	LysoPE 18:1(2n isomer)*	4.79E+02	C23H46NO7P	[M+H]+	1.42E+07	1.16E+07	1.04E+07	1.45E+04	1.64E+04	1.04E+04	1.06E+00	1.14E-03	down
447	LysoPE 18:1*	4.79E+02	C23H46NO7P	[M+H]+	1.74E+07	1.60E+07	1.42E+07	3.71E+04	1.84E+04	9.33E+03	1.05E+00	1.36E-03	down
448	LysoPC 15:0(2n isomer)*	4.81E+02	C23H48NO7P	[M+H]+	1.26E+05	1.43E+05	1.02E+05	9.00E+00	9.00E+00	9.00E+00	1.06E+00	7.28E-05	down

449	LysoPC 15:0*	4.81E+02	C23H48NO7P	[M+H]+	1.48E+06	1.30E+06	1.07E+06	9.00E+00	9.00E+00	9.00E+00	1.06E+00	7.03E-06	down
450	LysoPC 16:2(2n isomer)*	4.91E+02	C24H46NO7P	[M+H]+	1.76E+05	1.58E+05	1.42E+05	9.00E+00	9.00E+00	9.00E+00	1.06E+00	5.67E-05	down
451	LysoPC 16:1*	4.93E+02	C24H48NO7P	[M+H]+	9.92E+06	8.96E+06	1.01E+07	4.77E+03	4.05E+03	3.63E+03	1.06E+00	4.29E-04	down
452	LysoPC 16:1(2n isomer)*	4.93E+02	C24H48NO7P	[M+H]+	1.01E+07	7.60E+06	8.56E+06	9.00E+00	9.00E+00	9.00E+00	1.06E+00	1.03E-06	down
453	LysoPC 16:0(2n isomer)*	4.95E+02	C24H50NO7P	[M+H]+	4.64E+07	3.47E+07	4.11E+07	8.51E+04	7.91E+04	5.48E+04	1.06E+00	1.79E-03	down
454	LysoPC 16:0*	4.95E+02	C24H50NO7P	[M+H]+	4.68E+07	4.51E+07	4.12E+07	1.17E+05	7.63E+04	6.47E+04	1.06E+00	1.94E-03	down
455	LysoPC 17:2	5.05E+02	C25H48NO7P	[M+H]+	3.65E+05	4.33E+05	2.73E+05	9.00E+00	9.00E+00	9.00E+00	1.06E+00	2.52E-05	down
456	LysoPC 17:1	5.07E+02	C25H50NO7P	[M+H]+	1.17E+06	9.99E+05	8.66E+05	9.00E+00	9.00E+00	9.00E+00	1.06E+00	8.90E-06	down
457	LysoPC 17:0*	5.09E+02	C25H52NO7P	[M+H]+	1.09E+05	9.69E+04	6.61E+04	9.00E+00	9.00E+00	9.00E+00	1.06E+00	9.91E-05	down
458	LysoPC 17:0(2n isomer)*	5.09E+02	C25H52NO7P	[M+H]+	1.43E+05	1.48E+05	7.15E+04	9.00E+00	9.00E+00	9.00E+00	1.06E+00	7.45E-05	down
459	LysoPC 18:3*	5.17E+02	C26H48NO7P	[M+H]+	3.84E+06	3.04E+06	2.78E+06	9.00E+00	9.00E+00	9.00E+00	1.06E+00	2.80E-06	down
460	LysoPC 18:2*	5.19E+02	C26H50NO7P	[M+H]+	3.01E+07	2.96E+07	4.34E+07	5.55E+04	4.03E+04	2.60E+04	1.06E+00	1.18E-03	down
461	LysoPC 18:1(2n isomer)*	5.21E+02	C26H52NO7P	[M+H]+	6.05E+07	5.06E+07	5.38E+07	1.28E+05	5.51E+04	3.15E+04	1.05E+00	1.30E-03	down
462	LysoPC 18:1*	5.21E+02	C26H52NO7P	[M+H]+	6.06E+07	5.38E+07	5.30E+07	9.56E+04	6.44E+04	3.75E+04	1.06E+00	1.18E-03	down
463	LysoPC 18:0*	5.23E+02	C26H54NO7P	[M+H]+	6.66E+05	6.28E+05	5.27E+05	1.63E+04	1.61E+04	1.52E+04	1.06E+00	2.61E-02	down
464	LysoPC 20:1	5.49E+02	C28H56NO7P	[M+H]+	3.11E+05	2.59E+05	3.15E+05	9.00E+00	9.00E+00	9.00E+00	1.06E+00	3.05E-05	down
465	1-Linolenoyl-rac-glycerol-diglucoside	6.76E+02	C33H56O14	[M+H]+	2.64E+05	2.17E+05	2.41E+05	9.00E+00	9.00E+00	9.00E+00	1.06E+00	3.74E-05	down
**Nucleotides and derivatives**													
466	Cytosine	1.11E+02	C4H5N3O	[M+H]+	2.98E+05	3.10E+05	3.44E+05	1.05E+06	9.89E+05	8.43E+05	1.05E+00	3.03E+00	up
467	Uracil	1.12E+02	C4H4N2O2	[M-H]-	1.95E+04	7.37E+04	6.31E+04	1.09E+06	1.16E+06	1.20E+06	1.03E+00	2.21E+01	up
468	5-Methylcytosine	1.25E+02	C5H7N3O	[M+H]+	3.83E+04	4.04E+04	3.45E+04	1.55E+05	1.19E+05	1.14E+05	1.05E+00	3.43E+00	up
469	Thymine	1.26E+02	C5H6N2O2	[M+H]+	9.00E+00	9.00E+00	9.00E+00	3.50E+05	3.02E+05	3.40E+05	1.06E+00	3.68E+04	up
470	Barbituric acid;Malonylurea;2,4,6-Pyrimidinetrione	1.28E+02	C4H4N2O3	[M+H]+	9.00E+00	9.00E+00	9.00E+00	4.09E+06	5.48E+06	3.67E+06	1.06E+00	4.90E+05	up
471	Allopurinol	1.36E+02	C5H4N4O	[M+H]+	9.00E+00	9.00E+00	9.00E+00	2.57E+06	2.79E+06	2.72E+06	1.06E+00	2.99E+05	up
472	Hypoxanthine	1.36E+02	C5H4N4O	[M+H]+	9.00E+00	9.00E+00	9.00E+00	2.80E+06	3.10E+06	2.97E+06	1.06E+00	3.29E+05	up
473	1-Methyladenine	1.49E+02	C6H7N5	[M+H]+	1.23E+05	1.49E+05	2.44E+05	4.71E+06	3.95E+06	4.04E+06	1.05E+00	2.46E+01	up

474	Isoguanine*	1.51E+02	C5H5N5O	[M+H]+	4.94E+04	6.58E+04	4.96E+04	2.69E+07	1.68E+07	2.42E+07	1.06E+00	4.12E+02	up
475	Guanine*	1.51E+02	C5H5N5O	[M+H]+	4.94E+04	6.58E+04	4.96E+04	2.69E+07	1.68E+07	2.42E+07	1.06E+00	4.12E+02	up
476	Xanthine	1.52E+02	C5H4N4O2	[M-H]-	9.00E+00	9.00E+00	9.00E+00	6.35E+05	5.99E+05	1.21E+06	1.06E+00	9.05E+04	up
477	8-Azaguanine	1.52E+02	C4H4N6O	[M+H]+	1.20E+04	1.27E+04	1.46E+04	4.90E+06	2.73E+06	4.25E+06	1.06E+00	3.02E+02	up
478	6-O-methylguanine	1.65E+02	C6H7N5O	[M+H]+	3.29E+04	2.63E+04	3.42E+04	1.60E+06	1.43E+06	1.54E+06	1.06E+00	4.89E+01	up
479	6-Methylmercaptopurine	1.66E+02	C6H6N4S	[M+H]+	1.29E+06	1.54E+06	1.76E+06	8.36E+06	8.04E+06	8.50E+06	1.06E+00	5.42E+00	up
480	2-Deoxyribose-1-phosphate*	2.14E+02	C5H11O7P	[M-H]-	6.45E+04	7.20E+04	8.45E+04	2.90E+05	2.19E+05	2.23E+05	1.04E+00	3.31E+00	up
481	2'-Deoxycytidine	2.27E+02	C9H13N3O4	[M+H]+	4.67E+04	4.76E+04	3.59E+04	2.89E+05	3.31E+05	2.75E+05	1.06E+00	6.87E+00	up
482	Thymidine	2.42E+02	C10H14N2O5	[M+H]+	6.91E+04	1.15E+05	8.12E+04	6.01E+05	8.62E+05	6.92E+05	1.05E+00	8.12E+00	up
483	Cytarabine	2.43E+02	C9H13N3O5	[M+H]+	1.37E+06	1.47E+06	1.41E+06	3.99E+07	3.89E+07	3.84E+07	1.06E+00	2.75E+01	up
484	Uridine	2.44E+02	C9H12N2O6	[M-H]-	4.59E+04	4.18E+04	4.58E+04	8.83E+05	6.78E+05	9.75E+05	1.06E+00	1.90E+01	up
485	β-Pseudouridine	2.44E+02	C9H12N2O6	[M-H]-	9.00E+00	9.00E+00	9.00E+00	9.23E+04	8.34E+04	1.02E+05	1.06E+00	1.03E+04	up
486	1-beta-D-Arabinofuranosyluracil	2.44E+02	C9H12N2O6	[M+H]+	4.16E+05	4.18E+05	3.17E+05	8.84E+05	1.05E+06	9.48E+05	1.04E+00	2.50E+00	up
487	2'-O-Methyladenosine	2.81E+02	C11H15N5O4	[M+H]+	9.00E+00	9.00E+00	9.00E+00	1.76E+06	1.80E+06	2.05E+06	1.06E+00	2.08E+05	up
488	Xanthosine	2.84E+02	C10H12N4O6	[M-H]-	6.01E+04	6.00E+04	7.20E+04	3.14E+05	4.03E+05	3.14E+05	1.05E+00	5.37E+00	up
489	5-Aminoimidazole ribonucleotide	2.95E+02	C8H14N3O7P	[M+H]+	2.15E+06	2.67E+06	2.78E+06	1.25E+07	1.46E+07	1.35E+07	1.06E+00	5.34E+00	up
490	N6-(2-Hydroxyethyl)adenosine	3.11E+02	C12H17N5O5	[M+H]+	2.67E+04	2.74E+04	3.73E+04	2.36E+07	2.26E+07	2.37E+07	1.06E+00	7.65E+02	up
491	2-(Dimethylamino)guanosine	3.11E+02	C12H17N5O5	[M+H]+	3.07E+04	3.48E+04	5.33E+04	2.68E+07	2.78E+07	2.80E+07	1.06E+00	6.95E+02	up
492	Isopentenyladenine-7-N-glucoside	3.65E+02	C16H23N5O5	[M+H]+	9.24E+04	1.06E+05	6.74E+04	9.00E+00	9.00E+00	9.00E+00	1.06E+00	1.02E-04	down
493	Succinyladenosine	3.83E+02	C14H17N5O8	[M+H]+	9.47E+03	9.74E+03	6.94E+03	1.60E+06	1.33E+06	1.64E+06	1.06E+00	1.75E+02	up
494	Ribosyladenosine	3.99E+02	C15H21N5O8	[M+H]+	4.13E+04	5.14E+04	4.07E+04	6.03E+05	6.21E+05	6.00E+05	1.06E+00	1.37E+01	up
**Organic acids**													
495	Oxalic acid	9.00E+01	C2H2O4	[M-H]-	2.96E+04	4.17E+04	1.11E+04	3.67E+05	3.48E+05	3.23E+05	1.02E+00	1.26E+01	up
496	Succinic anhydride	1.00E+02	C4H4O3	[M+H]+	2.06E+05	1.44E+05	1.83E+05	4.68E+06	4.97E+06	4.68E+06	1.06E+00	2.69E+01	up
497	4-Pentenoic acid	1.00E+02	C5H8O2	[M+H]+	2.79E+04	9.48E+03	4.26E+04	5.05E+06	6.44E+06	5.59E+06	1.05E+00	2.13E+02	up
498	2-Aminoisobutyric acid*	1.03E+02	C4H9NO2	[M+H]+	1.34E+06	1.40E+06	1.30E+06	3.70E+05	3.23E+05	2.63E+05	1.05E+00	2.37E-01	down
499	γ-Aminobutyric acid*	1.03E+02	C4H9NO2	[M+H]+	2.54E+06	2.56E+06	2.32E+06	9.18E+05	1.08E+06	9.41E+05	1.05E+00	3.96E-01	down

500	Hydroxypyruvic acid	1.04E+02	C3H4O4	[M-H]-	2.69E+05	2.07E+05	3.21E+05	1.39E+06	1.52E+06	1.50E+06	1.05E+00	5.54E+00	up
501	2-Hydroxybutyric Acid*	1.04E+02	C4H8O3	[M-H]-	9.00E+00	9.00E+00	9.00E+00	4.28E+05	3.39E+05	3.57E+05	1.06E+00	4.16E+04	up
502	2-Hydroxyisobutyric acid*	1.04E+02	C4H8O3	[M-H]-	9.00E+00	9.00E+00	9.00E+00	4.70E+05	3.61E+05	4.06E+05	1.06E+00	4.58E+04	up
503	Malonic acid	1.04E+02	C3H4O4	[M-H]-	7.01E+06	5.64E+06	8.20E+06	1.58E+07	1.82E+07	1.78E+07	1.03E+00	2.49E+00	up
504	Pyrrole-2-carboxylic acid	1.11E+02	C5H5NO2	[M-H]-	9.00E+00	9.00E+00	9.00E+00	4.49E+05	4.41E+05	3.96E+05	1.06E+00	4.76E+04	up
505	3-Furoic acid	1.12E+02	C5H4O3	[M-H]-	9.00E+00	9.00E+00	9.00E+00	4.95E+05	3.74E+05	4.72E+05	1.06E+00	4.96E+04	up
506	3-Methyl-2-Oxobutanoic acid	1.16E+02	C5H8O3	[M-H]-	9.00E+00	9.00E+00	9.00E+00	6.94E+05	1.27E+06	1.12E+06	1.06E+00	1.14E+05	up
507	succinic acid	1.18E+02	C4H6O4	[M-H]-	2.26E+07	1.33E+07	2.07E+07	9.00E+00	9.00E+00	9.00E+00	1.06E+00	4.77E-07	down
508	Methylmalonic acid	1.18E+02	C4H6O4	[M-H]-	2.26E+07	1.33E+07	2.07E+07	9.00E+00	9.00E+00	9.00E+00	1.06E+00	4.77E-07	down
509	β-Hydroxyisovaleric acid	1.18E+02	C5H10O3	[M-H]-	9.74E+04	7.65E+04	7.26E+04	1.26E+06	1.25E+06	1.20E+06	1.06E+00	1.50E+01	up
510	2-Hydroxy-2-methylbutyric acid	1.18E+02	C5H10O3	[M-H]-	9.00E+00	9.00E+00	9.00E+00	3.49E+05	3.39E+05	3.30E+05	1.06E+00	3.77E+04	up
511	Aminomalonic acid	1.19E+02	C3H5NO4	[M-H]-	7.86E+05	4.52E+05	7.27E+05	3.35E+07	3.33E+07	3.60E+07	1.06E+00	5.23E+01	up
512	L-Pipecolic Acid	1.29E+02	C6H11NO2	[M+H]+	1.25E+06	1.30E+06	1.34E+06	5.05E+06	5.06E+06	5.32E+06	1.06E+00	3.97E+00	up
513	Citraconic acid	1.30E+02	C5H6O4	[M-H]-	9.00E+00	9.00E+00	9.00E+00	5.90E+07	5.29E+07	5.86E+07	1.06E+00	6.31E+06	up
514	Methylenesuccinic acid	1.30E+02	C5H6O4	[M-H]-	1.39E+05	1.76E+05	1.84E+05	4.56E+06	5.16E+06	4.34E+06	1.06E+00	2.81E+01	up
515	3-Methyl-2-oxopentanoic acid	1.30E+02	C6H10O3	[M-H]-	9.00E+00	9.00E+00	9.00E+00	3.55E+05	3.48E+05	3.45E+05	1.06E+00	3.88E+04	up
516	3-Guanidinopropionic acid	1.31E+02	C4H9N3O2	[M-H]-	4.14E+04	3.67E+04	4.89E+04	3.28E+05	3.52E+05	3.93E+05	1.06E+00	8.44E+00	up
517	Creatine	1.31E+02	C4H9N3O2	[M-H]-	9.00E+00	9.00E+00	9.00E+00	1.32E+05	1.35E+05	1.34E+05	1.06E+00	1.48E+04	up
518	6-Aminocaproic acid	1.31E+02	C6H13NO2	[M+H]+	8.19E+04	8.44E+04	6.77E+04	3.98E+05	9.83E+05	7.95E+05	1.03E+00	9.31E+00	up
519	Glutaric acid*	1.32E+02	C5H8O4	[M-H]-	9.00E+00	9.00E+00	9.00E+00	4.04E+05	2.93E+05	2.86E+05	1.06E+00	3.64E+04	up
520	2-Methylsuccinic acid*	1.32E+02	C5H8O4	[M-H]-	1.38E+05	1.21E+05	1.34E+05	1.54E+06	1.53E+06	4.69E+06	1.03E+00	1.97E+01	up
521	2-Hydroxy-2-methyl-3-oxobutanoic acid	1.32E+02	C5H8O4	[M-H]-	4.73E+05	3.58E+05	4.48E+05	5.00E+06	4.99E+06	4.38E+06	1.06E+00	1.12E+01	up
522	Dimethylmalonic acid*	1.32E+02	C5H8O4	[M-H]-	4.31E+05	3.78E+05	4.57E+05	4.93E+06	4.81E+06	4.69E+06	1.06E+00	1.14E+01	up
523	Monomethyl succinate	1.32E+02	C5H8O4	[M-H]-	1.38E+05	1.21E+05	1.34E+05	1.54E+06	1.53E+06	4.69E+06	1.03E+00	1.97E+01	up
524	2-Hydroxyisocaproic acid	1.32E+02	C6H12O3	[M-H]-	9.00E+00	9.00E+00	9.00E+00	3.00E+06	2.85E+06	2.95E+06	1.06E+00	3.26E+05	up
525	6-Hydroxyhexanoic acid	1.32E+02	C6H12O3	[M-H]-	9.00E+00	9.00E+00	9.00E+00	6.68E+05	1.33E+06	6.93E+05	1.06E+00	9.97E+04	up
526	2-Hydroxy-4-methylpentanoic acid	1.32E+02	C6H12O3	[M-H]-	9.00E+00	9.00E+00	9.00E+00	2.00E+07	1.82E+07	1.87E+07	1.06E+00	2.11E+06	up

527	Iminodiacetic acid	1.33E+02	C4H7NO4	[M-H]-	1.79E+06	2.11E+06	1.77E+06	2.00E+05	1.55E+05	1.78E+05	1.06E+00	9.41E-02	down
528	5-Hydroxymethyl-2-furancarboxylic acid	1.42E+02	C6H6O4	[M-H]-	9.00E+00	9.00E+00	9.00E+00	8.74E+04	2.55E+04	2.55E+04	1.06E+00	5.13E+03	up
529	2-n-Propyl-3-pentenoic acid*	1.42E+02	C8H14O2	[M-H]-	9.00E+00	9.00E+00	9.00E+00	1.11E+06	9.82E+05	1.01E+06	1.06E+00	1.15E+05	up
530	2-n-Propyl-4-pentenoic acid*	1.42E+02	C8H14O2	[M-H]-	9.00E+00	9.00E+00	9.00E+00	1.11E+06	9.82E+05	1.01E+06	1.06E+00	1.15E+05	up
531	1-Methylpiperidine-2-carboxylic acid	1.43E+02	C7H13NO2	[M+H]+	1.06E+06	1.15E+06	1.13E+06	3.69E+06	3.59E+06	3.55E+06	1.06E+00	3.23E+00	up
532	4-Acetamidobutyric acid	1.45E+02	C6H11NO3	[M+H]+	9.00E+00	9.00E+00	9.00E+00	1.90E+07	1.18E+07	1.50E+07	1.06E+00	1.70E+06	up
533	α-Ketoglutaric acid	1.46E+02	C5H6O5	[M-H]-	2.50E+05	2.33E+05	2.56E+05	5.33E+05	5.76E+05	4.82E+05	1.05E+00	2.15E+00	up
534	Mono-Methyl Glutarate	1.46E+02	C6H10O4	[M-H]-	9.00E+00	9.00E+00	9.00E+00	1.70E+05	1.59E+05	1.62E+05	1.06E+00	1.82E+04	up
535	2-Methylglutaric acid	1.46E+02	C6H10O4	[M-H]-	2.84E+05	2.58E+05	2.65E+05	4.73E+06	4.78E+06	4.63E+06	1.06E+00	1.75E+01	up
536	Adipic Acid	1.46E+02	C6H10O4	[M-H]-	2.84E+05	2.58E+05	2.65E+05	4.73E+06	4.78E+06	4.63E+06	1.06E+00	1.75E+01	up
537	2-Hydroxyglutaric Acid	1.48E+02	C5H8O5	[M-H]-	3.62E+05	4.00E+05	3.55E+05	8.52E+06	7.88E+06	7.02E+06	1.06E+00	2.10E+01	up
538	3-Methylmalic acid	1.48E+02	C5H8O5	[M-H]-	3.62E+05	4.00E+05	3.55E+05	8.52E+06	7.88E+06	7.02E+06	1.06E+00	2.10E+01	up
539	3-Hydroxyglutaric acid	1.48E+02	C5H8O5	[M-H]-	9.00E+00	9.00E+00	9.00E+00	2.44E+07	2.35E+07	2.55E+07	1.06E+00	2.72E+06	up
540	L-Citramalic acid	1.48E+02	C5H8O5	[M-H]-	9.60E+04	1.27E+05	1.21E+05	1.16E+07	1.01E+07	9.98E+06	1.06E+00	9.18E+01	up
541	2,3-Dihydroxy-3-methylpentanoic acid*	1.48E+02	C6H12O4	[M-H]-	9.00E+00	9.00E+00	9.00E+00	7.78E+05	9.94E+05	7.93E+05	1.06E+00	9.50E+04	up
542	2-Hydroxyphenylacetic acid	1.52E+02	C8H8O3	[M-H]-	9.00E+00	9.00E+00	9.00E+00	1.06E+05	6.26E+04	8.49E+04	1.06E+00	9.39E+03	up
543	Phenylpyruvic acid	1.64E+02	C9H8O3	[M-H]-	9.00E+00	9.00E+00	9.00E+00	6.09E+05	6.50E+05	6.13E+05	1.06E+00	6.93E+04	up
544	Sodium Valproate	1.66E+02	C8H15NaO2	[M-Na]-	7.96E+03	8.10E+03	8.46E+03	8.70E+05	9.16E+05	8.78E+05	1.06E+00	1.09E+02	up
545	3-Hydroxymandelate	1.68E+02	C8H8O4	[M-H]-	9.00E+00	9.00E+00	9.00E+00	4.18E+04	5.20E+04	3.59E+04	1.06E+00	4.81E+03	up
546	Decanoic acid	1.72E+02	C10H20O2	[M-H]-	2.13E+03	2.84E+03	3.73E+03	2.08E+05	2.81E+05	2.34E+05	1.06E+00	8.31E+01	up
547	3-Isopropylmalic Acid*	1.76E+02	C7H12O5	[M-H]-	9.00E+00	9.00E+00	9.00E+00	2.45E+07	2.02E+07	2.16E+07	1.06E+00	2.46E+06	up
548	2-Isopropylmalic Acid*	1.76E+02	C7H12O5	[M-H]-	9.00E+00	9.00E+00	9.00E+00	2.40E+07	2.03E+07	2.11E+07	1.06E+00	2.42E+06	up
549	2-Propylmalic Acid*	1.76E+02	C7H12O5	[M-H]-	9.00E+00	9.00E+00	9.00E+00	2.45E+07	2.02E+07	2.16E+07	1.06E+00	2.46E+06	up
550	Azelaic acid	1.88E+02	C9H16O4	[M-H]-	1.78E+06	1.57E+06	1.65E+06	5.42E+06	4.98E+06	4.87E+06	1.06E+00	3.05E+00	up
551	4-Hydroxy-3-methoxymandelate	1.98E+02	C9H10O5	[M-H]-	9.00E+00	9.00E+00	9.00E+00	8.64E+04	8.19E+04	7.69E+04	1.06E+00	9.08E+03	up
552	Sebacate	2.02E+02	C10H18O4	[M-H]-	9.00E+00	9.00E+00	9.00E+00	9.24E+04	8.75E+04	7.67E+04	1.06E+00	9.50E+03	up
553	4,8-Dihydroxyquinoline-2-carboxylic acid	2.05E+02	C10H7NO4	[M-H]-	9.00E+00	9.00E+00	9.00E+00	4.09E+06	2.87E+06	3.43E+06	1.06E+00	3.85E+05	up

554	Jasmonic acid	2.10E+02	C12H18O3	[M-H]-	9.00E+00	9.00E+00	9.00E+00	1.79E+05	1.88E+05	1.76E+05	1.06E+00	2.01E+04	up
555	Abscisic acid	2.64E+02	C15H20O4	[M-H]-	1.48E+06	1.63E+06	1.80E+06	4.95E+06	5.73E+06	5.11E+06	1.05E+00	3.21E+00	up
556	Triethyl citrate	2.76E+02	C12H20O7	[M+H]+	9.00E+00	9.00E+00	9.00E+00	1.69E+05	2.12E+05	1.90E+05	1.06E+00	2.12E+04	up
**Phenolic acids**													
557	Benzamide	1.21E+02	C7H7NO	[M+H]+	9.00E+00	9.00E+00	9.00E+00	6.63E+05	5.50E+05	4.03E+05	1.06E+00	5.98E+04	up
558	benzoic Acid	1.22E+02	C7H6O2	[M+H]+	9.00E+00	9.00E+00	9.00E+00	8.92E+06	8.80E+06	8.31E+06	1.06E+00	9.64E+05	up
559	4-Hydroxybenzaldehyde*	1.22E+02	C7H6O2	[M-H]-	9.00E+00	9.00E+00	9.00E+00	1.11E+06	2.37E+06	1.69E+06	1.06E+00	1.92E+05	up
560	2-Hydroxybenzaldehyde (Salicylaldehyde)*	1.22E+02	C7H6O2	[M-H]-	9.00E+00	9.00E+00	9.00E+00	3.89E+05	3.65E+05	3.86E+05	1.06E+00	4.22E+04	up
561	2-Phenylethanol	1.22E+02	C8H10O	[M+H-H2 O]+	9.00E+00	9.00E+00	9.00E+00	1.07E+06	8.29E+05	8.13E+05	1.06E+00	1.01E+05	up
562	2-Methylbenzoic acid	1.36E+02	C8H8O2	[M-H]-	9.00E+00	9.00E+00	9.00E+00	5.51E+05	5.61E+05	5.53E+05	1.06E+00	6.17E+04	up
563	4-Methoxybenzaldehyde	1.36E+02	C8H8O2	[M+H]+	9.00E+00	9.00E+00	9.00E+00	1.99E+05	2.25E+05	1.85E+05	1.06E+00	2.25E+04	up
564	Phenyl acetate	1.36E+02	C8H8O2	[M-H]-	1.19E+05	1.41E+05	1.24E+05	4.21E+05	3.39E+05	3.62E+05	1.05E+00	2.91E+00	up
565	4-Methylbenzoic Acid	1.36E+02	C8H8O2	[M+H]+	9.00E+00	9.00E+00	9.00E+00	5.31E+05	5.16E+05	5.31E+05	1.06E+00	5.85E+04	up
566	4-Hydroxyacetophenone	1.36E+02	C8H8O2	[M-H]-	1.33E+05	1.34E+05	8.97E+04	4.17E+05	3.53E+05	3.57E+05	1.03E+00	3.16E+00	up
567	4-Hydroxybenzoic Acid	1.38E+02	C7H6O3	[M-H]-	6.52E+03	7.20E+03	3.89E+03	1.08E+06	1.15E+06	1.14E+06	1.06E+00	1.91E+02	up
568	Salicylic acid	1.38E+02	C7H6O3	[M-H]-	2.87E+05	2.46E+05	2.70E+05	3.40E+07	3.22E+07	3.32E+07	1.06E+00	1.24E+02	up
569	Protocatechualdehyde	1.38E+02	C7H6O3	[M-H]-	3.16E+05	2.77E+05	2.71E+05	3.40E+07	3.26E+07	3.41E+07	1.06E+00	1.17E+02	up
570	Tyrosol	1.38E+02	C8H10O2	[M-H]-	9.00E+00	9.00E+00	9.00E+00	3.74E+06	3.46E+06	3.43E+06	1.06E+00	3.94E+05	up
571	Vanillin*	1.52E+02	C8H8O3	[M-H]-	9.00E+00	9.00E+00	9.00E+00	1.68E+06	3.74E+06	2.59E+06	1.06E+00	2.97E+05	up
572	4-Hydroxyphenylacetic acid	1.52E+02	C8H8O3	[M-H]-	9.00E+00	9.00E+00	9.00E+00	5.91E+05	5.98E+05	5.69E+05	1.06E+00	6.51E+04	up
573	2,6-Dihydroxyacetophenone	1.52E+02	C8H8O3	[M+H]+	3.25E+04	2.11E+04	5.83E+04	2.09E+06	2.00E+06	2.00E+06	1.05E+00	5.44E+01	up
574	Isovanillin*	1.52E+02	C8H8O3	[M+H]+	9.00E+00	9.00E+00	9.00E+00	5.10E+05	1.16E+06	7.70E+05	1.06E+00	9.05E+04	up
575	2,4-Dihydroxybenzoic Acid*	1.54E+02	C7H6O4	[M-H]-	1.34E+05	5.02E+04	7.96E+04	1.85E+07	1.79E+07	1.83E+07	1.06E+00	2.07E+02	up
576	3,4-Dihydroxybenzoic Acid (Protocatechuic acid)*	1.54E+02	C7H6O4	[M-H]-	3.86E+05	8.03E+04	3.30E+05	2.19E+07	2.17E+07	2.16E+07	1.04E+00	8.19E+01	up
577	3,4-Dimethoxyphenol	1.54E+02	C8H10O3	[M+H]+	9.00E+00	9.00E+00	9.00E+00	1.90E+05	2.50E+05	2.66E+05	1.06E+00	2.62E+04	up
578	Hydroxytyrosol	1.54E+02	C8H10O3	[M-H]-	9.00E+00	9.00E+00	9.00E+00	1.85E+05	1.81E+05	1.71E+05	1.06E+00	1.99E+04	up

579	4-Methoxycinnamaldehyde	1.62E+02	C10H10O2	[M+H]+	7.06E+04	5.44E+04	8.08E+04	5.36E+05	4.26E+05	5.12E+05	1.05E+00	7.16E+00	up
580	(E)-3-(3,4-dihydroxyphenyl)acrylaldehyde	1.64E+02	C9H8O3	[M+H]+	1.00E+05	8.09E+04	5.10E+04	2.17E+06	2.18E+06	2.21E+06	1.06E+00	2.83E+01	up
581	α-Hydroxycinnamic acid*	1.64E+02	C9H8O3	[M-H]-	9.00E+00	9.00E+00	9.00E+00	1.71E+06	6.81E+05	1.40E+06	1.06E+00	1.41E+05	up
582	p-Coumaric acid	1.64E+02	C9H8O3	[M+H]+	6.50E+04	5.05E+04	2.87E+04	7.89E+05	7.95E+05	8.43E+05	1.05E+00	1.68E+01	up
583	(E)-m-Coumaric acid*	1.64E+02	C9H8O3	[M-H]-	1.93E+05	1.23E+05	1.03E+05	6.01E+06	5.38E+06	5.43E+06	1.06E+00	4.02E+01	up
584	2-(Formylamino)benzoic Acid	1.65E+02	C8H7NO3	[M-H]-	6.92E+04	5.30E+04	3.86E+04	8.70E+05	8.85E+05	8.14E+05	1.05E+00	1.60E+01	up
585	Terephthalic acid	1.66E+02	C8H6O4	[M-H]-	3.47E+05	3.31E+05	3.74E+05	1.61E+06	2.36E+06	1.74E+06	1.05E+00	5.43E+00	up
586	5,7-Dihydroxy-1(3H)-isobenzofuranone	1.66E+02	C8H6O4	[M-H]-	6.78E+04	6.37E+04	7.07E+04	8.93E+05	9.89E+05	9.17E+05	1.06E+00	1.38E+01	up
587	Ethylparaben	1.66E+02	C9H10O3	[M-H]-	9.00E+00	9.00E+00	9.00E+00	5.43E+06	5.51E+06	5.20E+06	1.06E+00	5.97E+05	up
588	3-(4-Hydroxyphenyl)-propionic acid	1.66E+02	C9H10O3	[M-H]-	3.10E+04	2.99E+04	1.88E+04	1.77E+07	1.70E+07	1.65E+07	1.06E+00	6.42E+02	up
589	2,6-Dimethoxybenzaldehyde	1.66E+02	C9H10O3	[M-H]-	2.68E+04	2.25E+04	1.51E+04	1.11E+07	1.11E+07	1.04E+07	1.06E+00	5.07E+02	up
590	2-Hydroxy-3-phenylpropanoic acid	1.66E+02	C9H10O3	[M-H]-	1.65E+04	1.91E+04	1.97E+04	1.16E+07	1.17E+07	1.11E+07	1.06E+00	6.21E+02	up
591	Ethylsalicylate	1.66E+02	C9H10O3	[M+H]+	9.00E+00	9.00E+00	9.00E+00	1.96E+05	1.59E+05	1.81E+05	1.06E+00	1.99E+04	up
592	Veratraldehyde	1.66E+02	C9H10O3	[M+H]+	2.51E+03	7.77E+03	3.19E+03	4.37E+04	5.25E+04	4.87E+04	1.02E+00	1.08E+01	up
593	2-Amino-3-methoxybenzoic Acid	1.67E+02	C8H9NO3	[M+H]+	1.19E+05	1.43E+05	1.65E+05	1.79E+06	1.32E+06	1.54E+06	1.06E+00	1.09E+01	up
594	Vanillic acid	1.68E+02	C8H8O4	[M-H]-	2.01E+05	1.33E+05	2.04E+05	3.30E+07	3.24E+07	3.07E+07	1.06E+00	1.79E+02	up
595	Homogentisic acid	1.68E+02	C8H8O4	[M-H]-	9.00E+00	9.00E+00	9.00E+00	3.90E+06	3.79E+06	3.41E+06	1.06E+00	4.11E+05	up
596	Phloracetophenone	1.68E+02	C8H8O4	[M-H]-	9.00E+00	9.00E+00	9.00E+00	1.29E+06	1.51E+06	1.42E+06	1.06E+00	1.57E+05	up
597	Caffeic acid	1.80E+02	C9H8O4	[M-H]-	5.14E+04	4.62E+04	5.90E+04	9.22E+06	8.38E+06	8.97E+06	1.06E+00	1.70E+02	up
598	coniferyl alcohol	1.80E+02	C10H12O3	[M-H]-	1.87E+04	3.83E+04	2.28E+04	1.90E+05	1.65E+05	1.51E+05	1.03E+00	6.34E+00	up
599	5-AcetylSalicylic acid	1.80E+02	C9H8O4	[M+H]+	9.00E+00	9.00E+00	9.00E+00	7.97E+05	9.72E+05	8.32E+05	1.06E+00	9.63E+04	up
600	3,4-Dihydroxybenzoic Acid Ethyl Ester (Protocatechuic acid ethyl ester)	1.82E+02	C9H10O4	[M-H]-	9.00E+00	9.00E+00	9.00E+00	3.03E+06	2.96E+06	2.74E+06	1.06E+00	3.23E+05	up
601	Homovanillic acid; 4-Hydroxy-3-methoxyphenylacetic acid	1.82E+02	C9H10O4	[M-H]-	9.00E+00	9.00E+00	9.00E+00	1.13E+05	1.35E+05	1.28E+05	1.06E+00	1.39E+04	up
602	Hydroxyphenyllactic acid	1.82E+02	C9H10O4	[M-H]-	9.00E+00	9.00E+00	9.00E+00	2.09E+06	1.71E+06	1.65E+06	1.06E+00	2.02E+05	up
603	Methyl 2,4-dihydroxyphenylacetate	1.82E+02	C9H10O4	[M-H]-	9.00E+00	9.00E+00	9.00E+00	2.11E+06	1.69E+06	1.80E+06	1.06E+00	2.08E+05	up

604	Syringaldehyde; 4-Hydroxy-3,5-Dimethoxybenzaldehyde	1.82E+02	C9H10O4	[M-H]-	9.00E+00	9.00E+00	9.00E+00	1.05E+05	1.84E+05	1.31E+05	1.06E+00	1.56E+04	up
605	Methyl vanillate	1.82E+02	C9H10O4	[M-H]-	9.00E+00	9.00E+00	9.00E+00	8.48E+04	8.30E+04	7.73E+04	1.06E+00	9.07E+03	up
606	2,4-Dinitrophenol	1.84E+02	C6H4N2O5	[M-H]-	9.00E+00	9.00E+00	9.00E+00	8.49E+04	8.81E+04	9.42E+04	1.06E+00	9.90E+03	up
607	3-O-Methylgallic acid	1.84E+02	C8H8O5	[M-H]-	9.00E+00	9.00E+00	9.00E+00	2.22E+05	2.25E+05	2.21E+05	1.06E+00	2.47E+04	up
608	Noreugenin	1.92E+02	C10H8O4	[M+H]+	9.00E+00	9.00E+00	9.00E+00	1.69E+05	1.63E+05	1.85E+05	1.06E+00	1.91E+04	up
609	p-Coumaric acid ethyl ester	1.92E+02	C11H12O3	[M+H]+	9.00E+00	9.00E+00	9.00E+00	9.86E+05	1.08E+06	1.07E+06	1.06E+00	1.16E+05	up
610	Isoferulic Acid*	1.94E+02	C10H10O4	[M-H]-	5.56E+03	4.68E+03	5.54E+03	3.07E+06	3.20E+06	2.58E+06	1.06E+00	5.60E+02	up
611	Ferulic acid*	1.94E+02	C10H10O4	[M-H]-	7.42E+03	8.41E+03	6.40E+03	2.87E+06	3.08E+06	2.43E+06	1.06E+00	3.77E+02	up
612	3,4-Dimethoxyphenyl acetic acid	1.96E+02	C10H12O4	[M-H]-	3.11E+03	1.31E+03	9.21E+02	1.95E+05	1.78E+05	1.89E+05	1.05E+00	1.05E+02	up
613	Dihydroferulic Acid	1.96E+02	C10H12O4	[M-H]-	9.00E+00	9.00E+00	9.00E+00	1.11E+06	1.01E+06	9.61E+05	1.06E+00	1.14E+05	up
614	Ethyl Vanillate	1.96E+02	C10H12O4	[M+H]+	9.00E+00	9.00E+00	9.00E+00	9.31E+05	9.36E+05	9.80E+05	1.06E+00	1.05E+05	up
615	syringic acid	1.98E+02	C9H10O5	[M-H]-	9.00E+00	9.00E+00	9.00E+00	6.09E+05	5.34E+05	5.86E+05	1.06E+00	6.40E+04	up
616	2,4-Di-Tert-Butylphenol	2.06E+02	C14H22O	[M-H]-	7.70E+05	6.51E+05	7.91E+05	1.95E+06	1.76E+06	1.53E+06	1.04E+00	2.37E+00	up
617	Sinapinaldehyde	2.08E+02	C11H12O4	[M-H]-	3.35E+04	5.57E+04	3.91E+04	1.33E+05	2.49E+05	1.57E+05	1.01E+00	4.19E+00	up
618	Ferulic acid methyl ester	2.08E+02	C11H12O4	[M-H]-	9.00E+00	9.00E+00	9.00E+00	2.92E+07	2.69E+07	2.94E+07	1.06E+00	3.17E+06	up
619	Ethyl caffeate	2.08E+02	C11H12O4	[M-H]-	9.00E+00	9.00E+00	9.00E+00	9.24E+05	9.18E+05	8.95E+05	1.06E+00	1.01E+05	up
620	Ethyl ferulate	2.22E+02	C12H14O4	[M-H]-	9.00E+00	9.00E+00	9.00E+00	1.03E+07	9.64E+06	9.21E+06	1.06E+00	1.08E+06	up
621	Sinapic acid	2.24E+02	C11H12O5	[M-H]-	5.53E+04	2.74E+04	2.14E+04	8.83E+06	9.46E+06	9.04E+06	1.06E+00	2.63E+02	up
622	3,4'-Dihydroxy-3',5'-dimethoxypropiophenone	2.26E+02	C11H14O5	[M+H]+	9.00E+00	9.00E+00	9.00E+00	1.57E+05	1.98E+05	2.22E+05	1.06E+00	2.14E+04	up
623	3-Hydroxy-1-(4-hydroxy-3,5-dimethoxyphenyl)propan-1-one	2.26E+02	C11H14O5	[M+H]+	2.55E+05	3.03E+05	3.59E+05	1.15E+06	2.43E+06	2.25E+06	1.02E+00	6.34E+00	up
624	Stilbostemin B	2.28E+02	C15H16O2	[M+H]+	1.52E+04	1.41E+04	2.30E+04	2.29E+05	2.48E+05	2.85E+05	1.05E+00	1.46E+01	up
625	3,3',5-Trihydroxybibenzyl	2.30E+02	C14H14O3	[M-H]-	9.00E+00	9.00E+00	9.00E+00	3.27E+05	3.51E+05	3.71E+05	1.06E+00	3.88E+04	up
626	Methyl sinapate	2.38E+02	C12H14O5	[M-H]-	9.00E+00	9.00E+00	9.00E+00	1.18E+05	1.36E+05	1.32E+05	1.06E+00	1.43E+04	up
627	3,4'-Dihydroxy-3'-methoxybenzenepentanoic acid	2.40E+02	C12H16O5	[M-H]-	9.00E+00	9.00E+00	9.00E+00	1.39E+04	3.54E+04	1.31E+04	1.06E+00	2.31E+03	up
628	p-Hydroxybenzoyltartaric acid	2.70E+02	C11H10O8	[M-H]-	9.00E+00	9.00E+00	9.00E+00	4.71E+04	6.62E+04	6.42E+04	1.06E+00	6.58E+03	up

629	Benzyl glucoside	2.70E+02	C13H18O6	[M-H]-	9.00E+00	9.00E+00	9.00E+00	1.64E+04	1.21E+04	1.96E+04	1.06E+00	1.78E+03	up
630	4-O-Glucosyl-4-hydroxybenzoic Acid*	3.00E+02	C13H16O8	[M-H]-	3.45E+04	2.09E+04	3.67E+04	1.93E+05	2.31E+05	2.40E+05	1.04E+00	7.21E+00	up
631	Glucosyloxybenzoic Acid*	3.00E+02	C13H16O8	[M-H]-	3.45E+04	2.09E+04	3.67E+04	1.93E+05	2.31E+05	2.40E+05	1.04E+00	7.21E+00	up
632	1-O-Salicyloyl-β-D-glucose	3.00E+02	C13H16O8	[M-H]-	3.45E+04	2.09E+04	3.67E+04	1.93E+05	2.31E+05	2.40E+05	1.04E+00	7.21E+00	up
633	Doitungbiphenyl A	3.00E+02	C18H20O4	[M-H]-	9.00E+00	9.00E+00	9.00E+00	7.63E+05	8.07E+05	5.54E+05	1.06E+00	7.87E+04	up
634	3-methoxy-5-hydroxy-1-O-β-D-glucopyranoside	3.02E+02	C13H18O8	[M-H]-	9.00E+00	9.00E+00	9.00E+00	5.83E+04	6.01E+04	6.24E+04	1.06E+00	6.70E+03	up
635	Feruloylmalic acid	3.10E+02	C14H14O8	[M-H]-	9.00E+00	9.00E+00	9.00E+00	2.71E+05	2.60E+05	2.54E+05	1.06E+00	2.91E+04	up
636	1-O-Galloyl-rhamnose	3.16E+02	C13H16O9	[M+H]+	2.98E+04	4.22E+04	5.09E+04	5.81E+05	7.15E+05	7.12E+05	1.05E+00	1.63E+01	up
637	Protocatechuic acid-4-O-glucoside	3.16E+02	C13H16O9	[M-H]-	1.60E+06	1.34E+06	1.69E+06	6.18E+05	6.59E+05	6.37E+05	1.05E+00	4.14E-01	down
638	5-(2-Hydroxyethyl)-2-O-glucosylphenol	3.16E+02	C14H20O8	[M-H]-	9.00E+00	9.00E+00	9.00E+00	1.53E+05	1.14E+05	1.31E+05	1.06E+00	1.47E+04	up
639	Trans-5-O-(p-Coumaroyl)shikimate	3.20E+02	C16H16O7	[M+H]+	9.00E+00	9.00E+00	9.00E+00	2.81E+05	2.07E+05	2.35E+05	1.06E+00	2.68E+04	up
640	5-Galloylshikimic acid	3.26E+02	C14H14O9	[M-H]-	9.00E+00	9.00E+00	9.00E+00	1.36E+05	1.59E+05	1.50E+05	1.06E+00	1.65E+04	up
641	1-O-p-Coumaroyl-β-D-glucose	3.26E+02	C15H18O8	[M-H]-	2.65E+06	2.01E+06	1.67E+06	9.00E+00	9.00E+00	9.00E+00	1.06E+00	4.27E-06	down
642	Phenylpropionic acid-O-β-D-glucopyranoside*	3.26E+02	C15H18O8	[M-H]-	9.00E+00	9.00E+00	9.00E+00	2.97E+04	4.55E+04	3.52E+04	1.06E+00	4.09E+03	up
643	Demethyl coniferin	3.28E+02	C15H20O8	[M-H]-	9.00E+00	9.00E+00	9.00E+00	4.52E+05	2.91E+05	3.64E+05	1.06E+00	4.10E+04	up
644	3-Hydroxy-4-isopropylbenzylalcohol-3-O-glucoside	3.28E+02	C16H24O7	[M-H]-	7.67E+05	7.71E+05	8.54E+05	3.36E+06	3.49E+06	3.55E+06	1.06E+00	4.35E+00	up
645	5-Glucosyloxy-2-Hydroxybenzoic Acid methyl ester	3.30E+02	C14H18O9	[M-H]-	2.85E+04	1.54E+04	1.95E+04	6.45E+05	6.97E+05	6.47E+05	1.06E+00	3.14E+01	up
646	2,4,6-trihydroxy-acetophenone-4-O-b-D-glucopyranoside	3.30E+02	C14H18O9	[M-H]-	1.51E+06	1.58E+06	1.85E+06	6.13E+05	5.57E+05	5.78E+05	1.05E+00	3.54E-01	down
647	1-O-Vanilloyl-D-Glucose	3.30E+02	C14H18O9	[M-H]-	9.00E+00	9.00E+00	9.00E+00	6.59E+05	6.42E+05	7.18E+05	1.06E+00	7.48E+04	up
648	1-O-Caffeoyl-β-D-glucose	3.42E+02	C15H18O9	[M-H]-	1.40E+07	1.86E+07	1.79E+07	4.13E+04	9.12E+04	6.65E+04	1.06E+00	3.94E-03	down
649	6-O-Caffeoyl-D-glucose	3.42E+02	C15H18O9	[M-H]-	3.14E+06	3.40E+06	2.98E+06	3.88E+05	5.47E+05	4.71E+05	1.06E+00	1.48E-01	down
650	Syringaldehyde-4-O-glucoside	3.44E+02	C15H20O9	[M-H]-	8.80E+05	8.23E+05	7.54E+05	4.69E+06	4.61E+06	4.61E+06	1.06E+00	5.66E+00	up
651	Dihydrocaffeoylglucose	3.44E+02	C15H20O9	[M-H]-	6.09E+05	8.24E+05	7.42E+05	4.65E+06	4.54E+06	4.81E+06	1.06E+00	6.43E+00	up
652	Chlorogenic acid (3-O-caffeoylquinic acid)*	3.54E+02	C16H18O9	[M-H]-	1.45E+06	1.58E+06	1.57E+06	9.00E+00	9.00E+00	9.00E+00	1.06E+00	5.87E-06	down

653	1-caffeoylquinic acid*	3.54E+02	C16H18O9	[M-H]-	9.00E+00	9.00E+00	9.00E+00	1.22E+05	1.09E+05	1.19E+05	1.06E+00	1.29E+04	up
654	3-O-Feruloylquinic acid*	3.68E+02	C17H20O9	[M+H]+	2.29E+05	2.91E+05	2.63E+05	4.70E+05	6.33E+05	5.49E+05	1.02E+00	2.11E+00	up
655	3-Prenyl-4-O-glucosyloxy-4-hydroxybenzoic acid	3.68E+02	C18H24O8	[M-H]-	9.00E+00	9.00E+00	9.00E+00	1.35E+05	1.23E+05	1.66E+05	1.06E+00	1.57E+04	up
656	Feruloyl syringic acid	3.74E+02	C19H18O8	[M+H]+	7.84E+04	1.09E+05	9.60E+04	5.93E+05	7.50E+05	6.55E+05	1.05E+00	7.05E+00	up
657	D-Threo-guaiacylglycerol-7-O-β-D-glucoside	3.76E+02	C16H24O10	[M-H]-	2.65E+04	3.10E+04	2.94E+04	8.72E+04	1.34E+05	1.27E+05	1.04E+00	4.01E+00	up
658	ethyl rosmarinate	3.88E+02	C20H20O8	[M+H]+	1.70E+05	1.64E+05	2.20E+05	2.94E+06	3.28E+06	3.24E+06	1.06E+00	1.71E+01	up
659	5'-Glucosyloxyjasmanic acid	3.88E+02	C18H28O9	[M-H]-	2.93E+05	2.55E+05	2.39E+05	1.15E+05	1.30E+05	1.24E+05	1.04E+00	4.69E-01	down
660	4-O-Sinapoylquinic acid	3.98E+02	C18H22O10	[M+H]+	4.53E+04	4.35E+04	4.09E+04	1.47E+05	2.06E+05	1.63E+05	1.05E+00	3.98E+00	up
661	Benzyl-(2''-O-xylosyl)glucoside	4.02E+02	C18H26O10	[M-H]-	4.38E+05	4.65E+05	5.09E+05	1.79E+06	1.93E+06	1.68E+06	1.06E+00	3.82E+00	up
662	Echinacoside	7.86E+02	C35H46O20	[M-H]-	2.92E+05	3.25E+05	3.21E+05	3.71E+04	5.87E+04	2.60E+04	1.04E+00	1.30E-01	down
**Quinones**													
663	Anthraquinone-2-carboxylic acid	2.52E+02	C15H8O4	[M-H]-	9.00E+00	9.00E+00	9.00E+00	3.51E+05	3.55E+05	3.37E+05	1.06E+00	3.86E+04	up
664	5-hydroxy-anthraquinone-2-carboxylic acid	2.68E+02	C15H8O5	[M-H]-	9.00E+00	9.00E+00	9.00E+00	1.43E+04	1.24E+04	1.47E+04	1.06E+00	1.53E+03	up
665	Emodin-1-methyl ether	2.84E+02	C16H12O5	[M-H]-	9.00E+00	9.00E+00	9.00E+00	9.49E+04	9.41E+04	9.22E+04	1.06E+00	1.04E+04	up
666	Aloe emodin-1-O-glucoside	4.32E+02	C21H20O10	[M-H]-	9.00E+00	9.00E+00	9.00E+00	2.70E+04	1.04E+04	3.24E+04	1.06E+00	2.59E+03	up
667	HydroxyAloe-Emodin-8-O-glucoside	4.48E+02	C21H20O11	[M-H]-	9.00E+00	9.00E+00	9.00E+00	2.46E+05	2.43E+05	2.68E+05	1.06E+00	2.81E+04	up
668	Emodin-8-O-(6''-O-acetyl)glucoside	4.74E+02	C23H22O11	[M-H]-	9.00E+00	9.00E+00	9.00E+00	1.22E+05	9.21E+04	1.02E+05	1.06E+00	1.17E+04	up
669	Physcion-8-0-(6-acetyl)-glucoside	4.88E+02	C24H24O11	[M-H]-	3.22E+04	1.38E+04	2.34E+04	3.57E+05	3.38E+05	3.23E+05	1.05E+00	1.47E+01	up
**Terpenoids**													
670	Jasminoside B	3.46E+02	C16H26O8	[M+H]+	5.20E+04	3.34E+04	4.86E+04	2.69E+05	3.12E+05	2.92E+05	1.05E+00	6.51E+00	up
671	sweroside	3.58E+02	C16H22O9	[M+H]+	8.34E+04	8.51E+04	7.45E+04	2.36E+05	3.31E+05	3.27E+05	1.05E+00	3.68E+00	up
672	7-Deoxyloganic acid	3.60E+02	C16H24O9	[M-H]-	1.64E+04	1.88E+04	1.38E+04	1.15E+05	1.29E+05	1.18E+05	1.06E+00	7.39E+00	up
673	Geniposide	3.88E+02	C17H24O10	[M+H]+	9.31E+04	1.15E+05	1.38E+05	6.86E+05	8.95E+05	7.93E+05	1.05E+00	6.85E+00	up
674	Obacunone	4.54E+02	C26H30O7	[M+H]+	1.70E+04	5.18E+03	1.21E+04	2.01E+05	1.59E+05	1.65E+05	1.03E+00	1.53E+01	up
675	Limonin	4.70E+02	C26H30O8	[M+H]+	8.18E+06	4.71E+06	5.97E+06	3.66E+07	3.76E+07	3.72E+07	1.05E+00	5.90E+00	up
676	Melianone	4.70E+02	C30H46O4	[M+H]+	7.07E+06	4.05E+06	5.54E+06	3.21E+07	2.89E+07	3.23E+07	1.04E+00	5.60E+00	up
677	Obacunoic acid	4.72E+02	C26H32O8	[M+H]+	3.97E+04	1.17E+04	1.85E+04	6.87E+05	6.65E+05	7.11E+05	1.04E+00	2.95E+01	up

678	Deacetylnomilin	4.72E+02	C26H32O8	[M+H]+	9.62E+05	6.39E+05	7.77E+05	1.57E+06	1.54E+06	1.69E+06	1.01E+00	2.02E+00	up
679	Deacetylnomilinic acid	4.90E+02	C26H34O9	[M+H]+	2.62E+05	1.73E+05	1.77E+05	3.16E+06	3.34E+06	3.71E+06	1.06E+00	1.67E+01	up
680	Nomilinic acid	5.32E+02	C28H36O10	[M-H]-	1.65E+05	9.05E+04	1.59E+05	1.27E+07	1.17E+07	1.24E+07	1.06E+00	8.90E+01	up
681	2α,3β,19α-Trihydroxyurs-12-en-23,28-dioic acid-28-O-glucoside	6.80E+02	C36H56O12	[M-H]-	2.29E+04	3.10E+04	3.49E+04	9.00E+00	9.00E+00	9.00E+00	1.06E+00	3.04E-04	down
**Others**													
682	3-Methylbenzaldehyde*	1.20E+02	C8H8O	[M-H]-	9.00E+00	9.00E+00	9.00E+00	4.71E+04	1.93E+04	4.13E+04	1.06E+00	3.99E+03	up
683	4-Methylbenzaldehyde*	1.20E+02	C8H8O	[M-H]-	9.00E+00	9.00E+00	9.00E+00	4.25E+04	3.22E+04	4.11E+04	1.06E+00	4.29E+03	up
684	Acetylpyrazine	1.22E+02	C6H6N2O	[M+H]+	9.00E+00	9.00E+00	9.00E+00	2.80E+05	3.30E+05	2.74E+05	1.06E+00	3.28E+04	up
685	Nicotinic acid (Vitamin B3)*	1.23E+02	C6H5NO2	[M+H]+	4.96E+04	3.79E+04	5.34E+04	5.24E+06	5.44E+06	5.85E+06	1.06E+00	1.17E+02	up
686	5-hydroxymethylfurfural	1.26E+02	C6H6O3	[M+H]+	9.00E+00	9.00E+00	9.00E+00	1.22E+06	1.75E+06	1.39E+06	1.06E+00	1.62E+05	up
687	4-Guanidinobutanal	1.29E+02	C5H11N3O	[M+H]+	1.34E+06	1.49E+06	1.36E+06	4.79E+06	4.89E+06	5.07E+06	1.06E+00	3.52E+00	up
688	2-Benzoxazolinone	1.35E+02	C7H5NO2	[M+H]+	9.00E+00	9.00E+00	9.00E+00	3.68E+05	4.06E+05	3.72E+05	1.06E+00	4.24E+04	up
689	D-Threonic Acid	1.36E+02	C4H8O5	[M-H]-	1.68E+05	1.50E+05	2.12E+05	6.57E+05	6.92E+05	6.94E+05	1.05E+00	3.85E+00	up
690	5-hydroxymaltol	1.42E+02	C6H6O4	[M+H]+	9.00E+00	9.00E+00	9.00E+00	1.41E+05	1.73E+05	1.49E+05	1.06E+00	1.71E+04	up
691	D-Arabinono-1,4-lactone	1.48E+02	C5H8O5	[M-H]-	9.00E+00	9.00E+00	9.00E+00	2.44E+07	2.35E+07	2.55E+07	1.06E+00	2.72E+06	up
692	2-Dehydro-3-deoxy-L-arabinonate	1.48E+02	C5H8O5	[M-H]-	3.62E+05	4.00E+05	3.55E+05	8.52E+06	7.88E+06	7.02E+06	1.06E+00	2.10E+01	up
693	Mesitaldehyde	1.48E+02	C10H12O	[M+H]+	9.00E+00	9.00E+00	9.00E+00	1.86E+05	2.34E+05	2.01E+05	1.06E+00	2.30E+04	up
694	Benzylacetone	1.48E+02	C10H12O	[M+H]+	9.00E+00	9.00E+00	9.00E+00	1.80E+05	1.80E+05	1.86E+05	1.06E+00	2.02E+04	up
695	2',4'-Dimethylacetophenone	1.48E+02	C10H12O	[M+H]+	9.00E+00	9.00E+00	9.00E+00	1.86E+05	2.34E+05	2.01E+05	1.06E+00	2.30E+04	up
696	L-Arabitol	1.52E+02	C5H12O5	[M-H]-	9.00E+00	9.00E+00	9.00E+00	2.71E+05	2.48E+05	2.09E+05	1.06E+00	2.70E+04	up
697	Xylitol	1.52E+02	C5H12O5	[M-H]-	9.00E+00	9.00E+00	9.00E+00	1.06E+06	1.07E+06	9.97E+05	1.06E+00	1.16E+05	up
698	2-Hydroxy-4-methoxybenzaldehyde	1.52E+02	C8H8O3	[M+H]+	9.00E+00	9.00E+00	9.00E+00	4.10E+04	5.79E+04	5.66E+04	1.06E+00	5.76E+03	up
699	Orotic acid	1.56E+02	C5H4N2O4	[M-H]-	1.40E+05	1.56E+05	1.96E+05	9.00E+00	9.00E+00	9.00E+00	1.06E+00	5.49E-05	down
700	1,6-anhydro-β-D-glucose	1.62E+02	C6H10O5	[M-H]-	9.81E+05	1.07E+06	9.84E+05	4.10E+04	6.42E+04	3.93E+04	1.06E+00	4.77E-02	down
701	5-Hydroxy-1-tetralone	1.62E+02	C10H10O2	[M+H]+	9.00E+00	9.00E+00	9.00E+00	4.66E+05	4.19E+05	4.34E+05	1.06E+00	4.89E+04	up
702	4-hydroxyphenyl acrylaldehyde	1.64E+02	C9H8O3	[M+H]+	4.87E+04	8.76E+04	2.83E+04	1.15E+06	8.68E+05	8.36E+05	1.04E+00	1.73E+01	up

703	Rhamnose	1.64E+02	C6H12O5	[M-H]-	9.00E+00	9.00E+00	9.00E+00	1.51E+05	1.42E+05	1.62E+05	1.06E+00	1.68E+04	up
704	L-Fucose	1.64E+02	C6H12O5	[M-H]-	9.00E+00	9.00E+00	9.00E+00	1.13E+05	1.09E+05	9.13E+04	1.06E+00	1.16E+04	up
705	Pyridoxine	1.69E+02	C8H11NO3	[M+H]+	4.10E+05	3.63E+05	4.41E+05	1.68E+06	1.59E+06	1.58E+06	1.06E+00	3.99E+00	up
706	D-Glucurono-6,3-lactone	1.76E+02	C6H8O6	[M-H]-	2.00E+05	2.18E+05	1.93E+05	6.21E+05	4.92E+05	4.81E+05	1.04E+00	2.61E+00	up
707	L-Ascorbic acid (Vitamin C)*	1.76E+02	C6H8O6	[M-H]-	2.36E+06	3.62E+06	3.29E+06	1.09E+07	8.10E+06	1.14E+07	1.02E+00	3.27E+00	up
708	5,7-Dihydroxychromone	1.78E+02	C9H6O4	[M-H]-	9.00E+00	9.00E+00	9.00E+00	1.14E+05	1.77E+05	1.45E+05	1.06E+00	1.62E+04	up
709	D-Mannose*	1.80E+02	C6H12O6	[M-H]-	3.78E+07	3.92E+07	3.59E+07	1.83E+06	2.43E+06	2.11E+06	1.06E+00	5.64E-02	down
710	D-Galactose*	1.80E+02	C6H12O6	[M-H]-	2.57E+07	2.80E+07	2.77E+07	1.57E+06	1.83E+06	1.56E+06	1.06E+00	6.10E-02	down
711	D-Glucose*	1.80E+02	C6H12O6	[M-H]-	3.65E+07	3.72E+07	3.26E+07	2.44E+06	3.06E+06	2.87E+06	1.06E+00	7.88E-02	down
712	D-Fructose*	1.80E+02	C6H12O6	[M-H]-	3.65E+07	3.59E+07	3.36E+07	2.55E+06	3.05E+06	2.83E+06	1.06E+00	7.95E-02	down
713	Dulcitol	1.82E+02	C6H14O6	[M-H]-	3.05E+04	3.20E+04	2.53E+04	1.42E+05	1.04E+05	9.12E+04	1.04E+00	3.83E+00	up
714	4-Pyridoxic acid	1.83E+02	C8H9NO4	[M+H]+	4.17E+04	4.32E+04	4.04E+04	1.01E+06	9.81E+05	1.22E+06	1.06E+00	2.56E+01	up
715	2,6-Dimethyl-7-octene-2,3,6-triol	1.88E+02	C10H20O3	[M-H]-	2.43E+05	1.43E+05	1.79E+05	6.94E+05	7.27E+05	6.87E+05	1.04E+00	3.73E+00	up
716	D-Galacturonic acid*	1.94E+02	C6H10O7	[M-H]-	6.42E+03	4.91E+03	6.84E+03	1.21E+06	1.22E+06	1.12E+06	1.06E+00	1.96E+02	up
717	D-Glucoronic acid*	1.94E+02	C6H10O7	[M-H]-	5.52E+03	1.08E+04	9.68E+03	1.26E+06	1.16E+06	1.21E+06	1.06E+00	1.39E+02	up
718	D-Erythrose-4-phosphate	2.00E+02	C4H9O7P	[M-H]-	1.64E+04	9.07E+03	1.10E+04	1.59E+06	1.18E+06	1.45E+06	1.06E+00	1.16E+02	up
719	N,N'-dicyclohexylcarbodiimide	2.06E+02	C13H22N2	[M-H]-	7.41E+05	6.04E+05	7.81E+05	2.01E+06	1.75E+06	1.57E+06	1.03E+00	2.51E+00	up
720	D-Saccharic acid*	2.10E+02	C6H10O8	[M-H]-	2.77E+05	3.67E+05	3.90E+05	3.54E+06	2.95E+06	2.90E+06	1.06E+00	9.07E+00	up
721	D-Galactaric acid*	2.10E+02	C6H10O8	[M-H]-	2.94E+06	2.89E+06	2.76E+06	3.97E+05	4.85E+05	4.64E+05	1.06E+00	1.57E-01	down
722	D-Pantothenic Acid	2.19E+02	C9H17NO5	[M-H]-	1.65E+06	1.59E+06	1.78E+06	4.58E+06	4.49E+06	4.35E+06	1.06E+00	2.67E+00	up
723	Z-6-Hydroxy-7-methoxydihydroligustilide	2.36E+02	C13H16O4	[M+H]+	5.96E+04	8.41E+04	8.40E+04	2.47E+06	2.50E+06	2.33E+06	1.06E+00	3.21E+01	up
724	Pyridoxine-5'-phosphate	2.49E+02	C8H12NO6P	[M+H]+	9.00E+00	9.00E+00	9.00E+00	8.53E+05	7.37E+05	6.95E+05	1.06E+00	8.46E+04	up
725	D-Glucosamine 1-phosphate	2.59E+02	C6H14NO8P	[M-H]-	7.35E+04	7.59E+04	9.77E+04	1.24E+06	1.50E+06	1.45E+06	1.06E+00	1.69E+01	up
726	Glucose-1-phosphate*	2.60E+02	C6H13O9P	[M-H]-	2.17E+06	2.68E+06	2.63E+06	9.00E+00	9.00E+00	9.00E+00	1.06E+00	3.61E-06	down
727	D-Fructose 6-phosphate*	2.60E+02	C6H13O9P	[M-H]-	4.09E+06	3.49E+06	2.76E+06	4.05E+04	7.37E+04	2.01E+05	1.03E+00	3.05E-02	down
728	D-Glucose 6-phosphate*	2.60E+02	C6H13O9P	[M-H]-	2.23E+06	2.46E+06	2.39E+06	9.00E+00	9.00E+00	9.00E+00	1.06E+00	3.82E-06	down
729	3,5-Dihydroxy-2,4-dimethoxy-9H-fluoren-9-one	2.72E+02	C15H12O5	[M+H]+	9.00E+00	9.00E+00	9.00E+00	9.22E+05	9.95E+05	9.41E+05	1.06E+00	1.06E+05	up

730	Glucarate O-Phosphoric acid	2.90E+02	C6H11PO11	[M-H]-	1.56E+06	1.84E+06	3.19E+06	7.72E+06	8.44E+06	8.82E+06	1.01E+00	3.79E+00	up
731	3,5,7,4'-Tetrahydroxy-Coumaronochromone	3.02E+02	C15H10O7	[M+H]+	3.18E+04	2.78E+04	1.79E+04	2.44E+05	2.80E+05	2.95E+05	1.05E+00	1.06E+01	up
732	3,4-dihydroxy-allylbenzene-3-O-β-D-glucopyranoside	3.12E+02	C15H20O7	[M-H]-	7.43E+04	6.10E+04	4.92E+04	3.72E+05	4.24E+05	4.20E+05	1.05E+00	6.59E+00	up
733	N-benzoyl-2-aminoethyl-β-D-glucopyranoside	3.27E+02	C15H21NO7	[M+H]+	9.47E+04	6.75E+04	1.03E+05	1.36E+06	1.58E+06	1.56E+06	1.06E+00	1.69E+01	up
734	Aromatide	3.27E+02	C19H21NO4	[M+H]+	2.44E+04	1.88E+04	2.23E+04	4.04E+07	3.61E+07	3.83E+07	1.06E+00	1.75E+03	up
735	2-Amino-1,3-eicosanediol	3.29E+02	C20H43NO2	[M+H]+	9.17E+05	8.59E+05	7.03E+05	8.35E+06	8.55E+06	7.07E+06	1.06E+00	9.67E+00	up
736	Isomaltulose*	3.42E+02	C12H22O11	[M-H]-	6.32E+06	9.67E+06	6.98E+06	8.70E+04	6.57E+04	7.33E+04	1.06E+00	9.83E-03	down
737	D-Maltose*	3.42E+02	C12H22O11	[M-H]-	4.71E+06	8.33E+06	5.65E+06	7.74E+04	8.82E+04	7.77E+04	1.06E+00	1.30E-02	down
738	Lactobiose*	3.42E+02	C12H22O11	[M-H]-	8.83E+06	8.87E+06	8.13E+06	1.30E+05	9.25E+04	1.34E+05	1.06E+00	1.38E-02	down
739	D-Trehalose*	3.42E+02	C12H22O11	[M-H]-	2.60E+06	9.52E+06	3.65E+06	2.80E+04	1.85E+04	1.99E+04	1.05E+00	4.21E-03	down
740	Melibiose*	3.42E+02	C12H22O11	[M-H]-	2.25E+06	2.56E+06	2.01E+06	5.69E+04	6.31E+04	6.10E+04	1.06E+00	2.65E-02	down
741	D-Sucrose*	3.42E+02	C12H22O11	[M-H]-	9.69E+06	9.52E+06	1.15E+07	3.60E+04	2.72E+04	3.91E+04	1.06E+00	3.34E-03	down
742	Galactinol*	3.42E+02	C12H22O11	[M-H]-	2.45E+07	2.36E+07	2.00E+07	1.76E+05	1.35E+05	1.54E+05	1.06E+00	6.82E-03	down
743	4-Pyridoxic acid-O-glucoside	3.45E+02	C14H19NO9	[M+H]+	1.46E+04	1.61E+04	2.15E+04	3.24E+05	2.90E+05	2.34E+05	1.06E+00	1.62E+01	up
744	α-Conidendrin	3.56E+02	C20H20O6	[M+H]+	2.42E+04	2.86E+04	2.09E+04	3.06E+05	2.67E+05	2.70E+05	1.06E+00	1.14E+01	up
745	Bergaptol-O-β-D-glucoside	3.64E+02	C17H16O9	[M+H]+	2.61E+05	2.69E+05	3.96E+05	6.45E+05	7.77E+05	7.22E+05	1.01E+00	2.32E+00	up
746	3-Oxo-α-ionol-β-D-glucoside	3.70E+02	C19H30O7	[M+H]+	4.56E+04	3.05E+04	4.52E+04	1.96E+05	2.32E+05	2.12E+05	1.05E+00	5.28E+00	up
747	Citroside A	3.86E+02	C19H30O8	[M+H]+	1.09E+05	1.31E+05	1.36E+05	1.15E+06	1.55E+06	1.33E+06	1.06E+00	1.07E+01	up
748	Austroside B	3.86E+02	C19H30O8	[M+H]+	1.65E+05	1.71E+05	1.83E+05	1.29E+06	1.65E+06	1.36E+06	1.06E+00	8.27E+00	up
749	Evodol	4.84E+02	C26H28O9	[M+H]+	9.00E+00	9.00E+00	9.00E+00	5.44E+05	6.27E+05	5.07E+05	1.06E+00	6.21E+04	up
750	Rutaevin	4.86E+02	C26H30O9	[M+H]+	9.00E+00	9.00E+00	9.00E+00	1.58E+05	1.12E+05	8.92E+04	1.06E+00	1.33E+04	up
751	12α-Hydroxyevodol	5.00E+02	C26H28O10	[M-H]-	9.00E+00	9.00E+00	9.00E+00	1.77E+05	1.83E+05	1.45E+05	1.06E+00	1.87E+04	up
752	Dehydrodiconiferyl alcohol-4-O-glucoside	5.20E+02	C26H32O11	[M+H]+	1.31E+06	1.48E+06	1.38E+06	1.75E+05	1.83E+05	1.70E+05	1.06E+00	1.27E-01	down

Note: The differential metabolites are screened based on VIP >1 and p <0.05. Metabolites are considered to be up-regulated with fold change value >2 and considered to be down-regulated with fold change <0.5.

*Isomer metabolites.
